# Evaluation of *N*-alkyl isatins and indoles as acetylcholinesterase and butyrylcholinesterase inhibitors

**DOI:** 10.1080/14756366.2023.2286935

**Published:** 2023-12-07

**Authors:** Kaitlyn N. Alcorn, Isabelle A. Oberhauser, Matthew D. Politeski, Todd J. Eckroat

**Affiliations:** School of Science, Penn State Erie, The Behrend College, Erie, PA, USA

**Keywords:** Isatin, indole, acetylcholinesterase, butyrylcholinesterase

## Abstract

Two series of *N*-alkyl isatins and *N*-alkyl indoles varying in size of the alkyl group were synthesised and evaluated for inhibition of acetylcholinesterase (AChE) and butyrylcholinesterase (BChE). Among the *N*-alkyl isatins **4a**-**j**, the addition of the *N*-alkyl group improved inhibition potency towards AChE and BChE compared to isatin. Selectivity towards inhibition of BChE was observed, and the increase in size of the *N*-alkyl group positively correlated to improved inhibition potency. The most potent inhibitor for BChE was **4i** (IC_50_ = 3.77 µM, 22-fold selectivity for BChE over AChE). N-alkyl indoles **5a**-**j** showed similar inhibition of AChE, the most potent being **5g** (IC_50_ = 35.0 µM), but **5a**-**j** lost activity towards BChE. This suggests an important role of the 3-oxo group on isatin for BChE inhibition, and molecular docking of **4i** with human BChE indicates a key hydrogen bond between this group and Ser198 and His438 of the BChE catalytic triad.

## Graphical Abstract

**Figure:**
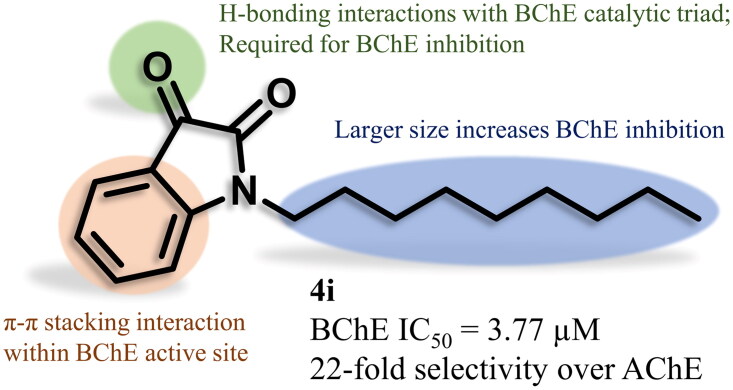

## Introduction

Acetylcholinesterase (AChE, EC 3.1.1.7) and butyrylcholinesterase (BChE, EC 3.1.1.8) are well-studied enzymes with structural, mechanistic, and physiological similarities^1^. Both enzymes contain three important structural features: a catalytic active site (CAS), gorge region, and peripheral anionic site (PAS). The CAS of each enzyme contains the Ser-His-Glu catalytic triad common to serine esterases, as well as anionic, oxyanion hole, and acyl pocket subsites. The gorge is about 20 Å long and connects the CAS at the enzyme interior to the PAS at the enzyme surface. Aromatic amino acids are prominent in the CAS, gorge, and PAS of both AChE and BChE. However, key amino acid substitutions in BChE relative to AChE are worth mentioning. First, the replacement of two Phe residues with Leu and Val in the acyl pocket allows BChE to bind larger substrates. Additionally, several aromatic residues are substituted with hydrophobic residues in the lining of the BChE gorge^2–5^.

AChE and BChE are both well-known enzymatic targets for treatment of Alzheimer’s disease (AD), and AChE and BChE inhibitors (AChEI and BChEI, respectively) have long been of interest. Under normal conditions, AChE hydrolyses acetylcholine (ACh) to regulate neurotransmission^6^. Inhibition of this enzyme boosts ACh levels to treat symptoms of AD, and it may also affect other AD etiologies^7–9^. Inhibition of BChE, which shows increased levels in advanced stages of AD, can have a similar beneficial effect^10^^,^^11^. Our lab has an ongoing interest in exploring new or understudied chemical scaffolds as dual inhibitors of AChE and BChE or selective inhibitors of one or the other.

**Scheme 1.**
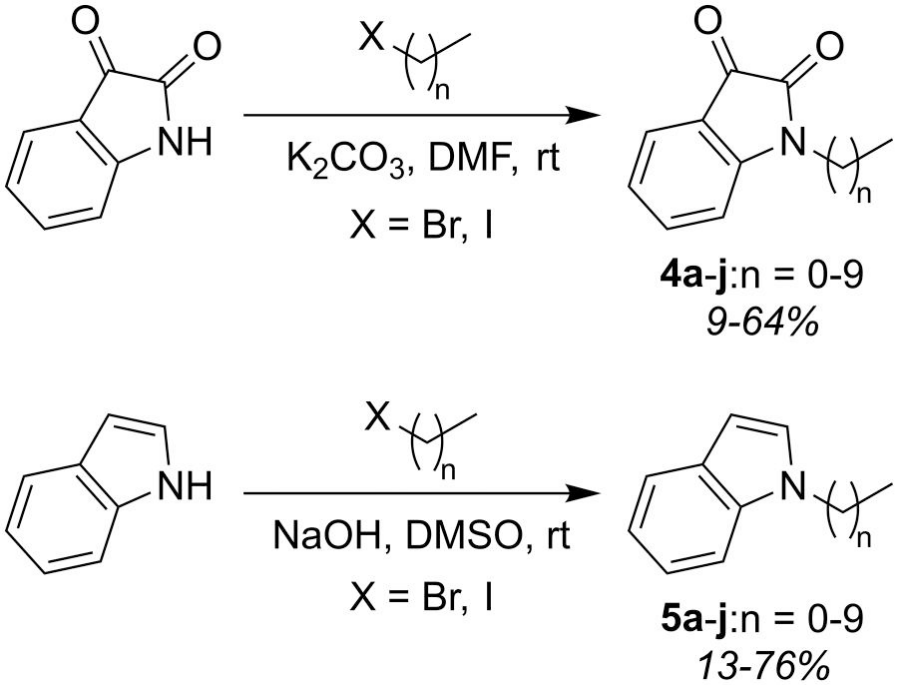
Synthesis of *N*-alkyl isatins **4a**-**j** and *N*-alkyl indoles **5a**-**j**.

**Figure 1.**
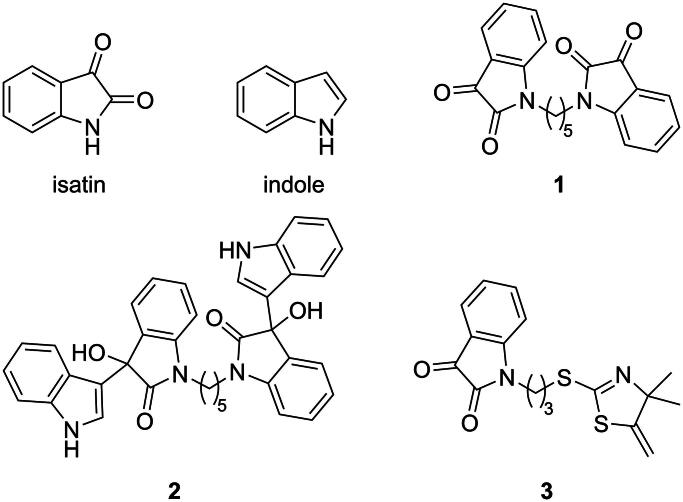
Structures of isatin, indole, and previously investigated isatin/indole-based AChEI and BChEI.

Nitrogen-containing heterocycles show diverse biological activities in the literature, and they are well-known scaffolds for the design of AChEI and BChEI, which has recently been reviewed^12^^,^^13^. Isatin (indoline-2,3-dione) and indole (Figure 1), and derivatives thereof, are no exception with various reports of inhibition of AChE and BChE^14–17^. We recently reported isatin dimer **1** and 3-indolyl-3-hydroxy-2-oxindole dimer **2** as selective BChEI (IC_50_ = 7.56 and 4.49 µM, respectively) (Figure 1).^18^ Prior, we reported on the AChE inhibition of isatin-linked thiazoline-2-thiol **3** (IC_50_ = 18.2 µM) (Figure 1).^19^ In the course of this previous work, we noted that 1-butylindoline-2,3-dione (**4d**, see Scheme 1), in which the nitrogen of isatin has been substituted with a butyl group, showed weak inhibition of AChE (IC_50_ = 540 µM). Compound **4d** was prepared in the context of using it as a control for inhibition by comparison with **3** and was expected to be inactive. However, the weak inhibition caught our attention, and further investigation of **4d** showed selectivity for BChE inhibition (IC_50_ = 73.7 µM) (previously unpublished). Naturally, this led us to wonder about the structure-activity relationship (SAR) between size of the alkyl group and inhibition of AChE and/or BChE. To further complement this *N*-alkyl SAR investigation, we also chose to investigate the role of the 2-oxo and 3-oxo groups of isatin by making a comparable indole series. Despite other biological activities being known for *N*-alkyl isatins and indoles, to our knowledge, there is no comprehensive report in the literature addressing the effect of N-alkylation of isatin or indole on the ability of these compounds to act as AChEI and BChEI. Herein, we report investigations into this matter.

## Materials and methods

### General

Consumable chemicals for synthesis and biochemical testing were purchased from Sigma-Aldrich (St. Louis, MO), VWR International (Radnor, PA), Beantown Chemical (Hudson, NH), AK Scientific (Union City, CA), and Tokyo Chemical Industry (Tokyo, Japan). A Bruker Avance 400 spectrometer was used to collect all ^1^H and ^13^C NMR spectra, and a Thermo Scientific™ Q Exactive™ Hybrid Quadrupole-Orbitrap Mass Spectrometer was used to collect HRMS. UV-Vis absorbance in enzymatic assay was monitored on a Dynex Technologies Opsys MR™ Microplate Reader using flat-bottomed 96-well plates (VWR International).

### General procedure for synthesis of *N*-alkyl isatins (4a-j)

Isatin (200 mg, 1.36 mmol), iodomethane (1.5 eq) or 1-bromoalkane (1.5 eq), K_2_CO_3_ (2 eq), and DMF (3 ml) were combined and stirred at rt for 24 h. The reaction mixture was then diluted with water (100 ml) and extracted with DCM (3 × 15 ml). The combined organic layers were washed with brine (50 ml), dried (MgSO_4_), and concentrated under reduced pressure to afford the crude product, which was then purified by flash column chromatography (SiO_2_; 3:1/hexanes:EtOAc) to yield the desired product.

#### 1-Methylindoline-2,3-dione (4a)

Yield = 63% as an orange solid; ^1^H NMR (DMSO-*d*_6_, 400 MHz) δ 7.67 (td, 1H, *J*_1_ = 7.9 Hz, *J*_2_ = 1.1 Hz Ar-H), 7.54 (d, 1H, *J* = 7.3 Hz, Ar-H), 7.13 (m, 2H, Ar-H), 3.14 (s, 3H, -CH_3_); ^13^C NMR (DMSO-*d*_6_, 100 MHz) δ 183.4 (C = O), 158.2 (C = O), 151.4 (Ar-C), 138.2 (Ar-C), 124.2 (Ar-C), 123.2 (Ar-C), 117.4 (Ar-C), 110.6 (Ar-C), 26.0 (-CH_3_); HRMS (ESI) *m/z* calcd for C_9_H_7_NO_2_: 161.0471, found 162.0550 [M + H]^+^.

#### 1-Ethylindoline-2,3-dione (4b)

Yield = 64% as an orange solid; ^1^H NMR (DMSO-*d*_6_, 400 MHz) δ 7.65 (td, 1H, *J*_1_ = 7.8 Hz, *J*_2_ = 0.9 Hz, Ar-H), 7.55 (d, 1H, *J* = 7.4 Hz, Ar-H), 7.18 (d, 1H, *J* = 8.0 Hz, Ar-H), 7.11 (t, 1H, *J* = 7.5 Hz, Ar-H), 3.69 (q, 2H, *J* = 7.2 Hz, -CH_2_-), 1.17 (t, 3H, *J* = 7.2 Hz, -CH_3_); ^13^C NMR (DMSO-*d*_6_, 100 MHz) δ 183.7 (C = O), 157.8 (C = O), 150.4 (Ar-C), 138.3 (Ar-C), 124.6 (Ar-C), 123.2 (Ar-C), 115.5 (Ar-C), 110.7 (Ar-C), 34.3 (-CH_2_-), 12.3 (-CH_3_); HRMS (ESI) *m/z* calcd for C_10_H_9_NO_2_: 175.0628, found 176.0706 [M + H]^+^.

#### 1-Propylindoline-2,3-dione (4c)

Yield = 53% as an orange solid; ^1^H NMR (DMSO-*d*_6_, 400 MHz) δ 7.65 (td, 1H, *J*_1_ = 7.9 Hz, *J*_2_ = 1.0 Hz, Ar-H), 7.53 (d, 1H, *J* = 7.4 Hz, Ar-H), 7.19 (d, 1H, *J* = 8.0 Hz, Ar-H), 7.11 (t, 1H, *J* = 7.5 Hz, Ar-H), 3.62 (t, 2H, *J* = 7.1 Hz, -CH_2_-), 1.62 (sextet, 2H, *J* = 7.4 Hz, -CH_2_-), 0.90 (t, 3H, *J* = 7.4 Hz, -CH_3_); ^13^C NMR (DMSO-*d*_6_, 100 MHz) δ 183.6 (C = O), 158.1 (C = O), 150.8 (Ar-C), 138.2 (Ar-C), 124.5 (Ar-C), 123.1 (Ar-C), 117.4 (Ar-C), 110.8 (Ar-C), 41.1 (-CH_2_-), 20.2 (-CH_2_-), 11.2 (-CH_3_); HRMS (ESI) *m/z* calcd for C_11_H_11_NO_2_: 189.0784, found 190.0862 [M + H]^+^.

#### 1-Butylindoline-2,3-dione (4d)

Yield = 15% as an orange solid; ^1^H NMR (DMSO-*d*_6_, 400 MHz) δ 7.65 (td, 1H, *J*_1_ = 7.8 Hz, *J*_2_ = 1.0 Hz, Ar-H), 7.54 (d, 1H, *J* = 7.4 Hz, Ar-H), 7.18 (d, 1H, *J* = 8.0 Hz, Ar-H), 7.12 (t, 1H, *J* = 7.5 Hz, Ar-H), 3.65 (t, 2H, *J* = 7.1 Hz, -CH_2_-), 1.58 (br pent, 2H, *J* = 7.1 Hz, -CH_2_-), 1.33 (sextet, 2H, *J* = 7.4 Hz, -CH_2_-), 0.90 (t, 3H, *J* = 7.3 Hz, -CH_3_); ^13^C NMR (DMSO-*d*_6_, 100 MHz) δ 183.6 (C = O), 158.1 (C = O), 150.8 (Ar-C), 138.3 (Ar-C), 124.5 (Ar-C), 123.2 (Ar-C), 117.5 (Ar-C), 110.8 (Ar-C), 39.7 (-CH_2_-, confirmed by DEPT135), 28.9 (-CH_2_-), 19.5 (-CH_2_-), 13.6 (-CH_3_); HRMS (ESI) *m/z* calcd for C_12_H_13_NO_2_: 203.0941, found 204.1019 [M + H]^+^.

#### 1-Pentylindoline-2,3-dione (4e)

Yield = 25% as an orange solid; ^1^H NMR (DMSO-*d*_6_, 400 MHz) δ 7.65 (td, 1H, *J*_1_ = 7.6 Hz, *J*_2_ = 0.8 Hz, Ar-H), 7.54 (d, 1H, *J* = 7.3 Hz, Ar-H), 7.17 (d, 1H, *J* = 8.0 Hz, Ar-H), 7.12 (t, 1H, *J* = 7.5 Hz, Ar-H), 3.64 (t, 2H, *J* = 7.1 Hz, -CH_2_-), 1.59 (br pent, 2H, *J* = 7.0 Hz, -CH_2_-), 1.29 (m, 4H, -(CH_2_)_2_-), 0.85 (t, 3H, *J* = 6.6 Hz, -CH_3_); ^13^C NMR (DMSO-*d*_6_, 100 MHz) δ 183.6 (C = O), 158.1 (C = O), 150.8 (Ar-C), 138.3 (Ar-C), 124.5 (Ar-C), 123.2 (Ar-C), 117.5 (Ar-C), 110.8 (Ar-C), 39.9 (-CH_2_-, confirmed by DEPT135), 28.4 (-CH_2_-), 26.5 (-CH_2_-), 21.9 (-CH_2_-), 13.9 (-CH_3_); HRMS (ESI) *m/z* calcd for C_13_H_15_NO_2_: 217.1097, found 218.1175 [M + H]^+^.

#### 1-Hexylindoline-2,3-dione (4f)

Yield = 9% as an orange solid; ^1^H NMR (DMSO-*d*_6_, 400 MHz) δ 7.65 (td, 1H, *J*_1_ = 7.8 Hz, *J*_2_ = 1.0 Hz, Ar-H), 7.54 (d, 1H, *J* = 7.4 Hz, Ar-H), 7.17 (d, 1H, *J* = 8.0 Hz, Ar-H), 7.12 (t, 1H, *J* = 7.5 Hz, Ar-H), 3.64 (t, 2H, *J* = 7.1 Hz, -CH_2_-), 1.58 (br pent, 2H, *J* = 7.0 Hz, -CH_2_-), 1.29 (m, 6H, -(CH_2_)_3_-), 0.84 (t, 3H, *J* = 6.8 Hz, -CH_3_); ^13^C NMR (DMSO-*d*_6_, 100 MHz) δ 183.6 (C = O), 158.1 (C = O), 150.8 (Ar-C), 138.3 (Ar-C), 124.5 (Ar-C), 123.2 (Ar-C), 117.5 (Ar-C), 110.8 (Ar-C), 39.9 (-CH_2_-, confirmed by DEPT135), 30.9 (-CH_2_-), 26.7 (-CH_2_-), 25.9 (-CH_2_-), 22.1 (-CH_2_-), 13.9 (-CH_3_); HRMS (ESI) *m/z* calcd for C_14_H_17_NO_2_: 231.1254, found 232.1331 [M + H]^+^.

#### 1-Heptylindoline-2,3-dione (4 g)

Yield = 23% as an orange solid; ^1^H NMR (DMSO-*d*_6_, 400 MHz) δ 7.65 (td, 1H, *J*_1_ = 7.8 Hz, *J*_2_ = 0.9 Hz, Ar-H), 7.54 (d, 1H, *J* = 7.4 Hz, Ar-H), 7.17 (d, 1H, *J* = 8.0 Hz, Ar-H), 7.12 (t, 1H, *J* = 7.5 Hz, Ar-H), 3.64 (t, 2H, *J* = 7.1 Hz, -CH_2_-), 1.59 (br pent, 2H, *J* = 6.8 Hz, -CH_2_-), 1.29 (m, 4H, -(CH_2_)_2_-), 1.25 (m, 4H, -(CH_2_)_2_-), 0.86 (t, 3H, *J* = 7.7 Hz, -CH_3_); ^13^C NMR (DMSO-*d*_6_, 100 MHz) δ 183.6 (C = O), 158.1 (C = O), 150.8 (Ar-C), 138.3 (Ar-C), 124.5 (Ar-C), 123.2 (Ar-C), 117.5 (Ar-C), 110.8 (Ar-C), 39.9 (-CH_2_-, confirmed by DEPT135), 31.2 (-CH_2_-), 28.4 (-CH_2_-), 26.8 (-CH_2_-), 26.2 (-CH_2_-), 22.1 (-CH_2_-), 14.0 (-CH_3_); HRMS (ESI) *m/z* calcd for C_15_H_19_NO_2_: 245.1410, found 246.1488 [M + H]^+^.

#### 1-Octylindoline-2,3-dione (4h)

Yield = 10% as an orange solid; ^1^H NMR (DMSO-*d*_6_, 400 MHz) δ 7.65 (t, 1H, *J* = 7.8 Hz, Ar-H), 7.53 (d, 1H, *J* = 7.4 Hz, Ar-H), 7.17 (d, 1H, *J* = 7.9 Hz, Ar-H), 7.12 (t, 1H, *J* = 7.5 Hz, Ar-H), 3.64 (t, 2H, *J* = 7.0 Hz, -CH_2_-), 1.58 (br pent, 2H, *J* = 6.9 Hz, -CH_2_-), 1.28 (m, 4H, -(CH_2_)_2_-), 1.25 (m, 6H, -(CH_2_)_3_-), 0.83 (t, 3H, *J* = 6.9 Hz, -CH_3_); ^13^C NMR (DMSO-*d*_6_, 100 MHz) δ 183.7 (C = O), 158.1 (C = O), 150.8 (Ar-C), 138.3 (Ar-C), 124.6 (Ar-C), 123.2 (Ar-C), 117.5 (Ar-C), 110.8 (Ar-C), 39.9 (-CH_2_-, confirmed by DEPT135), 31.3 (-CH_2_-), 28.71 (-CH_2_-), 28.67 (-CH_2_-), 26.8 (-CH_2_-), 26.3 (-CH_2_-), 22.1 (-CH_2_-), 14.0 (-CH_3_); HRMS (ESI) *m/z* calcd for C_16_H_21_NO_2_: 259.1567, found 260.1646 [M + H]^+^.

#### 1-Nonylindoline-2,3-dione (4i)

Yield = 40% as an orange solid; ^1^H NMR (DMSO-*d*_6_, 400 MHz) δ 7.64 (td, 1H, *J*_1_ = 7.5 Hz, *J*_2_ = 0.7 Hz Ar-H), 7.53 (d, 1H, *J* = 7.1 Hz, Ar-H), 7.17 (d, 1H, *J* = 7.9 Hz, Ar-H), 7.11 (t, 1H, *J* = 7.5 Hz, Ar-H), 3.64 (t, 2H, *J* = 7.1 Hz, -CH_2_-), 1.57 (br pent, 2H, *J* = 6.9 Hz, -CH_2_-), 1.27 (m, 4H, -(CH_2_)_2_-), 1.21 (m, 8H, -(CH_2_)_4_-), 0.83 (t, 3H, *J* = 6.4 Hz, -CH_3_); ^13^C NMR (DMSO-*d*_6_, 100 MHz) δ 183.6 (C = O), 158.1 (C = O), 150.8 (Ar-C), 138.3 (Ar-C), 124.6 (Ar-C), 123.2 (Ar-C), 117.5 (Ar-C), 110.8 (Ar-C), 39.9 (-CH_2_-, confirmed by DEPT135), 31.3 (-CH_2_-), 28.9 (-CH_2_-), 28.74 (-CH_2_-), 28.69 (-CH_2_-), 26.8 (-CH_2_-), 26.3 (-CH_2_-), 22.1 (-CH_2_-), 14.0 (-CH_3_); HRMS (ESI) *m/z* calcd for C_17_H_23_NO_2_: 273.1723, found 274.1802 [M + H]^+^.

#### 1-Decylindoline-2,3-dione (4j)

Yield = 52% as an orange solid; ^1^H NMR (DMSO-*d*_6_, 400 MHz) δ 7.64 (t, 1H, *J* = 7.8 Hz, Ar-H), 7.53 (d, 1H, *J* = 7.4 Hz, Ar-H), 7.17 (d, 1H, *J* = 8.0 Hz, Ar-H), 7.11 (t, 1H, *J* = 6.4 Hz, Ar-H), 3.63 (t, 2H, *J* = 7.0 Hz, -CH_2_-), 1.57 (br pent, 2H, *J* = 6.7 Hz, -CH_2_-), 1.27 (m, 4H, -(CH_2_)_2_-), 1.20 (m, 10H, -(CH_2_)_5_-), 0.82 (t, 3H, *J* = 6.4 Hz, -CH_3_); ^13^C NMR (DMSO-*d*_6_, 100 MHz) δ 183.6 (C = O), 158.1 (C = O), 150.8 (Ar-C), 138.3 (Ar-C), 124.5 (Ar-C), 123.2 (Ar-C), 117.5 (Ar-C), 110.8 (Ar-C), 39.9 (-CH_2_-, confirmed by DEPT135), 31.3 (-CH_2_-), 29.0 (-(CH_2_)_2_-), 28.7 (-(CH_2_)_2_-), 26.8 (-CH_2_-), 26.3 (-CH_2_-), 22.1 (-CH_2_-), 14.0 (-CH_3_); HRMS (ESI) *m/z* calcd for C_18_H_25_NO_2_: 287.1880, found 288.1958 [M + H]^+^.

### General procedure for synthesis of N-alkyl indoles (5a-j)

Indole (200 mg, 1.71 mmol), iodomethane (1 eq) or 1-bromoalkane (1 eq), NaOH (2 eq), and DMSO (3 ml) were combined and stirred at rt for 24 h. The reaction mixture was then diluted with water (100 ml) and extracted with EtOAc (3 × 15 ml). The combined organic layers were washed with brine (50 ml), dried (MgSO_4_), and concentrated under reduced pressure to afford the crude product, which was then purified by flash column chromatography (SiO_2_; hexanes to 10:1/hexanes:EtOAc) to yield the desired product.

#### 1-Methyl-1H-indole (5a)

Yield = 50% as pale yellow oil; ^1^H NMR (DMSO-*d*_6_, 400 MHz) δ 7.54 (d, 1H, *J* = 7.9 Hz, Ar-H), 7.42 (d, 1H, *J* = 8.2 Hz, Ar-H), 7.31 (d, 1H, *J* = 3.0 Hz, Ar-H), 7.14 (td, 1H, *J*_1_ = 7.3 Hz, *J*_2_ = 0.6, Ar-H), 7.02 (td, 1H, *J*_1_ = 7.7 Hz, *J*_2_ = 0.6, Ar-H), 6.41 (d, 1H, *J* = 2.4 Hz, Ar-H), 3.78 (s, 3H, -CH_3_); ^13^C NMR (DMSO-*d*_6_, 100 MHz) δ 136.3 (Ar-C), 129.5 (Ar-C), 128.0 (Ar-C), 121.0 (Ar-C), 120.3 (Ar-C), 118.9 (Ar-C), 109.6 (Ar-C), 100.2 (Ar-C), 32.4 (-CH_3_); HRMS (ESI) *m/z* calcd for C_9_H_9_N: 131.0730, found 132.0808 [M + H]^+^.

#### 1-Ethyl-1H-indole (5b)

Yield = 13% as a pale yellow oil; ^1^H NMR (DMSO-*d*_6_, 400 MHz) δ 7.53 (d, 1H, *J* = 7.8 Hz, Ar-H), 7.46 (d, 1H, *J* = 8.2 Hz, Ar-H), 7.37 (d, 1H, *J* = 3.1 Hz, Ar-H), 7.12 (t, 1H, *J* = 7.9 Hz, Ar-H), 7.00 (t, 1H, *J* = 7.8 Hz, Ar-H), 6.41 (d, 1H, *J* = 2.6 Hz, Ar-H), 4.20 (q, 2H, *J* = 7.0 Hz, -CH_2_-), 1.35 (t, 3H, *J* = 7.2 Hz, -CH_3_-); ^13^C NMR (DMSO-*d*_6_, 100 MHz) δ 135.3 (Ar-C), 128.1 (Ar-C), 127.9 (Ar-C), 120.9 (Ar-C), 120.4 (Ar-C), 118.8 (Ar-C), 109.6 (Ar-C), 100.4 (Ar-C), 40.2 (-CH_2_-), 15.5 (-CH_3_); HRMS (ESI) *m/z* calcd for C_10_H_11_N: 145.0886, found 146.0963 [M + H]^+^.

#### 1-Propyl-1H-indole (5c)

Yield = 20% as a pale yellow oil; ^1^H NMR (DMSO-*d*_6_, 400 MHz) δ 7.53 (d, 1H, *J* = 7.8 Hz, Ar-H), 7.45 (d, 1H, *J* = 8.2 Hz, Ar-H), 7.34 (d, 1H, *J* = 3.0 Hz, Ar-H), 7.11 (t, 1H, *J* = 7.9 Hz, Ar-H), 7.00 (t, 1H, *J* = 7.1 Hz, Ar-H), 6.41 (d, 1H, *J* = 2.6 Hz, Ar-H), 4.12 (t, 2H, *J* = 7.0 Hz, -CH_2_-), 1.75 (sextet, 2H, *J* = 7.3 Hz, -CH_2_-), 0.82 (t, 3H, *J* = 7.4 Hz, -CH_3_); ^13^C NMR (DMSO-*d*_6_, 100 MHz) δ 135.7 (Ar-C), 128.7 (Ar-C), 128.1 (Ar-C), 120.9 (Ar-C), 120.4 (Ar-C), 118.8 (Ar-C), 109.8 (Ar-C), 100.3 (Ar-C), 47.1 (-CH_2_-), 23.2 (-CH_2_-), 11.2 (-CH_3_); HRMS (ESI) *m/z* calcd for C_11_H_13_N: 159.1043, found 160.1120 [M + H]^+^.

#### 1-Butyl-1H-indole (5d)

Yield = 58% as a pale yellow oil; ^1^H NMR (DMSO-*d*_6_, 400 MHz) δ 7.54 (d, 1H, *J* = 7.8 Hz, Ar-H), 7.44 (d, 1H, *J* = 8.2 Hz, Ar-H), 7.35 (d, 1H, *J* = 3.1 Hz, Ar-H), 7.12 (td, 1H, *J*_1_ = 7.3 Hz, *J*_2_ = 0.5, Ar-H), 7.01 (td, 1H, *J*_1_ = 7.1 Hz, *J*_2_ = 0.6 Ar-H), 6.41 (d, 1H, *J* = 2.6 Hz, Ar-H), 4.14 (t, 2H, *J* = 7.0 Hz, -CH_2_-), 1.71 (pent, 2H, *J* = 7.1 Hz, -CH_2_-), 1.22 (sextet, 2H, *J* = 7.5 Hz, -CH_2_-) 0.87 (t, 3H, *J* = 7.4 Hz, -CH_3_); ^13^C NMR (DMSO-*d*_6_, 100 MHz) δ 135.7 (Ar-C), 128.6 (Ar-C), 128.1 (Ar-C), 120.9 (Ar-C), 120.4 (Ar-C), 118.8 (Ar-C), 109.7 (Ar-C), 100.3 (Ar-C), 45.2 (-CH_2_-), 32.0 (-CH_2_-), 19.5 (-CH_2_-), 13.6 (-CH_3_); HRMS (ESI) *m/z* calcd for C_12_H_15_N: 173.1199, found 174.1277 [M + H]^+^.

#### 1-Pentyl-1H-indole (5e)

Yield = 17% as a pale yellow oil; ^1^H NMR (DMSO-*d*_6_, 400 MHz) δ 7.53 (d, 1H, *J* = 7.8 Hz, Ar-H), 7.44 (d, 1H, *J* = 8.2 Hz, Ar-H), 7.34 (d, 1H, *J* = 3.1 Hz, Ar-H), 7.11 (t, 1H, *J* = 7.8 Hz, Ar-H), 7.00 (t, 1H, *J* = 7.7 Hz, Ar-H), 6.41 (d, 1H, *J* = 2.6 Hz, Ar-H), 4.15 (t, 2H, *J* = 7.0 Hz, -CH_2_-), 1.74 (pent, 2H, *J* = 7.2 Hz, -CH_2_-), 1.30 (sextet, 2H, *J* = 6.1 Hz, -CH_2_-) 1.20 (m, 2H, *J* = 7.4 Hz, -CH_2_-) 0.83 (t, 3H, *J* = 7.0 Hz, -CH_3_); ^13^C NMR (DMSO-*d*_6_, 100 MHz) δ 135.6 (Ar-C), 128.6 (Ar-C), 128.1 (Ar-C), 120.9 (Ar-C), 120.4 (Ar-C), 118.8 (Ar-C), 109.7 (Ar-C), 100.3 (Ar-C), 45.4 (-CH_2_-), 29.6 (-CH_2_-), 28.5 (-CH_2_-), 21.8 (-CH_2_-), 13.9 (-CH_3_); HRMS (ESI) *m/z* calcd for C_13_H_17_N: 187.1356, found 188.1433 [M + H]^+^.

#### 1-Hexyl-1H-indole (5f)

Yield = 26% as a pale yellow oil; ^1^H NMR (DMSO-*d*_6_, 400 MHz) δ 7.53 (d, 1H, *J* = 7.8 Hz, Ar-H), 7.44 (d, 1H, *J* = 8.2 Hz, Ar-H), 7.35 (d, 1H, *J* = 3.0 Hz, Ar-H), 7.11 (t, 1H, *J* = 7.7 Hz, Ar-H), 7.00 (t, 1H, *J* = 7.6 Hz, Ar-H), 6.40 (d, 1H, *J* = 2.7 Hz, Ar-H), 4.15 (t, 2H, *J* = 7.0 Hz, -CH_2_-), 1.74 (m, 2H, -CH_2_-), 1.24 (br m, 6H, -(CH_2_)_3_-), 0.82 (m, 3H, -CH_3_); ^13^C NMR (DMSO-*d*_6_, 100 MHz) δ 135.6 (Ar-C), 128.6 (Ar-C), 128.1 (Ar-C), 120.9 (Ar-C), 120.4 (Ar-C), 118.7 (Ar-C), 109.7 (Ar-C), 100.3 (Ar-C), 45.4 (-CH_2_-), 30.8 (-CH_2_-), 29.8 (-CH_2_-), 25.9 (-CH_2_-), 22.0 (-CH_2_-), 13.9 (-CH_3_); HRMS (ESI) *m/z* calcd for C_14_H_19_N: 201.1512, found 202.1589 [M + H]^+^.

#### 1-Heptyl-1H-indole (5 g)

Yield = 69% as a pale yellow oil; ^1^H NMR (DMSO-*d*_6_, 400 MHz) δ 7.53 (d, 1H, *J* = 7.8 Hz, Ar-H), 7.44 (d, 1H, *J* = 8.2 Hz, Ar-H), 7.34 (d, 1H, *J* = 3.1 Hz, Ar-H), 7.11 (t, 1H, *J* = 7.8 Hz, Ar-H), 6.99 (t, 1H, *J* = 7.6 Hz, Ar-H), 6.40 (d, 1H, *J* = 2.6 Hz, Ar-H), 4.15 (t, 2H, *J* = 7.0 Hz, -CH_2_-), 1.73 (m, 2H, -CH_2_-), 1.21 (br m, 8H, -(CH_2_)_4_-), 0.83 (m, 3H, -CH_3_); ^13^C NMR (DMSO-*d*_6_, 100 MHz) δ 135.6 (Ar-C), 128.6 (Ar-C), 128.1 (Ar-C), 120.9 (Ar-C), 120.4 (Ar-C), 118.8 (Ar-C), 109.7 (Ar-C), 100.3 (Ar-C), 45.4 (-CH_2_-), 31.2 (-CH_2_-), 29.9 (-CH_2_-), 28.3 (-CH_2_-), 26.2 (-CH_2_-), 22.0 (-CH_2_-), 13.9 (-CH_3_); HRMS (ESI) *m/z* calcd for C_15_H_21_N: 215.1669, found 216.1747 [M + H]^+^.

#### 1-Octyl-1H-indole (5h)

Yield = 26% as a pale yellow oil; ^1^H NMR (DMSO-*d*_6_, 400 MHz) δ 7.53 (d, 1H, *J* = 7.8 Hz, Ar-H), 7.44 (d, 1H, *J* = 8.2 Hz, Ar-H), 7.35 (d, 1H, *J* = 3.1 Hz, Ar-H), 7.11 (t, 1H, *J* = 7.3 Hz, Ar-H), 7.00 (t, 1H, *J* = 7.3 Hz, Ar-H), 6.40 (d, 1H, *J* = 2.8 Hz, Ar-H), 4.15 (t, 2H, *J* = 7.0 Hz, -CH_2_-), 1.74 (br pent, 2H, *J* = 7.2 Hz, -CH_2_-), 1.22 (br m, 10H, -(CH_2_)_5_-), 0.83 (t, 3H, *J* = 6.5 Hz, -CH_3_); ^13^C NMR (DMSO-*d*_6_, 100 MHz) δ 135.6 (Ar-C), 128.6 (Ar-C), 128.1 (Ar-C), 120.9 (Ar-C), 120.4 (Ar-C), 118.8 (Ar-C), 109.7 (Ar-C), 100.3 (Ar-C), 45.4 (-CH_2_-), 31.2 (-CH_2_-), 29.8 (-CH_2_-), 28.64 (-CH_2_-), 28.59 (-CH_2_-), 26.3 (-CH_2_-), 22.1 (-CH_2_-), 13.9 (-CH_3_); HRMS (ESI) *m/z* calcd for C_16_H_23_N: 229.1825, found 230.1904 [M + H]^+^.

#### 1-Nonyl-1H-indole (5i)

Yield = 70% as a pale yellow oil; ^1^H NMR (DMSO-*d*_6_, 400 MHz) δ 7.53 (d, 1H, *J* = 7.8 Hz, Ar-H), 7.44 (d, 1H, *J* = 8.2 Hz, Ar-H), 7.34 (d, 1H, *J* = 3.0 Hz, Ar-H), 7.11 (t, 1H, *J* = 7.7 Hz, Ar-H), 6.99 (t, 1H, *J* = 7.6 Hz, Ar-H), 6.40 (d, 1H, *J* = 2.6 Hz, Ar-H), 4.14 (t, 2H, *J* = 7.0 Hz, -CH_2_-), 1.72 (br pent, 2H, *J* = 7.2 Hz, -CH_2_-), 1.20 (br m, 12H, -(CH_2_)_6_-), 0.83 (t, 3H, *J* = 6.5 Hz, -CH_3_); ^13^C NMR (DMSO-*d*_6_, 100 MHz) δ 135.6 (Ar-C), 128.6 (Ar-C), 128.1 (Ar-C), 120.8 (Ar-C), 120.4 (Ar-C), 118.7 (Ar-C), 109.7 (Ar-C), 100.3 (Ar-C), 45.4 (-CH_2_-), 31.2 (-CH_2_-), 29.8 (-CH_2_-), 28.9 (-CH_2_-), 28.63 (-CH_2_-), 28.60 (-CH_2_-), 26.3 (-CH_2_-), 22.1 (-CH_2_-), 13.9 (-CH_3_); HRMS (ESI) *m/z* calcd for C_17_H_25_N: 243.1982, found 244.2061 [M + H]^+^.

#### 1-Decyl-1H-indole (5j)

Yield = 76% as a green oil; ^1^H NMR (DMSO-*d*_6_, 400 MHz) δ 7.53 (d, 1H, *J* = 7.8 Hz, Ar-H), 7.44 (d, 1H, *J* = 8.2 Hz, Ar-H), 7.34 (d, 1H, *J* = 3.0 Hz, Ar-H), 7.11 (t, 1H, *J* = 7.4 Hz, Ar-H), 6.99 (t, 1H, *J* = 7.6 Hz, Ar-H), 6.40 (d, 1H, *J* = 2.7 Hz, Ar-H), 4.14 (t, 2H, *J* = 7.0 Hz, -CH_2_-), 1.73 (br pent, 2H, *J* = 7.1 Hz, -CH_2_-), 1.20 (br m, 14H, -(CH_2_)_7_-), 0.84 (t, 3H, *J* = 6.5 Hz, -CH_3_); ^13^C NMR (DMSO-*d*_6_, 100 MHz) δ 135.6 (Ar-C), 128.5 (Ar-C), 128.1 (Ar-C), 120.8 (Ar-C), 120.4 (Ar-C), 118.7 (Ar-C), 109.7 (Ar-C), 100.3 (Ar-C), 45.4 (-CH_2_-), 31.2 (-CH_2_-), 29.8 (-CH_2_-), 29.0 (-CH_2_-), 28.9 (-CH_2_-), 28.7 (-CH_2_-), 28.6 (-CH_2_-), 26.3 (-CH_2_-), 22.1 (-CH_2_-), 14.0 (-CH_3_); HRMS (ESI) *m/z* calcd for C_18_H_27_N: 257.2138, found 258.2220 [M + H]^+^.

### AChE and BChE inhibition and kinetics assays

Inhibition of AChE (Sigma-Aldrich, Product No. C2888, from *Electrophorus electricus*) and BChE (Lee Biosolutions, Product No. 130–10-1, from equine serum) was carried out as previously described by our lab^18^^,^^19^. Briefly, compounds to be tested were prepared in sodium phosphate (dibasic) buffer (0.1 M) containing 10% DMSO and then diluted with phosphate buffer across the wells of a 96-well plate to reach desired concentrations (50 µL total well volume). AChE (50 µL, 400 U/L, in phosphate buffer) or BChE (50 µL, 400 U/L, in phosphate buffer) was added to the appropriate wells. After incubation, the enzymatic reaction was initiated with 100 µL of a 1:1 (v/v) solution of acetylthiocholine (ATC, 2 mM in phosphate buffer) or butyrylthiocholine (BTC, 2 mM in phosphate buffer) and 5,5′-dithiobis-(2-nitrobenzoic acid) (DTNB, 1 mM in phosphate buffer). The change in absorbance at 405 nm was monitored over 10 min and used to graph percent inhibition vs concentration of inhibitor (log transformed). IC_50_ values were calculated using GraphPad Prism 9.1.1.

Kinetics assays of **4i** inhibition with AChE and BChE were also carried out as previously described^18^^,^^19^. Briefly, varying concentrations of **4i** were incubated with AChE and BChE before initiating the enzymatic reactions by addition of 100 µL of a 1:1 (v/v) solution of ATC or BTC (0.4 mM, 0.5 mM, 1 mM, or 2 mM in phosphate buffer) and DTNB (1 mM in phosphate buffer) to all wells. The change in absorbance at 405 nm over 10 min was used to calculate the rate of reaction (mean ΔAbs/min ± SEM for three replicates). Rate vs concentration was fitted to a Lineweaver-Burk plot using GraphPad Prism 9.1.1.

### Molecular modelling

Molecular modelling was run using the modelling program AutoDock Vina (http://vina.scripps.edu/) as previously described by our lab^18^^,^^19^. Briefly, the crystal structures of *Tc*AChE (PDB ID: 1ACJ, 2.8 Å resolution) and human BChE (PDB ID: 4BDS, 2.1 Å resolution) were edited in PyMOL and AutoDock Tools. Ligands **4i** and **5i** were prepared using ChemDraw and Chem3D, as well as AutoDock Tools. The scoring grid (36 × 36 × 36 Å) for AutoDock Vina was centred as follows: AChE – x-center: 4.766, y-center: 65.514, z-center: 56.822; BChE – x-center: 138.790, y-center: 123.636, z-center: 38.646. PyMOL was used for results visualisation.

## Results and discussion

The synthesis of the target molecules **4a**-**j** and **5a**-**j** was carried out in straightforward fashion by substitution of isatin or indole with iodomethane or 1-bromoalkanes in the presence of K_2_CO_3_ and DMF or NaOH and DMSO (Scheme 1). Yields ranged from 9–76%, and structures were confirmed by ^1^H NMR,^13^C NMR, and HRMS. In ^13^C NMR (DMSO-*d_6_*, 400 MHz) spectra for **4d-j**, the residual DMSO signal overlapped with the signal from the methylene carbon adjacent to the nitrogen. A DEPT135 experiment was run to differentiate this signal (negative) from the solvent (positive) (see supplementary data), as we have employed previosuly^18^. The synthesised compounds were then evaluated as AChEI and BChEI using the established Ellman method^20^. Results are reported as IC_50_ values in Table 1.

**Table 1.** Inhibition of AChE and BChE by *N*-alkyl isatins **4a**-**j** and indoles **5a**-**j**.

Compound	*n*	AChE IC_50_ (µM)^a^	BChE IC_50_ (µM)^a^	Selectivity^b^	BBB permeability^c^
**4a**	0	647 ± 57	> 1000	< 0.6	1.16 (moderate)
**4b**	1	297 ± 35	193 ± 8	1.5	1.48 (moderate)
**4c**	2	519 ± 11	257 ± 8	2.0	2.00 (moderate)
**4d**	3	540 ± 23^c^	73.7 ± 1.3	7.3	2.38 (high)
**4e**	4	197 ± 51	83.8 ± 3.4	2.4	2.55 (high)
**4f**	5	> 125	90.8 ± 2.3	> 1.4	0.25 (moderate)
**4g**	6	67.8 ± 7.8	20.7 ± 3.3	3.3	0.39 (moderate)
**4h**	7	> 125	17.9 ± 1.8	> 7.0	0.54 (moderate)
**4i**	8	82.1 ± 8.1	3.77 ± 0.45	21.8	0.49 (moderate)
**4j**	9	62.2 ± 8.5	5.16 ± 0.66	12.1	0.39 (moderate)
**5a**	0	86.7 ± 4.5	> 125	< 0.7	1.65 (moderate)
**5b**	1	101 ± 6	> 125	< 0.8	1.67 (moderate)
**5c**	2	78.6 ± 2.3	> 125	< 0.6	1.96 (moderate)
**5d**	3	> 125	> 125	–	2.56 (high)
**5e**	4	95.0 ± 5.9	> 125	< 0.8	3.58 (high)
**5f**	5	76.2 ± 1.6	> 125	< 0.6	6.60 (high)
**5g**	6	35.0 ± 10.5	> 62.5	< 0.6	9.04 (high)
**5h**	7	49.5 ± 1.8	> 62.5	< 0.8	11.00 (high)
**5i**	8	44.6 ± 1.7	> 62.5	< 0.7	13.01 (high)
**5j**	9	48.6 ± 1.4	> 62.5	< 0.8	19.26 (high)
**isatin**	–	> 1000^d^	> 1000^d^	–	0.67 (moderate)
**rivastigmine**	–	275 ± 25^d^	1.14 ± 0.13^d^	241	1.16 (moderate)
**tacrine**	–	0.00187 ± 0.00002^d^	0.0128 ± 0.0012^d^	0.14	0.87 (moderate)

^a^ IC_50_ values are reported as the mean of three independent experiments ± SEM. AChE was from *Electrophorus electricus*. BChE was from equine serum. For compounds with limited solubility under the assay conditions, the IC_50_ is given as > X, where X = the highest soluble concentration tested.

^b^ Selectivity was calculated as AChE IC_50_/BChE IC_50_.

^c^ Predicted using the PreADMET application (https://preadmet.qsarhub.com/.) where low BBB permeability < 0.1, moderate BBB permeability 0.1–2.0, high BBB permeability > 2.0.

^d^ Values previously determined^18^^,^^19^.

The best AChEI from each series was **4j** (*n* = 9, IC_50_ = 62.2 µM) and **5 g** (*n* = 6, IC_50_ = 35.0 µM). Regarding AChE IC_50_, there is no well-defined SAR for alkyl chain length in either series. *N*-Alkyl isatins **4a**-**j** show a loose trend in that longer alkyl chains (**4 g**, **4i**, **4j**) show better inhibition of AChE than shorter chains (**4a**-**e**), but the *N*-alkyl indoles **5a**-**j** show little difference between the shortest alkyl chain (**5a**) and the longest (**5j**). The best BChEI was **4i** (*n* = 8, IC_50_ = 3.77 µM). *N*-Alkyl isatins **4a**-**j** showed a SAR that as the alkyl chain gets longer, the BChE IC_50_ decreases. Interestingly, none of the *N*-alkyl indoles **5a**-**j** showed significant inhibition of BChE at the concentrations tested. Thus, the indole series selectively inhibited AChE while the isatin series selectively inhibited BChE.

To put these IC_50_ values in context, they were compared to isatin and two well-known inhibitors, rivastigmine and tacrine, which we have previously used for comparison (Table 1)^18^^,^^19^. In most cases, the addition of an alkyl group to isatin significantly improves activity over the parent compound for both enzymes. The best AChEI (**5 g**) and BChEI (**4i**) from the compounds under investigation were comparable in activity to rivastigmine, a clinically used inhibitor for AD, against both AChE and BChE. However, neither **5g** or **4i** was as active against AChE or BChE as tacrine, an inhibitor extensively studied in the literature as an AChEI and BChEI and formerly used clinically.

As mentioned earlier, there was, to the best of our knowledge, no comprehensive report of *N*-alkyl isatins or indoles addressing the effect of alkyl chain length on inhibition of these enzymes. However, **4a** was previously reported by Hyatt *et al.* to have a *K*_i_ > 100 µM for both AChE and BChE^21^. This same group also reported that the *N*-dodecyl and *N*-hexadecyl isatins have *K*_i_ values > 100 µM for both AChE and BChE. However, these were the only three *N*-alkyl isatins tested. Interestingly, and more relevant to the discussion of the current study, Hyatt *et al.* reached the conclusion that the inhibitory potency of isatin compounds against carboxylesterases was related to their hydrophobicity, with analogs showing a clogP > 5 commonly having nM range *K*_i_ values. This trend and conclusion certainly apply to the current study, particularly among **4a**-**j** regarding BChE inhibitory potency. Our observations are also like a previous report of *N*-alkyl tacrines, in which longer alky chains improved inhibition of AChE, and, in all cases except one, the *N*-alkyl tacrines showed better AChE inhibition than the parent tacrine molecule^22^.

**Figure 2.**
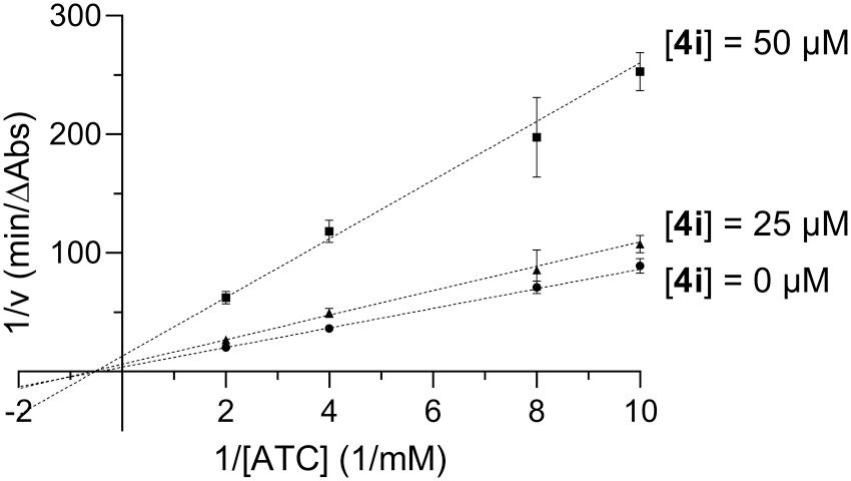
Lineweaver-Burk plot showing non-competitive inhibition of AChE with respect to acetylthiocholine (ATC) for compound **4i**.

**Figure 3.**
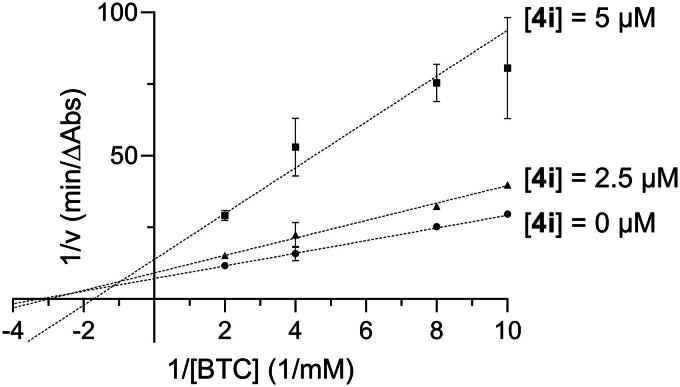
Lineweaver-Burk plot showing mixed inhibition of BChE with respect to butyrylthiocholine (BTC) for compound **4i**.

**Figure 4.**
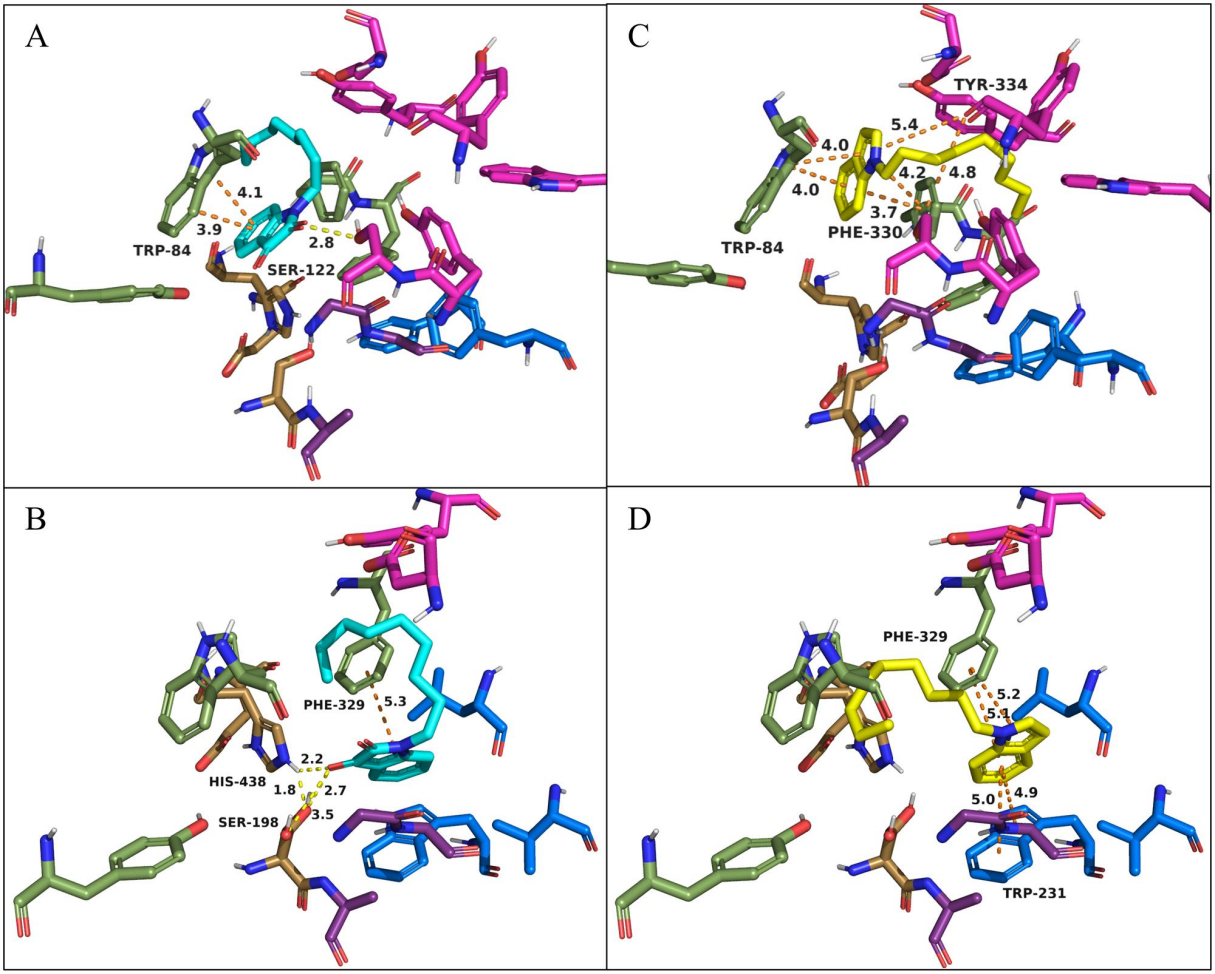
Docking of *Tc*AChE (PDB: 1ACJ; **A** and **C**) and hBChE (PDB: 4BDS; **B** and **D**) with 1-nonylindoline-2,3-dione (**4i**, cyan) and 1-nonyl-1*H*-indole (**5i**, yellow). Active site residues of *Tc*AChE include the catalytic triad (Ser200, His440, Glu327; gold), anionic site (Trp84, Tyr130, Phe330, and Phe331; green), oxyanion hole (Gly118, Gly119, Ala201; purple), acyl pocket (Phe288, Phe290; blue), and PAS (Tyr70, Asp72, Tyr121, Ser122, Trp279, Tyr334; magenta). Active site residues of hBChE include the catalytic triad (Ser198, His438, Glu325; gold), anionic site (Trp82, Tyr128, Phe329; green), oxyanion hole (Gly116, Gly117, Ala199; purple), acyl pocket (Trp231, Leu286, Val288; blue), and PAS (Asp70, Tyr332; magenta). Orange dashes indicate π-π stacking interactions, and yellow dashes indicate H-bonding interactions. All distances shown are in Å. O, N, and H atoms are shown in red, blue, and white, respectively.

Compound **4i** was chosen as a representative compound to investigate mode of inhibition through Lineweaver-Burk analysis. As shown in Figures 2 and 3, **4i** displayed a non-competitive inhibition pattern with AChE and a mixed inhibition pattern with BChE. Previously we reported similar observations for isatin- and indole-based inhibitors. Namely, **3** showed non-competitive inhibition with AChE, while **1** and **2** showed mixed inhibition with BChE^18^^,^^19^. Thus, the current results are in agreement with these previous findings.

To better understand the results of the enzyme inhibition studies, molecular modelling of **4i** and **5i** with *Tc*AChE (PDB: 1ACJ) and hBChE (PDB: 4BDS) was performed using AutoDock Vina by a method previously reported^18^^,^^19^^,^^23–25^. Results are shown in Figure 4(A–D). Active site residues of *Tc*AChE include the catalytic triad (Ser200, His440, Glu327; gold), anionic site (Trp84, Tyr130, Phe330, and Phe331; green), oxyanion hole (Gly118, Gly119, Ala201; purple), acyl pocket (Phe288, Phe290; blue), and PAS (Tyr70, Asp72, Tyr121, Ser122, Trp279, Tyr334; magenta). Active site residues of hBChE include the catalytic triad (Ser198, His438, Glu325; gold), anionic site (Trp82, Tyr128, Phe329; green), oxyanion hole (Gly116, Gly117, Ala199; purple), acyl pocket (Trp231, Leu286, Val288; blue), and PAS (Asp70, Tyr332; magenta).

**4i** was predicted to bind to the active site of *Tc*AChE with a parallel π-π stacking between the isatin moiety and Trp84, as well as a H-bonding interaction between the 2-oxo group and side chain OH of Ser122 (Figure 4(A)). In comparison, **4i** was predicted to bind to the active site of hBChE with a perpendicular π-π stacking between the isatin moiety and Phe329, and a H-bonding interaction was also indicated between the 3-oxo group and side chain OH and NH of Ser198 and His438, respectively (Figure 4(B)). This direct H-bonding interaction between **4i** and Ser198 and His438, which are part of the catalytic triad, may account for the enhanced potency seen against BChE (22-fold selectivity). Parallel π-π stacking with Trp84 and Phe330 and perpendicular π-π stacking with Tyr334 were predicted for the indole moiety of **5i** when binding to the active site of *Tc*AChE (Figure 4(C)). When binding to hBChE, the indole moiety of **5i** was predicted to form perpendicular π-π stacking with Trp231 and Phe329 (Figure 4(D)). In all cases, the alkyl chain of the inhibitors has no binding interactions with the enzymes (expected due to lack of functionality). The alkyl chain may enhance binding to the enzyme active site by a hydrophobic effect or a steric directing effect or a combination of the two. This likely accounts for the observations that longer alkyl chains increase inhibition.

The shift from the isatin moiety to the indole moiety (**4i** vs. **5i**) results in the loss of the 2-oxo and 3-oxo groups that participate in H-bonding interactions between **4i** and the active sites of both enzymes. In the case of *Tc*AChE, it appears that the lost H-bond with Ser122 is compensated for by the gain of π-π interactions with Phe330 and Tyr334 (Figure 4(A) vs. 4(C)), which may explain the similar potency of **4i** and **5i** towards AChE in the inhibition assay. However, in the case of hBChE, it appears that the lost H-bonds with Ser198 and His438 cannot be compensated for by the gain of a π-π interaction with Trp231 (Figure 4(B) vs. 4(D)). This likely accounts for the significant loss of potency (>17-fold) towards BChE for **5i** compared to **4i** in the inhibition assay.

Lastly, recognising that targeting AChE and BChE in the context of AD means that compounds in question must be able to cross the blood-brain barrier (BBB), a BBB permeability was predicted using the PreADMET application (https://preadmet.qsarhub.com/). Results are shown in Table 1 where compounds were assigned a low, moderate, or high BBB permeability. Of note, all compounds in this study (**4a**-**j** and **5a**-**j**) were predicted to have moderate or high BBB permeability.

## Conclusion

Overall, the current study demonstrates that the addition of an *N*-alkyl chain to isatin increases inhibition of both AChE and BChE. For BChE, there is a clear trend indicating that the inhibitory potency increases as the length of the alkyl chain increases. Shifting to an indole scaffold resulted in a loss of BChE potency, but AChE potency was maintained. This observation, supported by molecular modelling, indicates a key role for the 3-oxo group of isatin in BChE inhibition. Results do not discount the strategy of linking isatin to another moiety by an alkyl linker, as in **1**–**3**, as this additional moiety may offer other properties to make these compounds relevant to AD (e.g. metal binding, reactive oxygen species inhibition). However, results do show that adding a simple alkyl chain can lead to significantly improved inhibition of AChE and BChE.

## Supplementary Material

Supplemental Material

## Data Availability

The data that support the findings of this study are available from the corresponding author, TJE, upon reasonable request.
