# Supplementary material for: Evaluation of N-alkyl isatins and indoles as acetylcholinesterase and butyrylcholinesterase inhibitors
Source: J Enzyme Inhib Med Chem. 2023 Dec 7;39(1):2286935. doi: 10.1080/14756366.2023.2286935 (PMC11721616; doi:10.1080/14756366.2023.2286935)
Supplement: Supplemental Material [file IENZ_A_2286935_SM6087.pdf]

Journal of Enzyme Inhibition and Medicinal Chemistry
Regular Article – Supplementary Material

Evaluation of *N*-Alkyl Isatins and Indoles as Acetylcholinesterase and Butyrylcholinesterase Inhibitors

Kaitlyn N. Alcorn,[a] Isabelle A. Oberhauser,[a] Matthew D. Politeski,[a] and Todd J. Eckroat[a]*

[a]*School of Science, Penn State Erie, The Behrend College, Erie, PA 16563, USA*

**To whom correspondence should be addressed. e-mail: tje146@psu.edu, phone: 1-814-898-6839*

Supplementary Material Contents

1. Copies of ^{1}H NMR, ^{13}C NMR, and HRMS spectra for *N*-alkyl isatins **4a-j**
2. Copies of ^{1}H NMR, ^{13}C NMR, and HRMS spectra for *N*-alkyl indoles **5a-j**
3. List of abbreviations

1. Copies of ^{1}H NMR, ^{13}C NMR, and HRMS spectra for *N*-alkyl isatins 4a-j

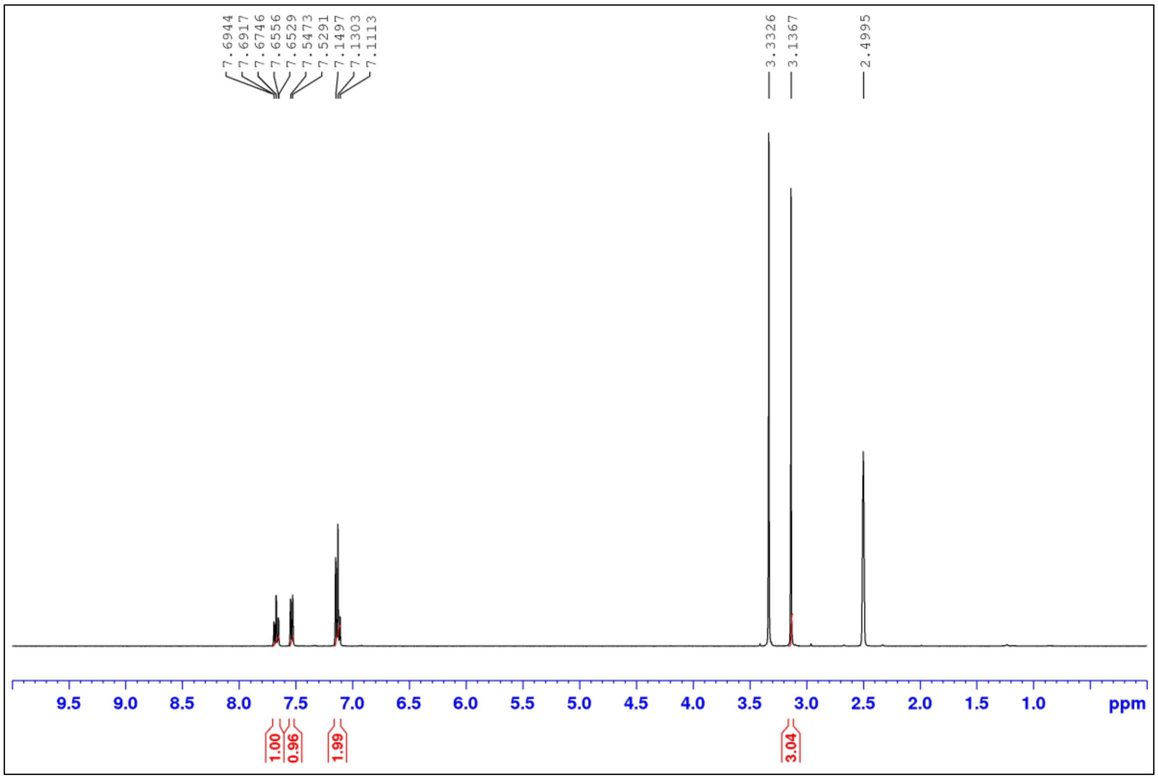

Fig. S1 ^{1}H NMR (DMSO-d_6, 400 MHz) spectrum of 1-methylindoline-2,3-dione (**4a**)

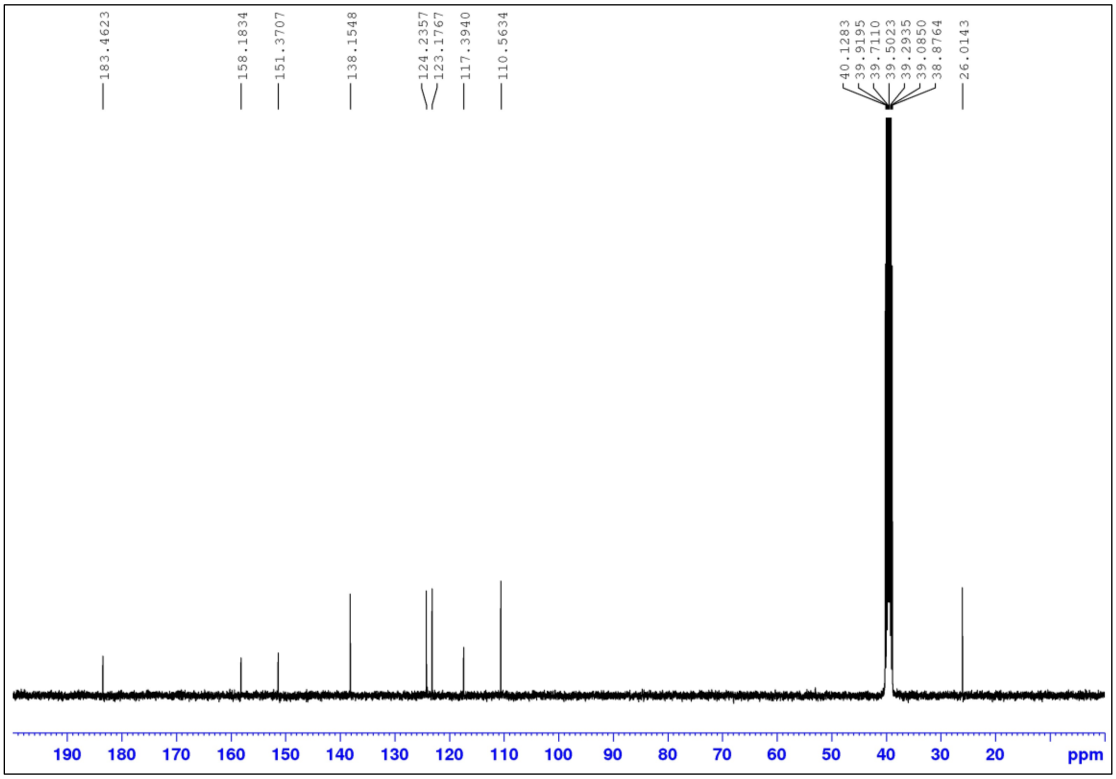

Fig. S2 ^{13}C NMR (DMSO-d_6, 100 MHz) spectrum of 1-methylindoline-2,3-dione (**4a**)

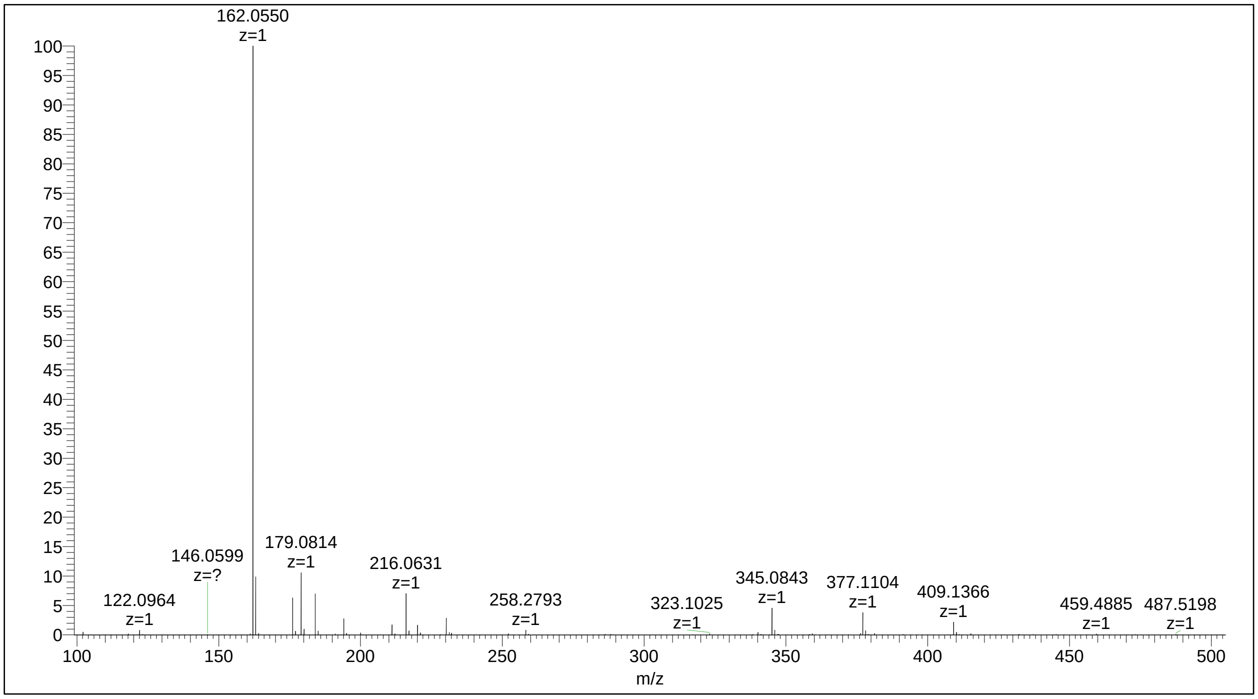

Fig. S3 HRMS (ESI) spectrum of 1-methylindoline-2,3-dione (**4a**)

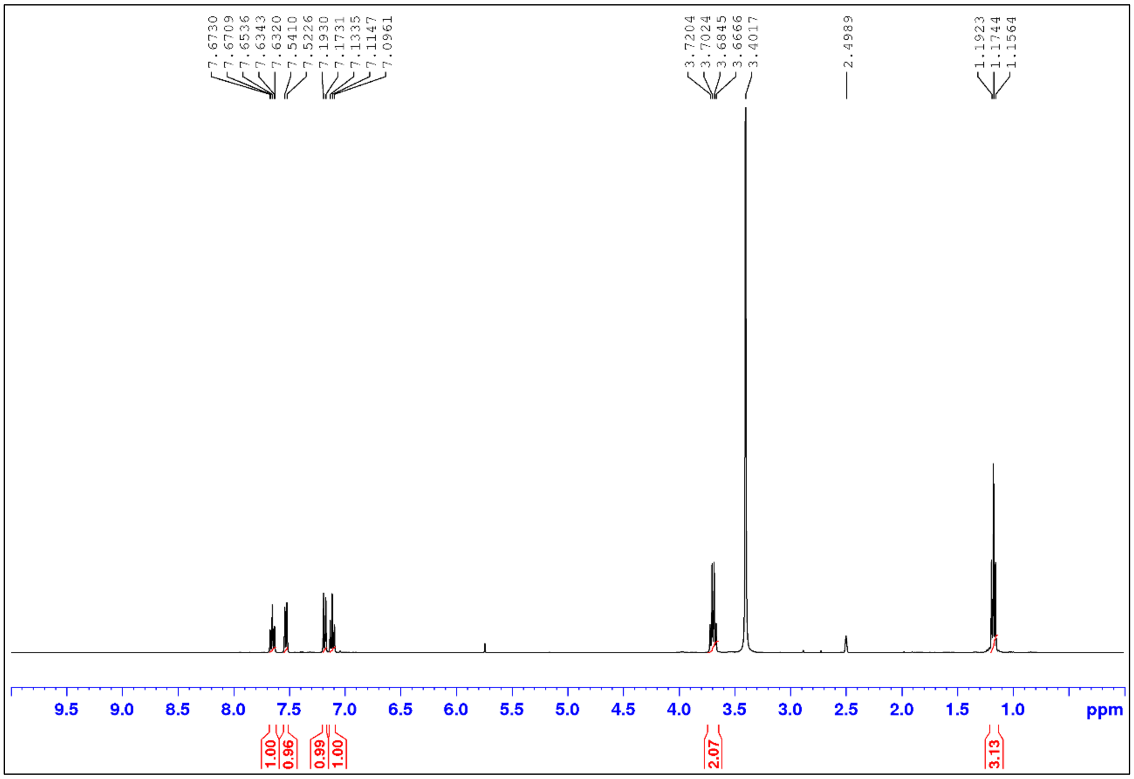

Fig. S4 ^{1}H NMR (DMSO-d_6, 400 MHz) spectrum of 1-ethylindoline-2,3-dione (**4b**)

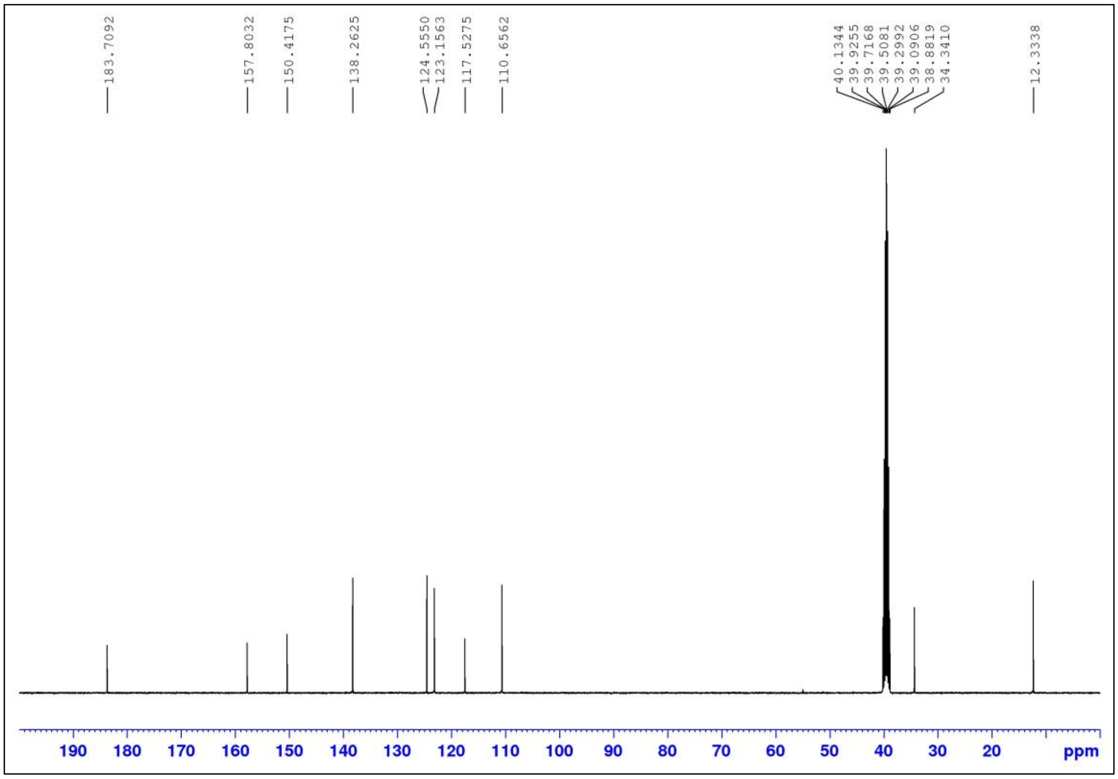

Fig. S5 ^{13}C NMR (DMSO-d_6, 100 MHz) spectrum of 1-ethylindoline-2,3-dione (**4b**)

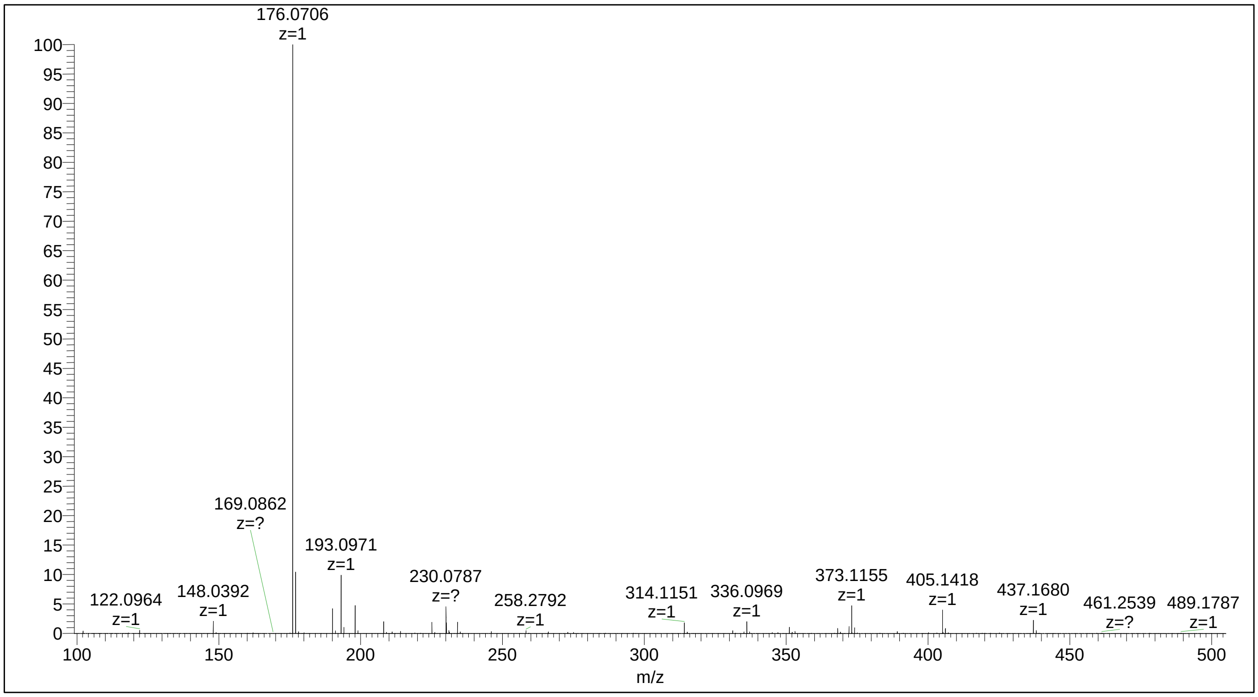

Fig. S6 HRMS (ESI) spectrum of 1-ethylindoline-2,3-dione (**4b**)

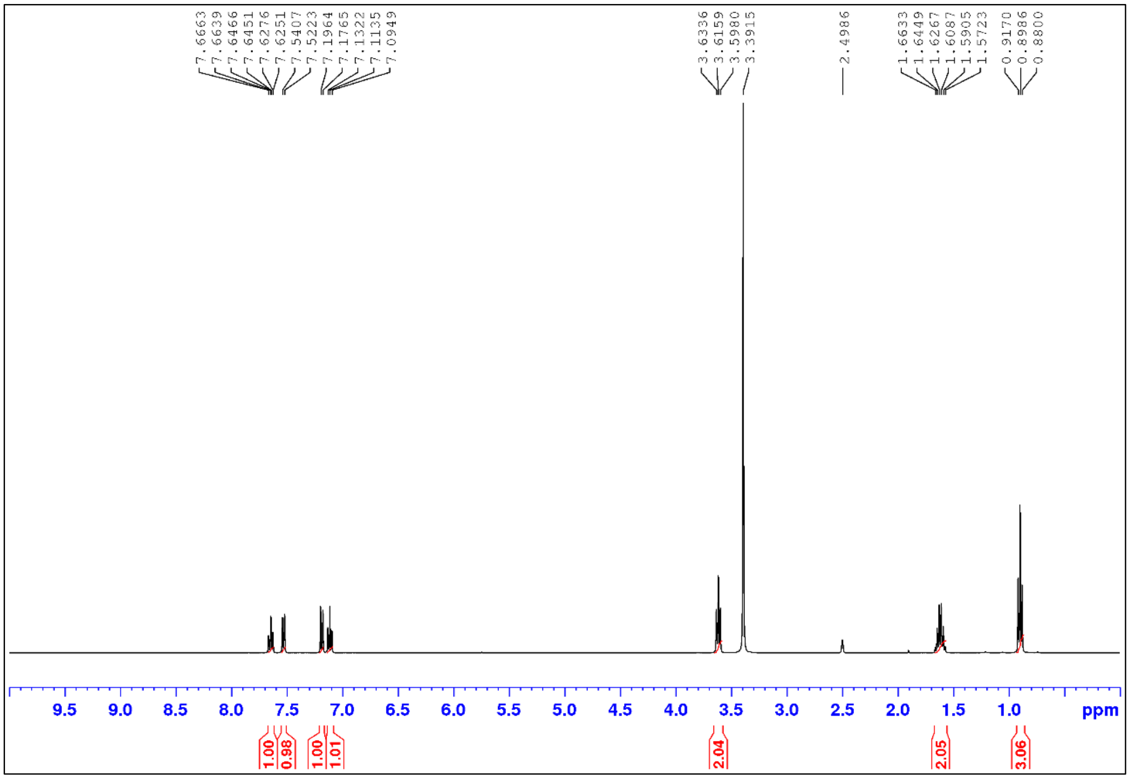

Fig. S7 ^{1}H NMR (DMSO-d_6, 400 MHz) spectrum of 1-propylindoline-2,3-dione (**4c**)

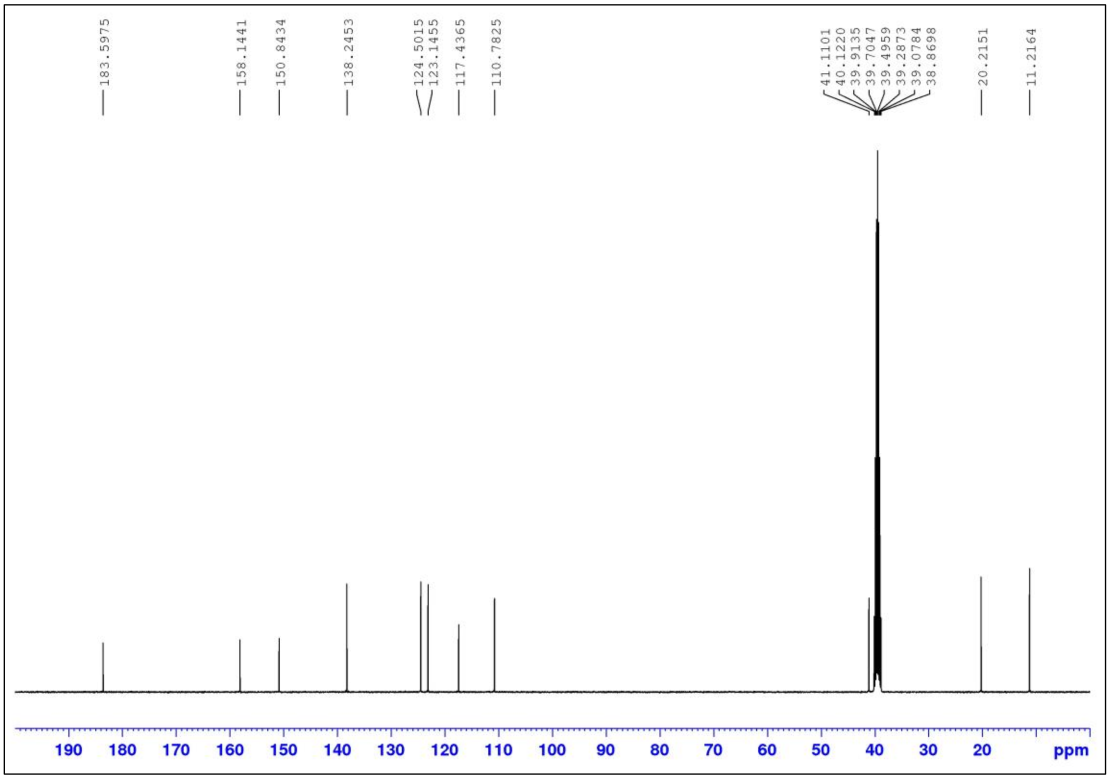

Fig. S8 ^{13}C NMR (DMSO-d_6, 100 MHz) spectrum of 1-propylindoline-2,3-dione (**4c**)

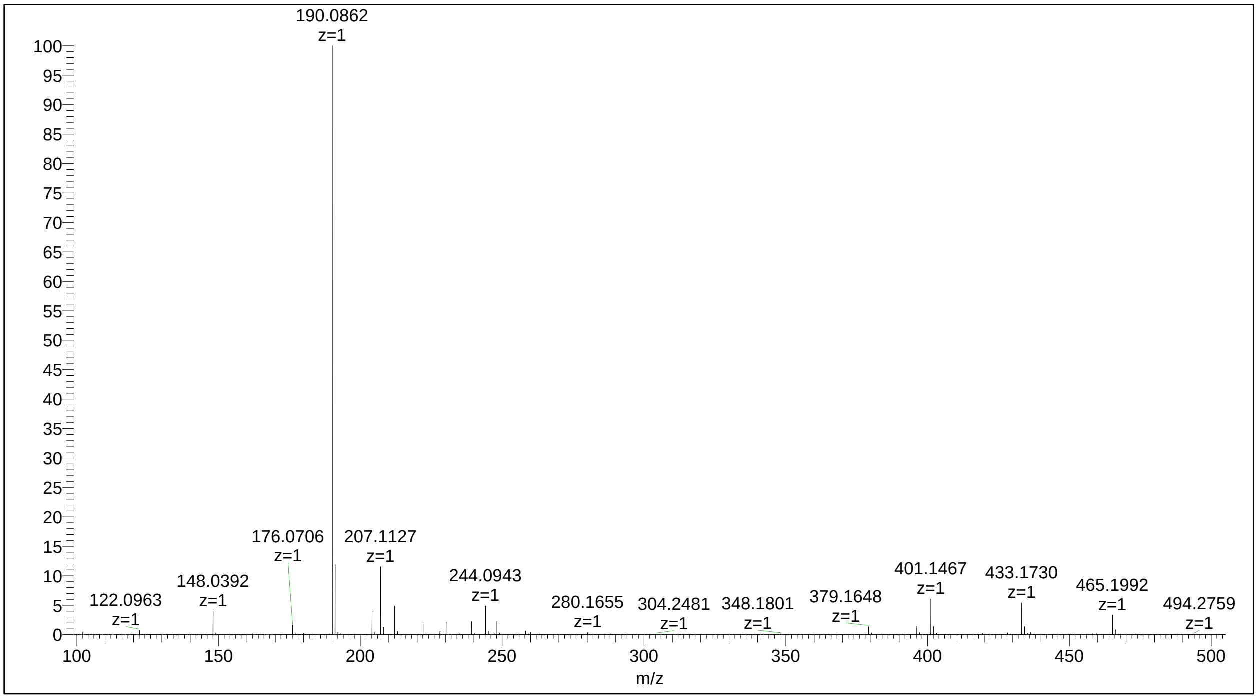

Fig. S9 HRMS (ESI) spectrum of 1-propylindoline-2,3-dione (**4c**)

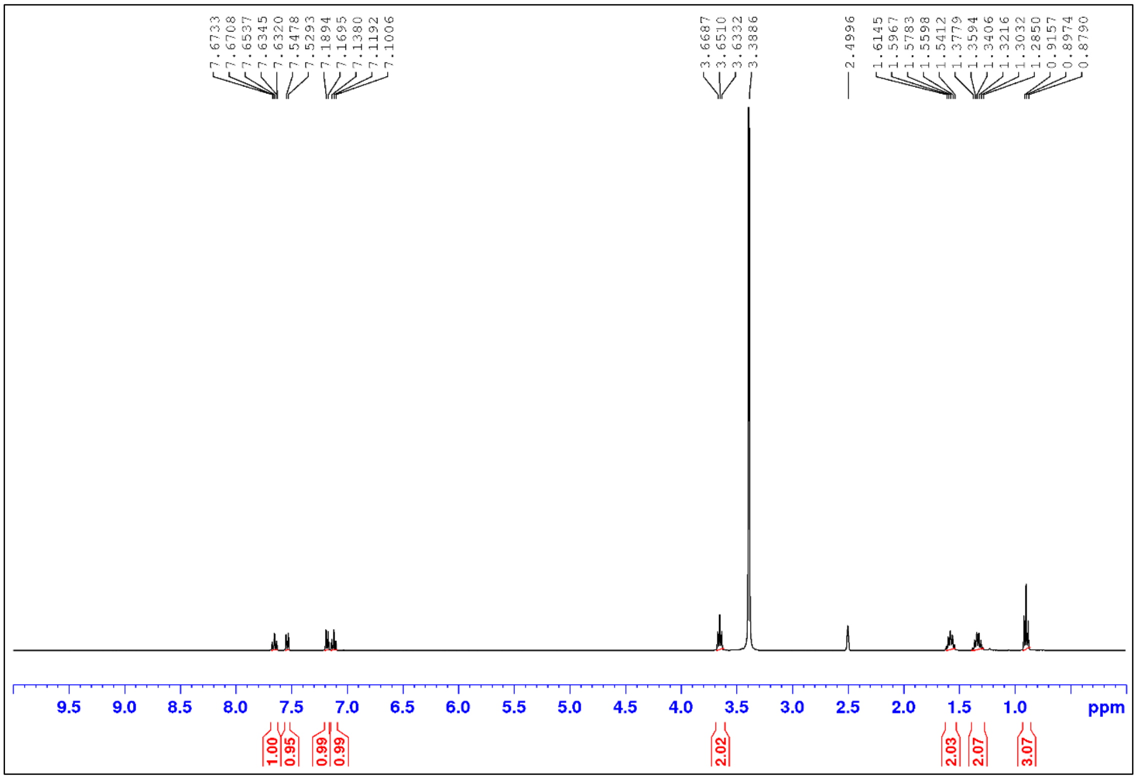

Fig. S10 ^{1}H NMR (DMSO-d_6, 400 MHz) spectrum of 1-butylindoline-2,3-dione (**4d**)

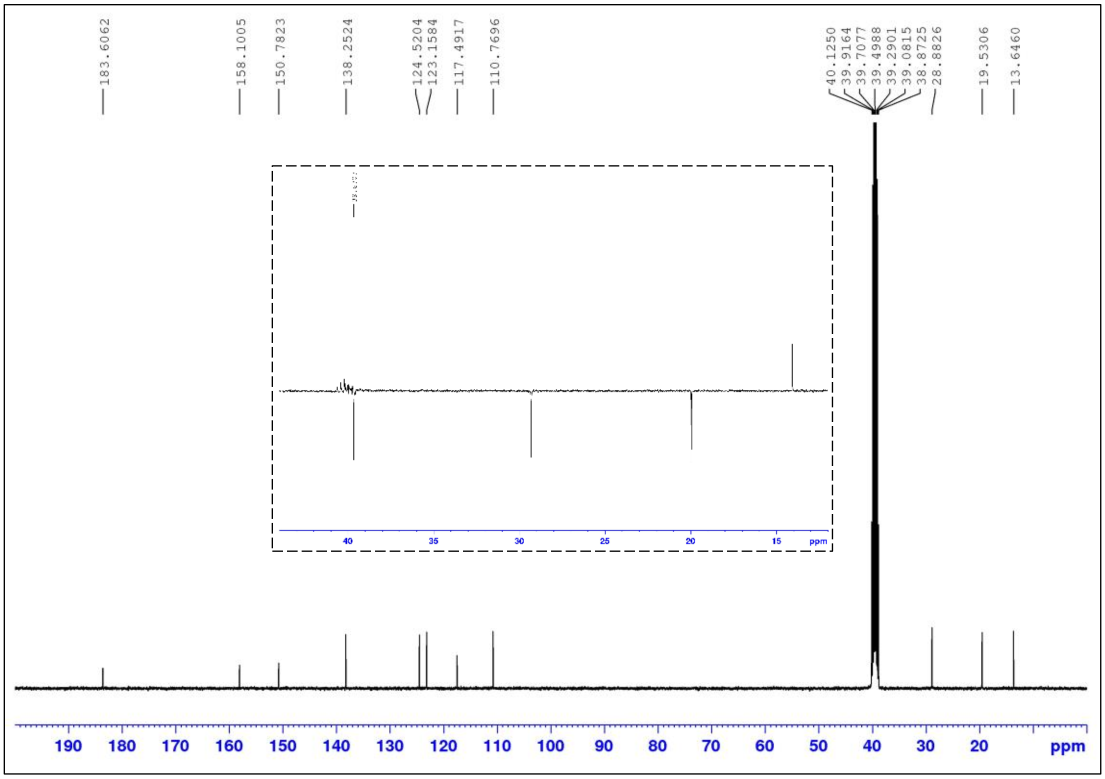

Fig. S11 ^{13}C NMR (DMSO-d_6, 100 MHz) spectrum of 1-butylindoline-2,3-dione (**4d**)
(Inset: DEPT135 showing negative signal for CH_2 peak obscured by DMSO in main spectrum)

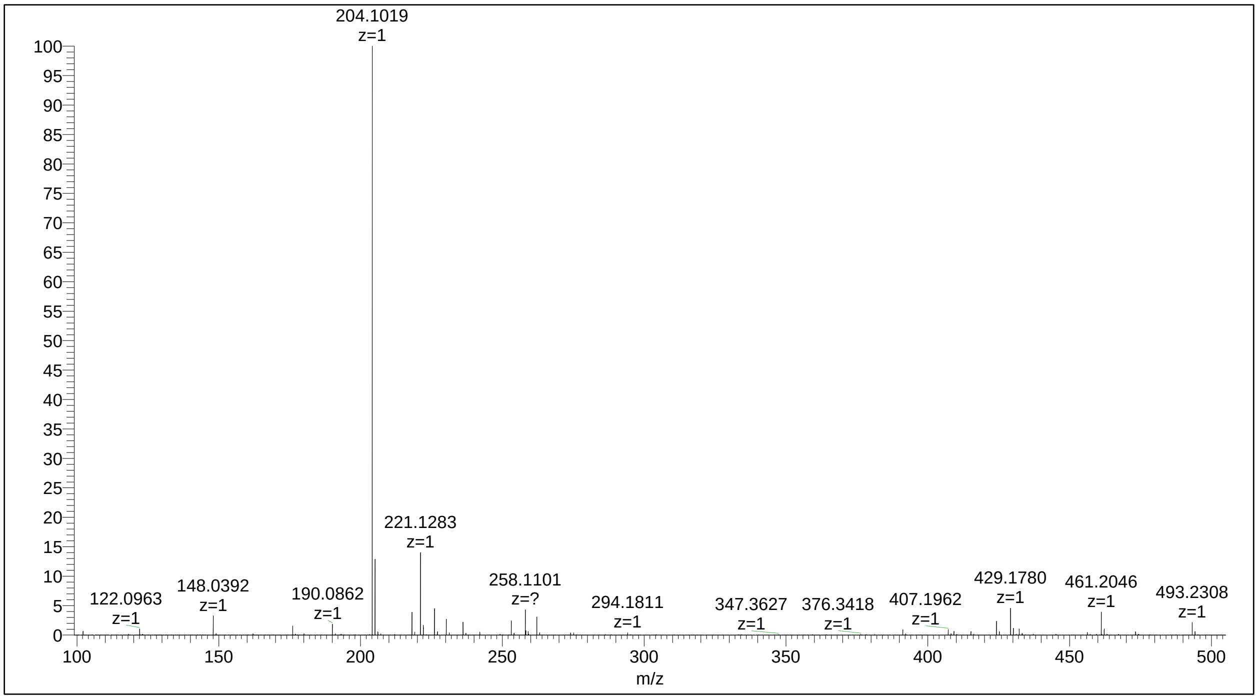

Fig. S12 HRMS (ESI) spectrum of 1-butylindoline-2,3-dione (**4d**)

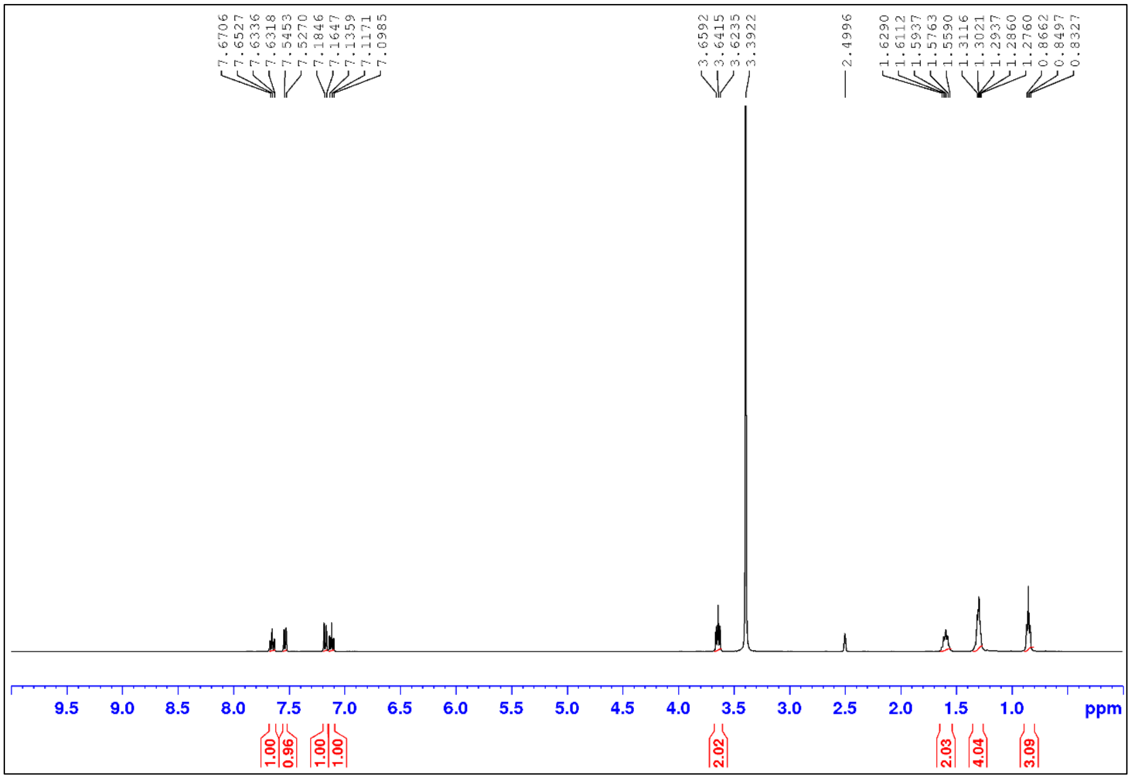

Fig. S13 ^{1}H NMR (DMSO-d_6, 400 MHz) spectrum of 1-pentylindoline-2,3-dione (**4e**)

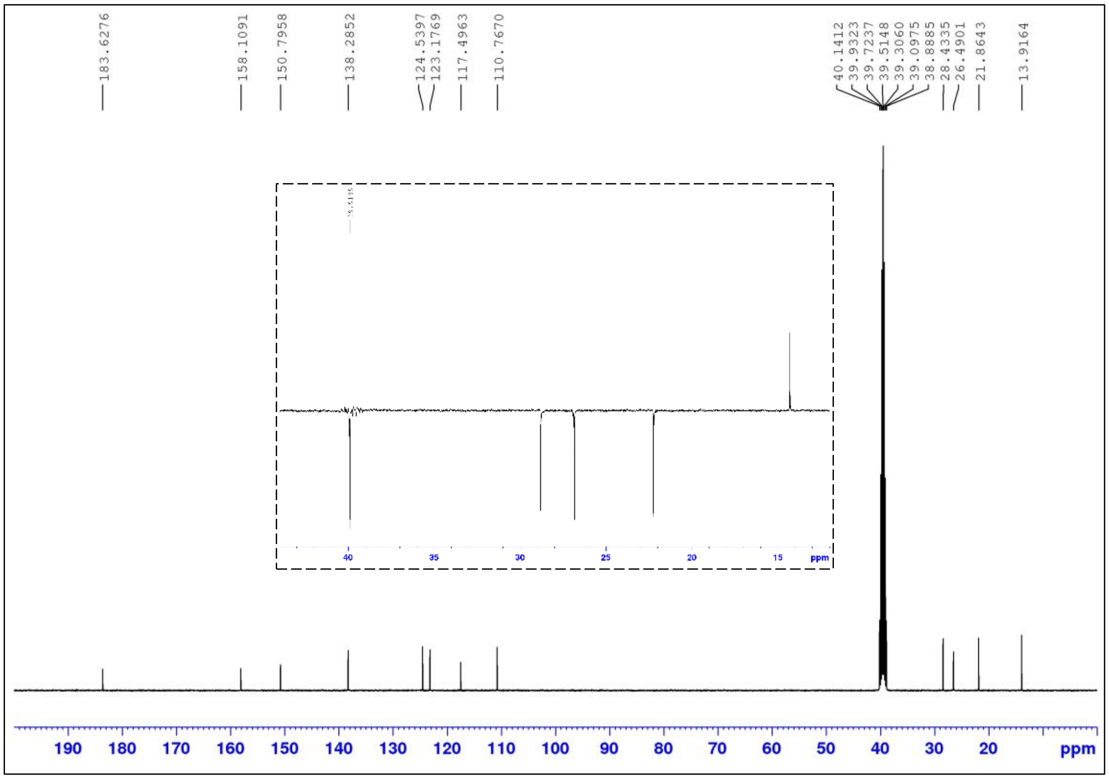

Fig. S14 ^{13}C NMR (DMSO-d_6, 100 MHz) spectrum of 1-pentylindoline-2,3-dione (**4e**)
(Inset: DEPT135 showing negative signal for CH_2 peak obscured by DMSO in main spectrum)

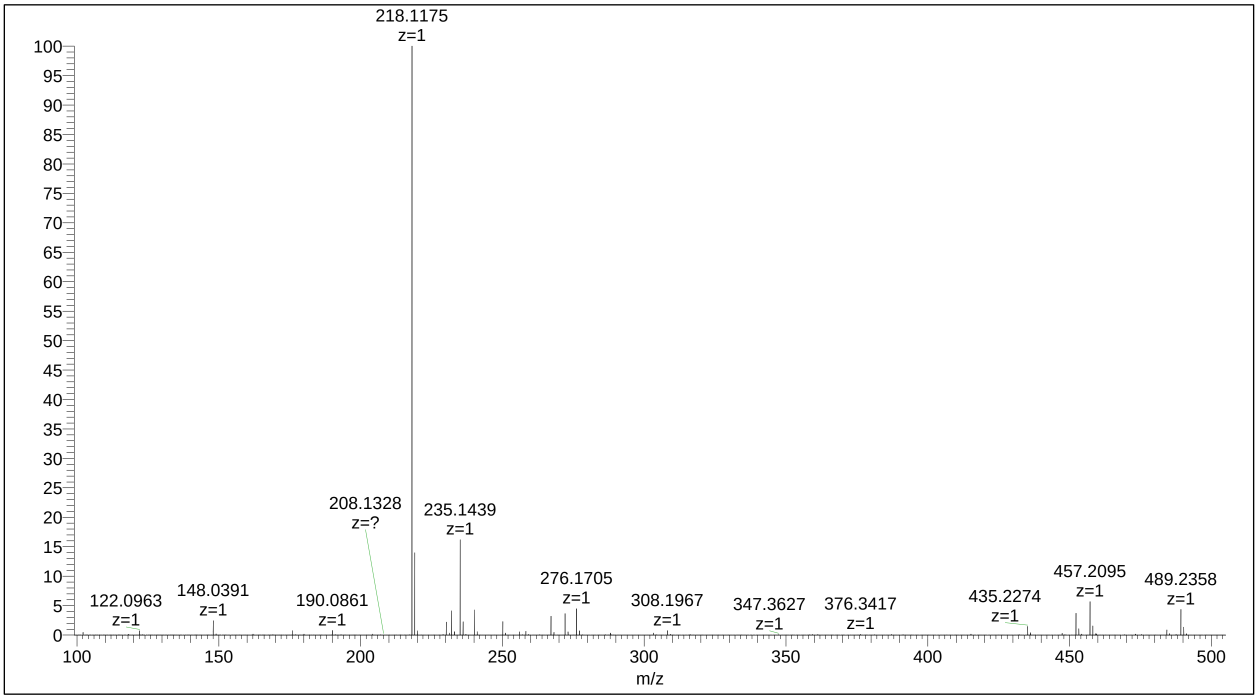

Fig. S15 HRMS (ESI) spectrum of 1-pentylindoline-2,3-dione (**4e**)

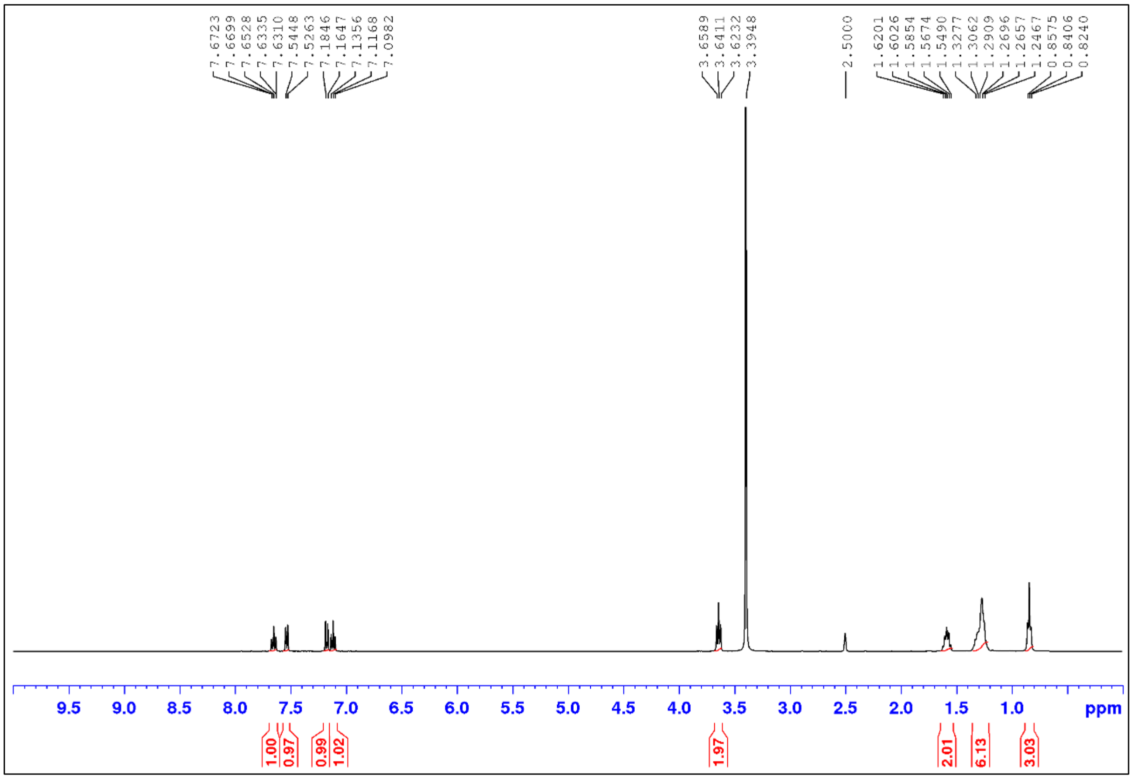

Fig. S16 ¹H NMR (DMSO-d_6, 400 MHz) spectrum of 1-hexylindoline-2,3-dione (**4f**)

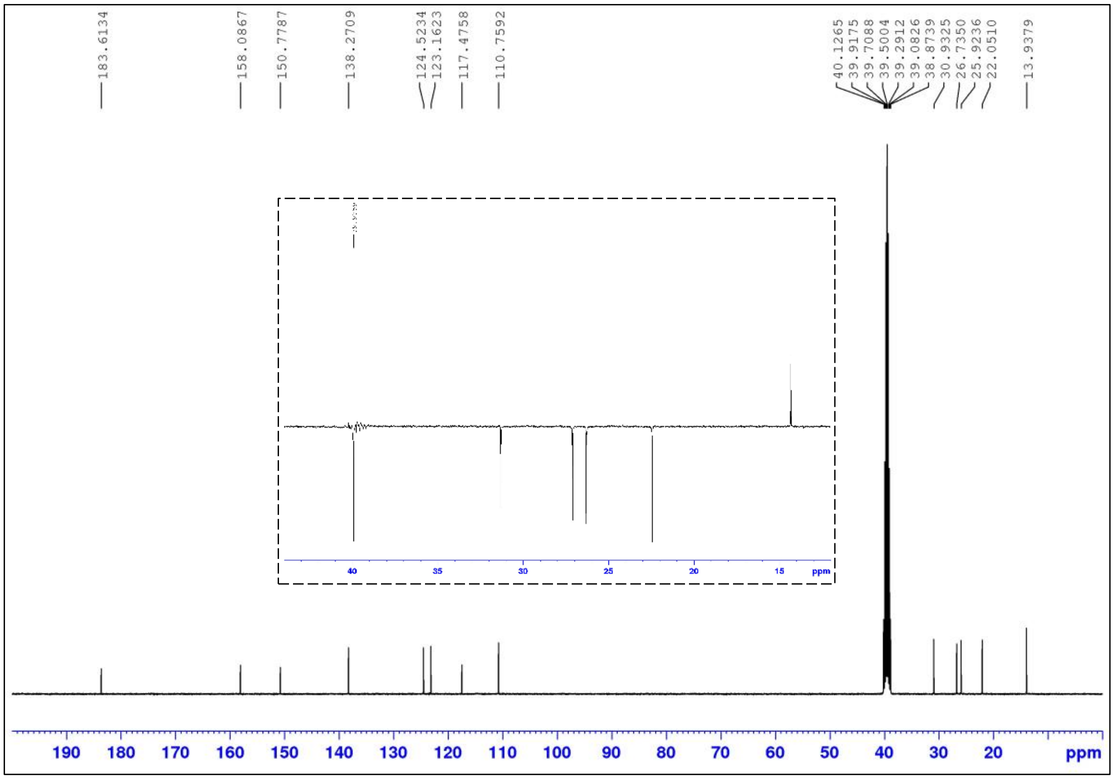

Fig. S17 ¹³C NMR (DMSO-d_6, 100 MHz) spectrum of 1-hexylindoline-2,3-dione (**4f**)
(Inset: DEPT135 showing negative signal for CH_2 peak obscured by DMSO in main spectrum)

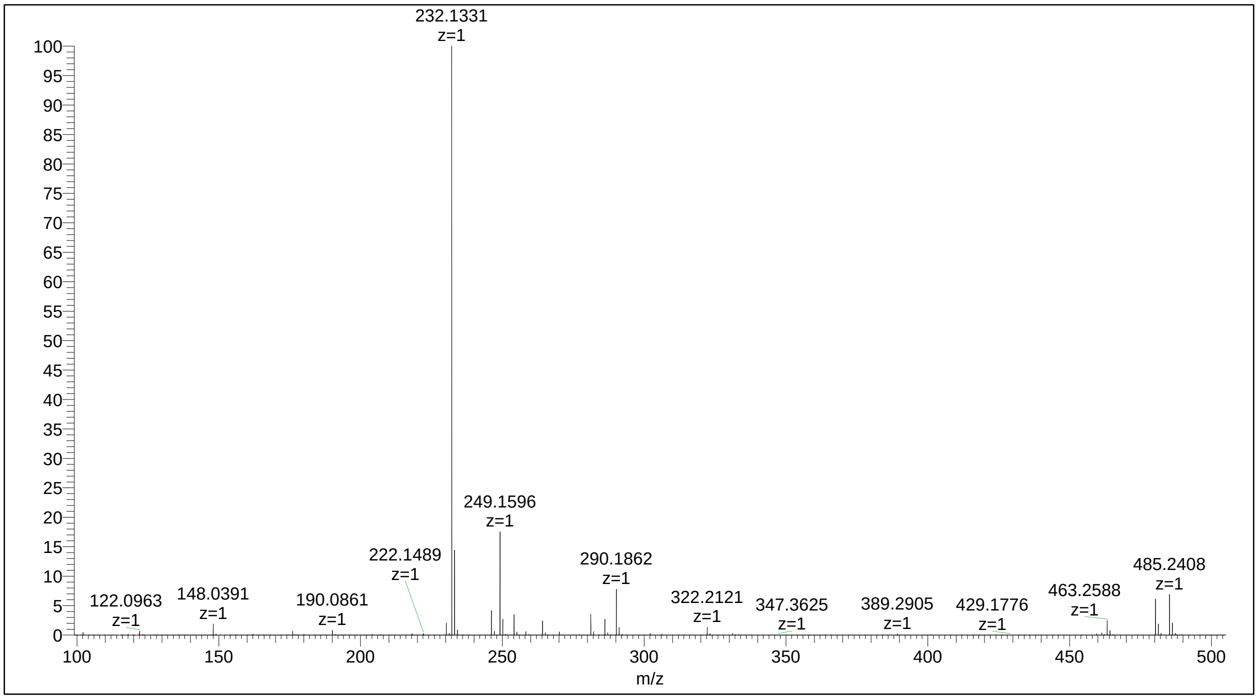

Fig. S18 HRMS (ESI) spectrum of 1-hexylindoline-2,3-dione (**4f**)

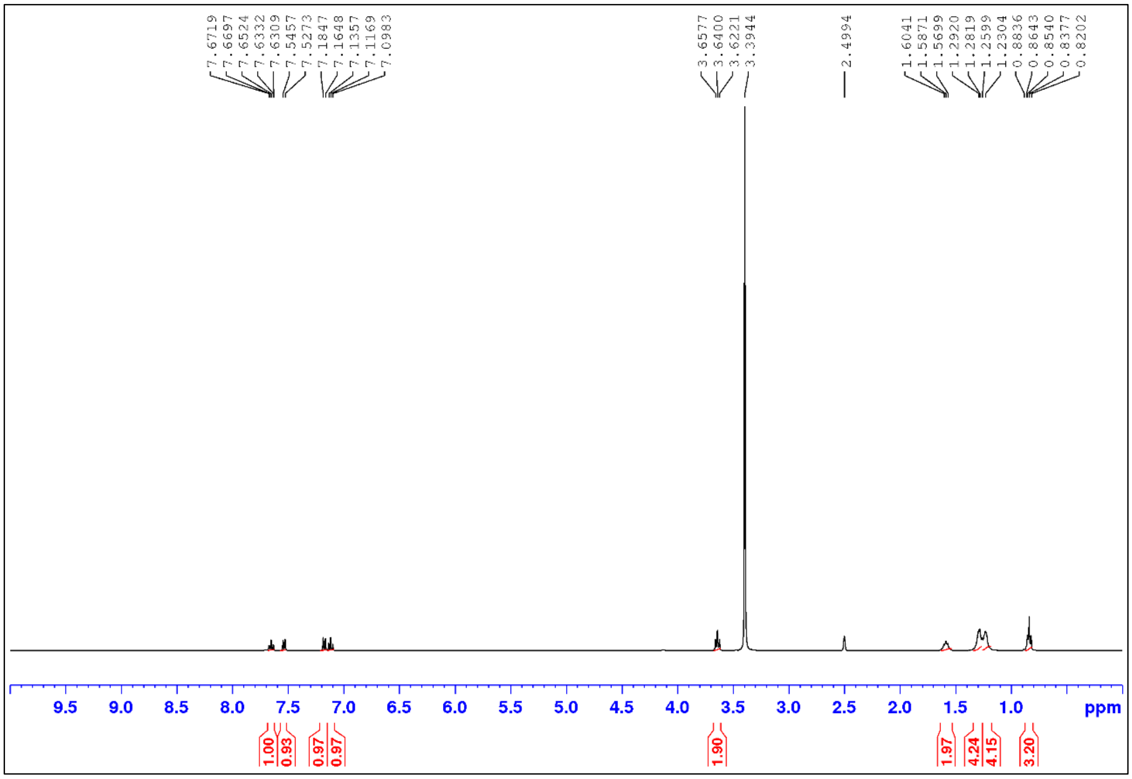

Fig. S19 ^{1}H NMR (DMSO-d_6, 400 MHz) spectrum of 1-heptylindoline-2,3-dione (**4g**)

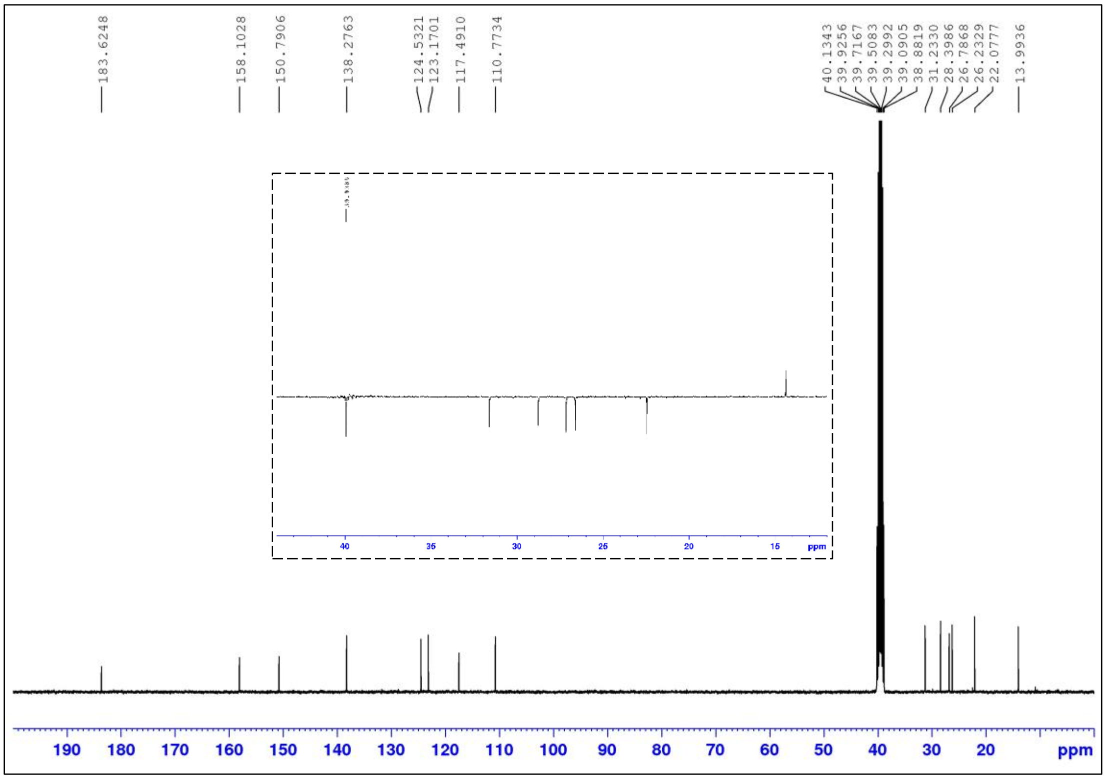

Fig. S20 ^{13}C NMR (DMSO-d_6, 100 MHz) spectrum of 1-heptylindoline-2,3-dione (**4g**)
(Inset: DEPT135 showing negative signal for CH_2 peak obscured by DMSO in main spectrum)

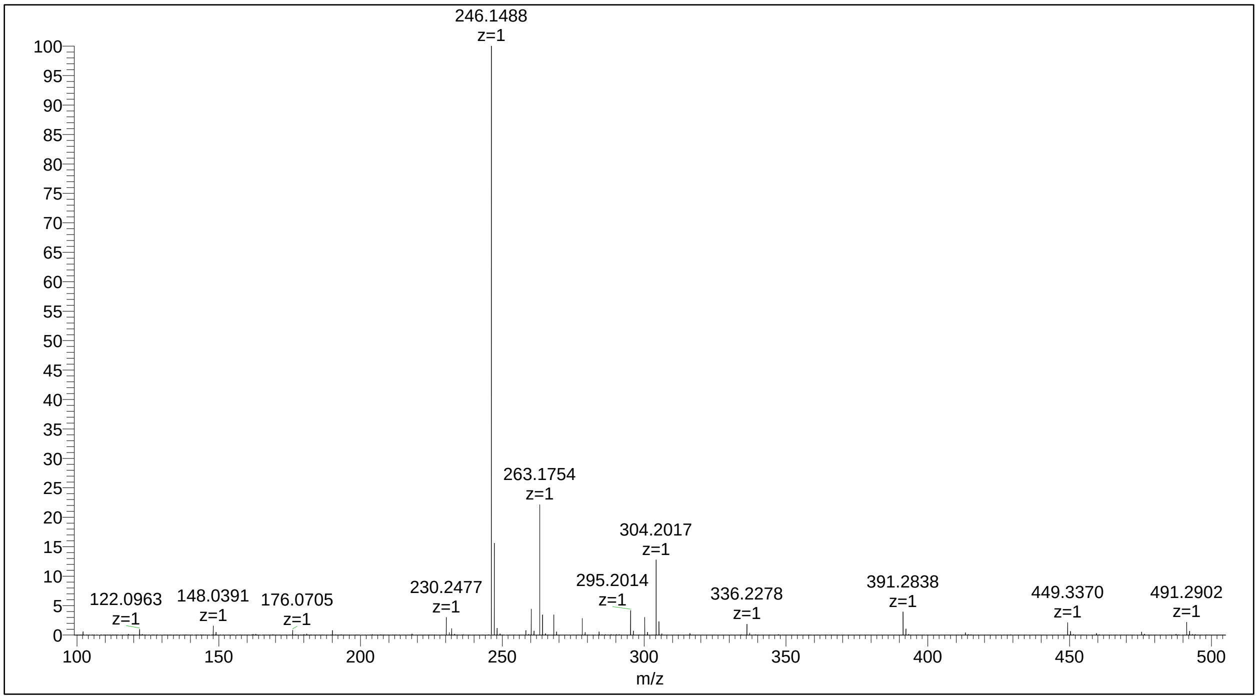

Fig. S21 HRMS (ESI) spectrum of 1-heptylindoline-2,3-dione (**4g**)

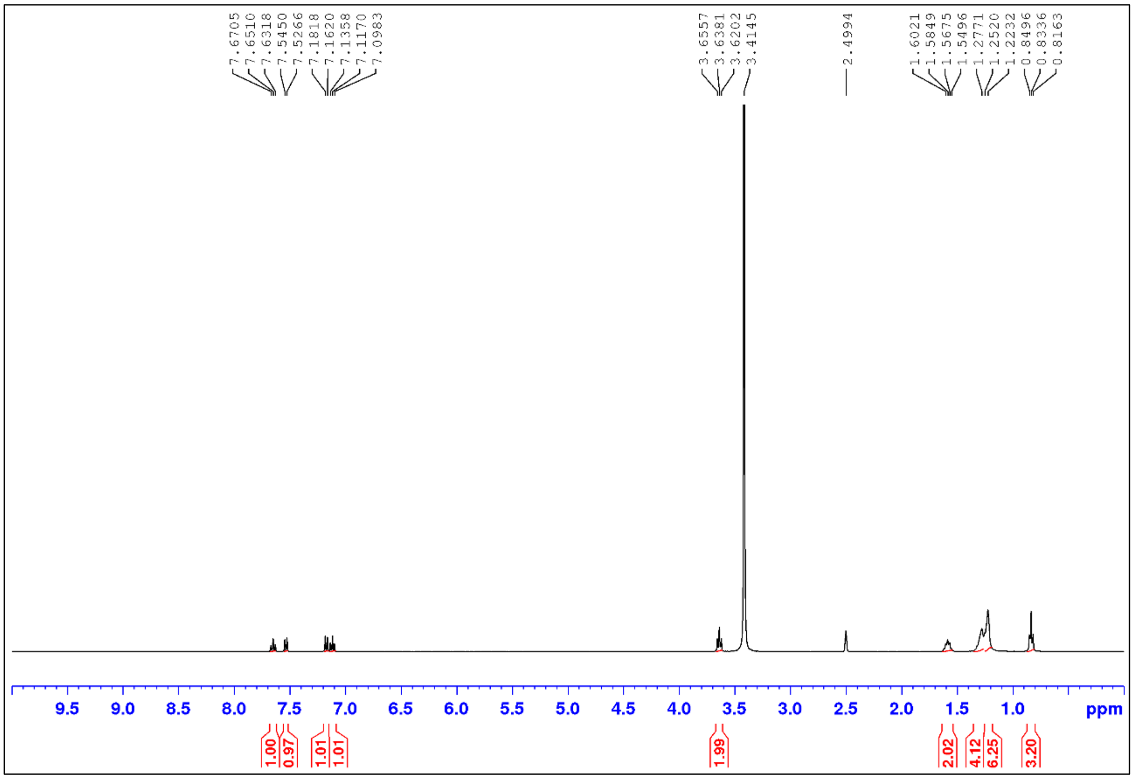

Fig. S22 ^{1}H NMR (DMSO-d_6, 400 MHz) spectrum of 1-octylindoline-2,3-dione (**4h**)

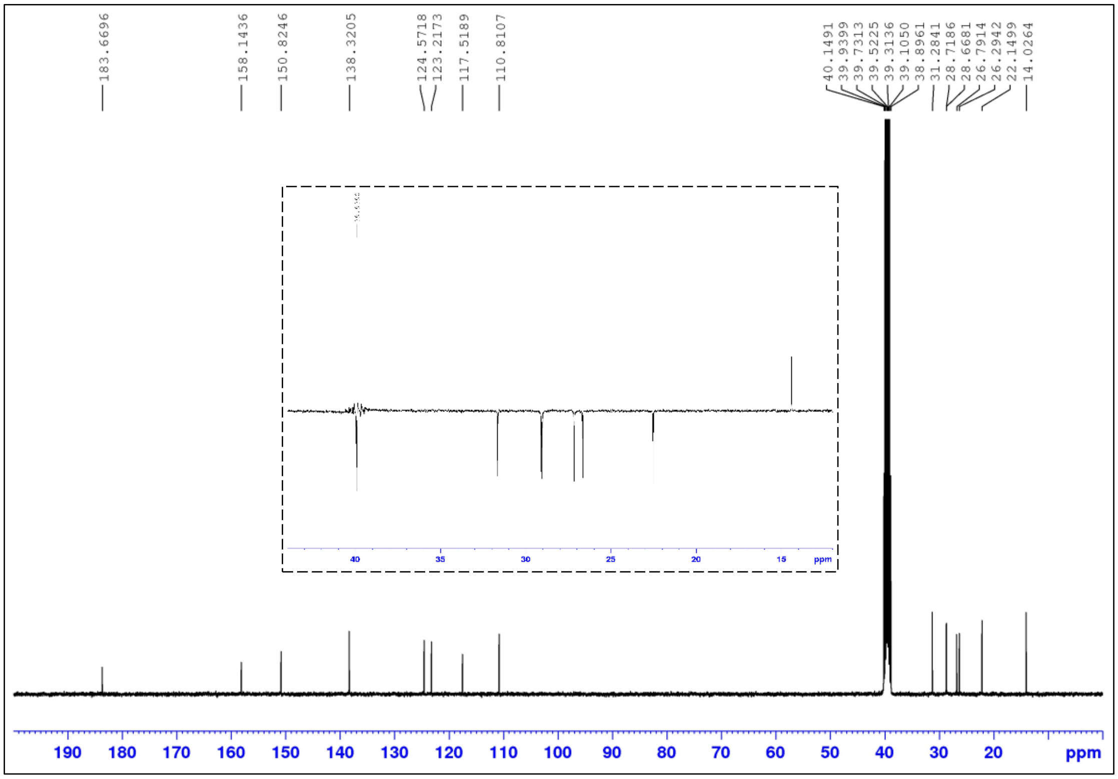

Fig. S23 ^{13}C NMR (DMSO-d_6, 100 MHz) spectrum of 1-octylindoline-2,3-dione (**4h**)
(Inset: DEPT135 showing negative signal for CH_2 peak obscured by DMSO in main spectrum)

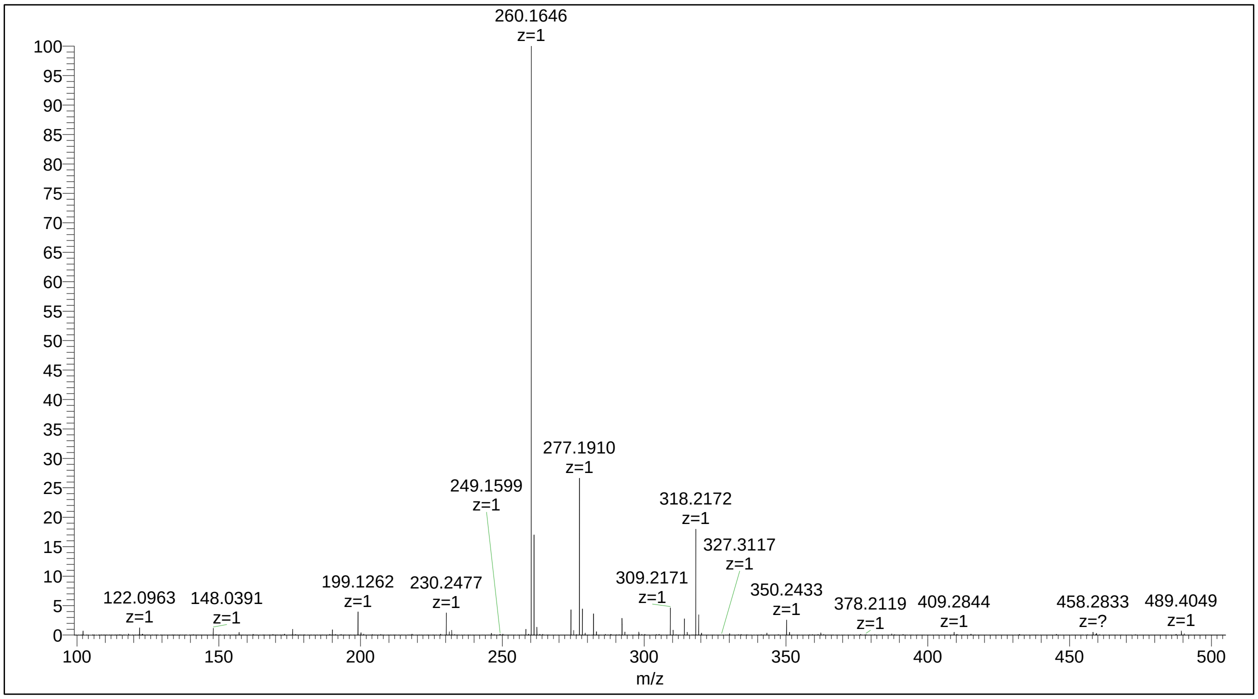

Fig. S24 HRMS (ESI) spectrum of 1-octylindoline-2,3-dione (**4h**)

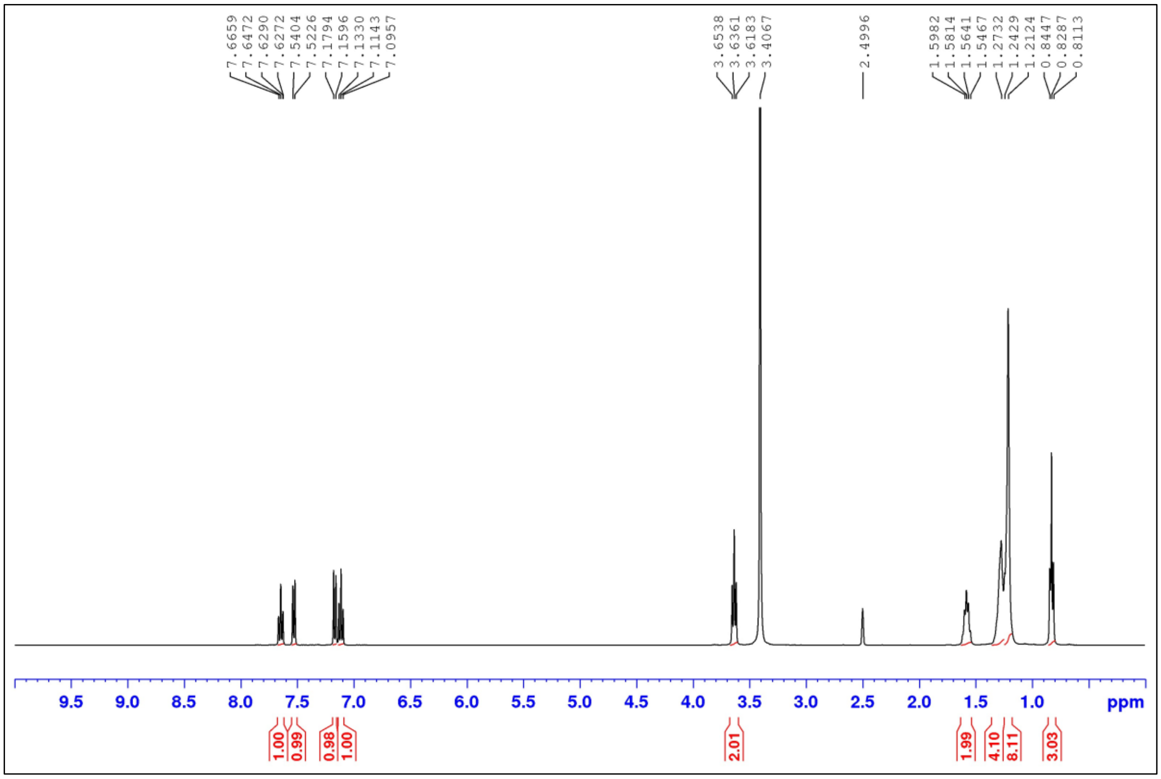

Fig. S25 ^{1}H NMR (DMSO-d_6, 400 MHz) spectrum of 1-nonylindoline-2,3-dione (**4i**)

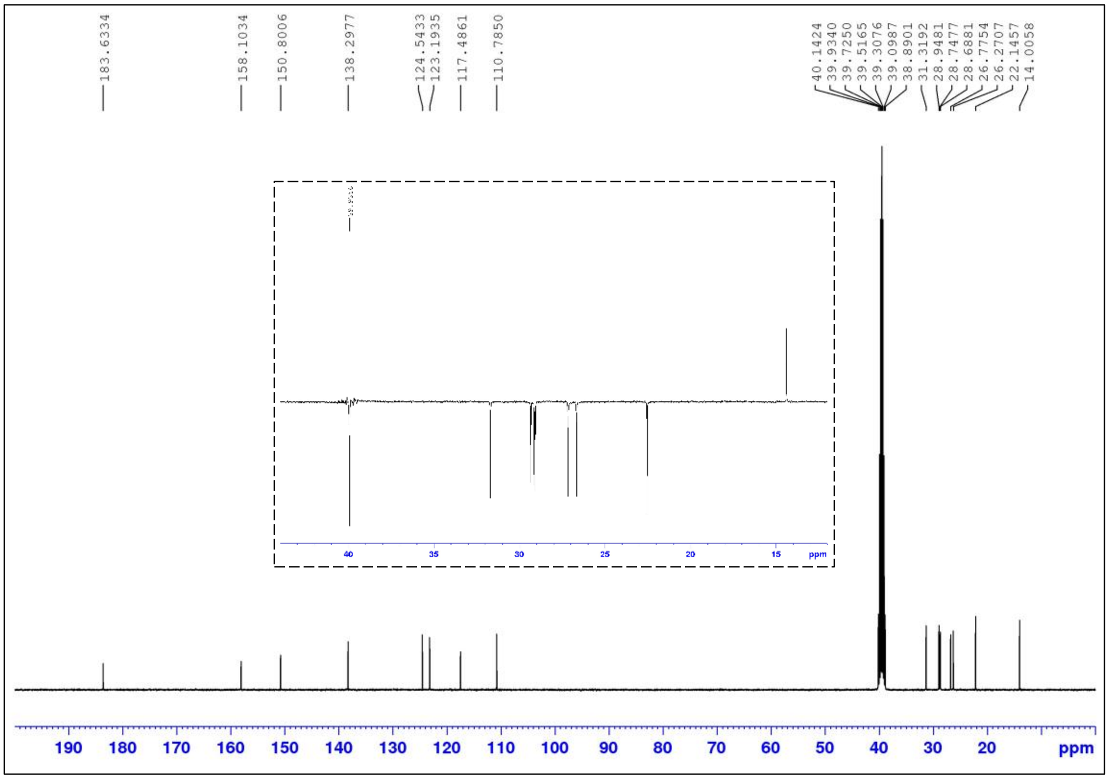

Fig. S26 ^{13}C NMR (DMSO-d_6, 100 MHz) spectrum of 1-nonylindoline-2,3-dione (**4i**)
(Inset: DEPT135 showing negative signal for CH_2 peak obscured by DMSO in main spectrum)

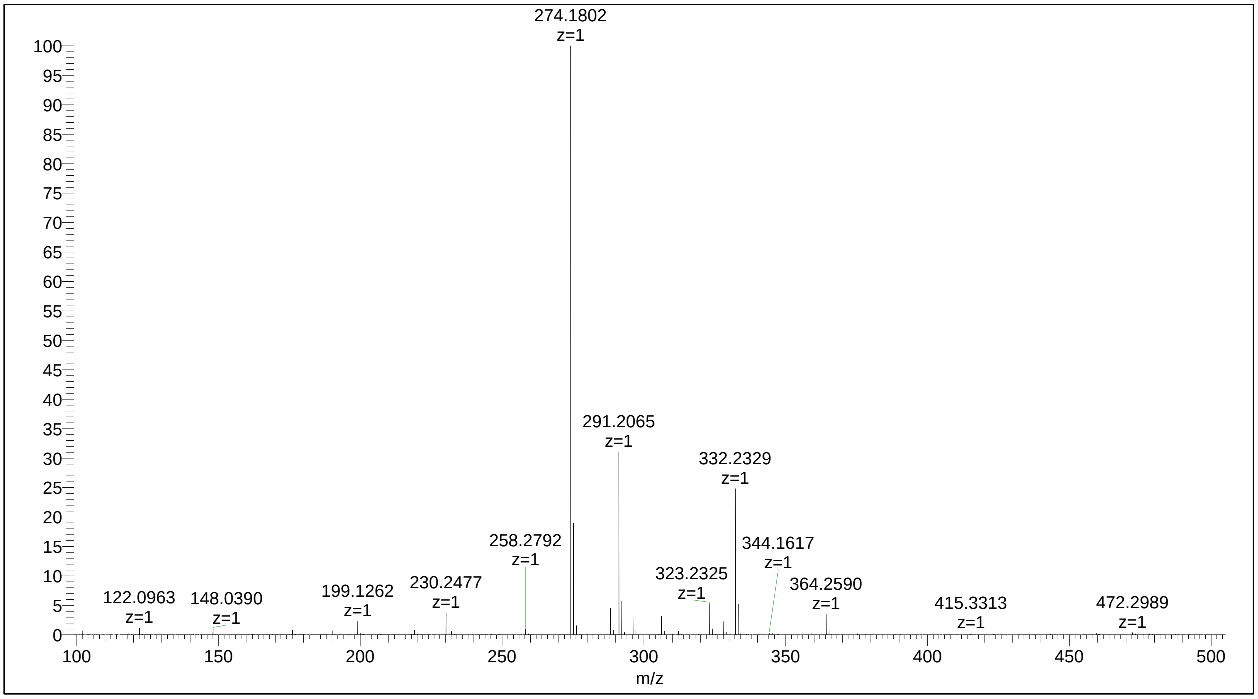

Fig. S27 HRMS (ESI) spectrum of 1-nonylindoline-2,3-dione (**4i**)

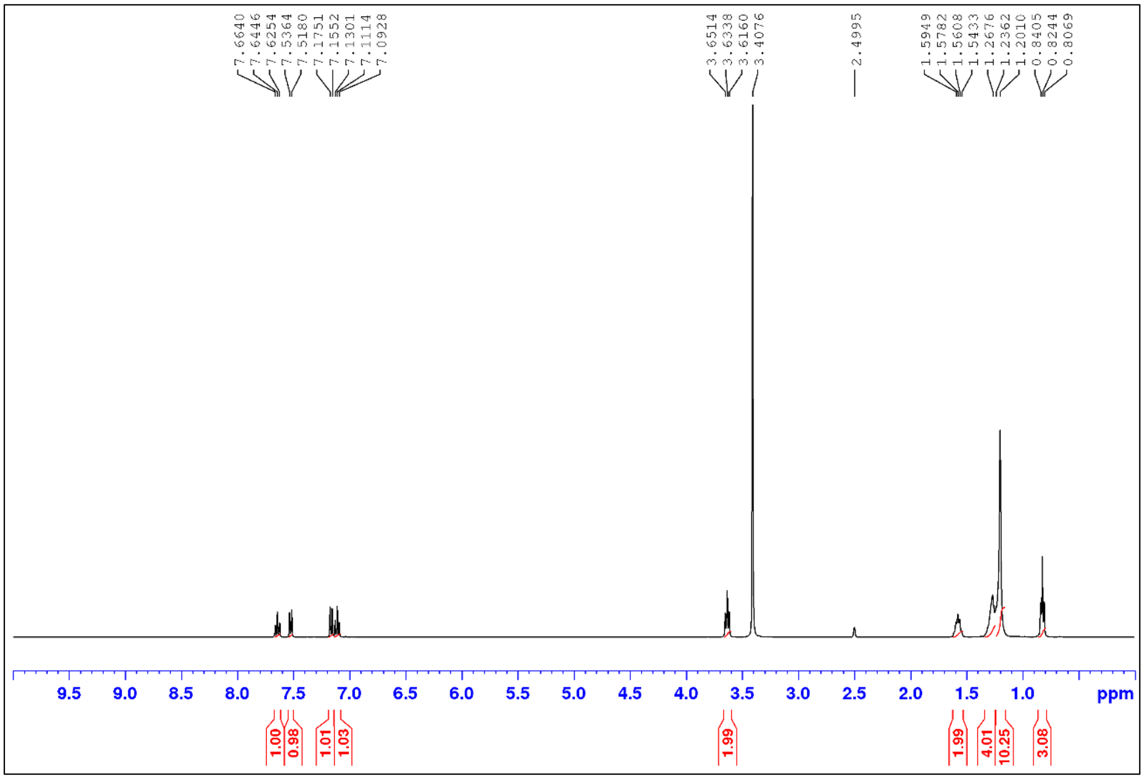

Fig. S28 ^{1}H NMR (DMSO-d_6, 400 MHz) spectrum of 1-decylindoline-2,3-dione (**4j**)

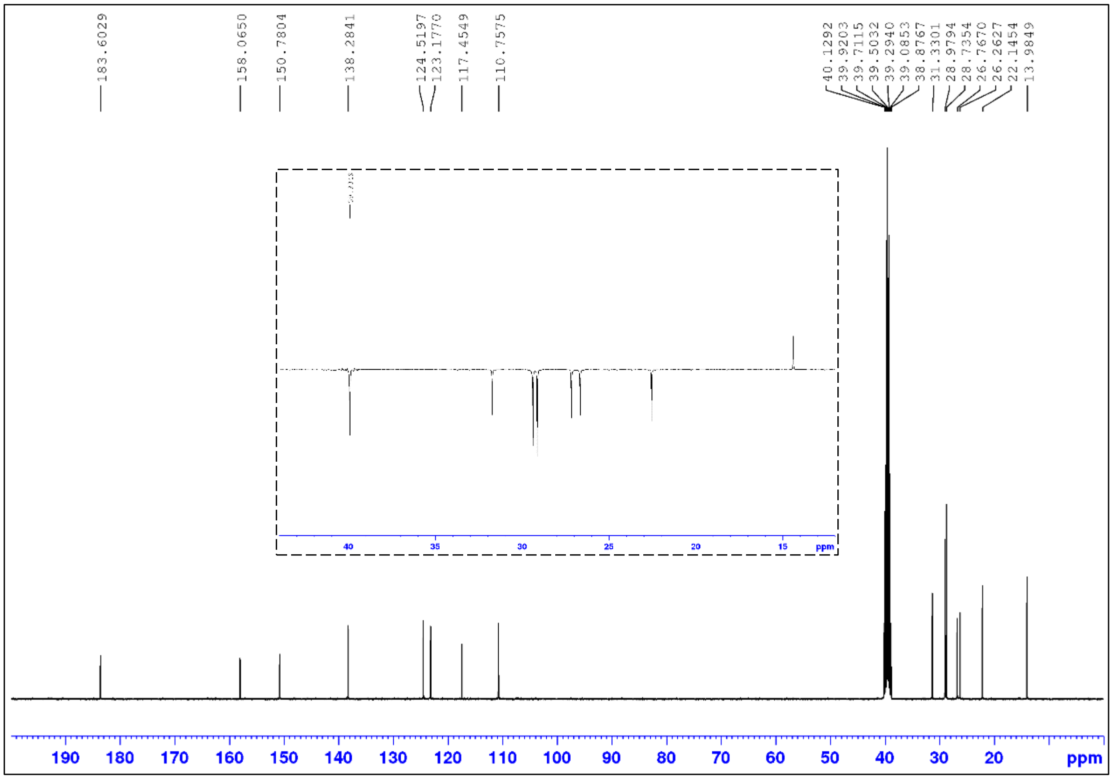

Fig. S29 ^{13}C NMR (DMSO-d_6, 100 MHz) spectrum of 1-decylindoline-2,3-dione (**4j**)
(Inset: DEPT135 showing negative signal for CH_2 peak obscured by DMSO in main spectrum)

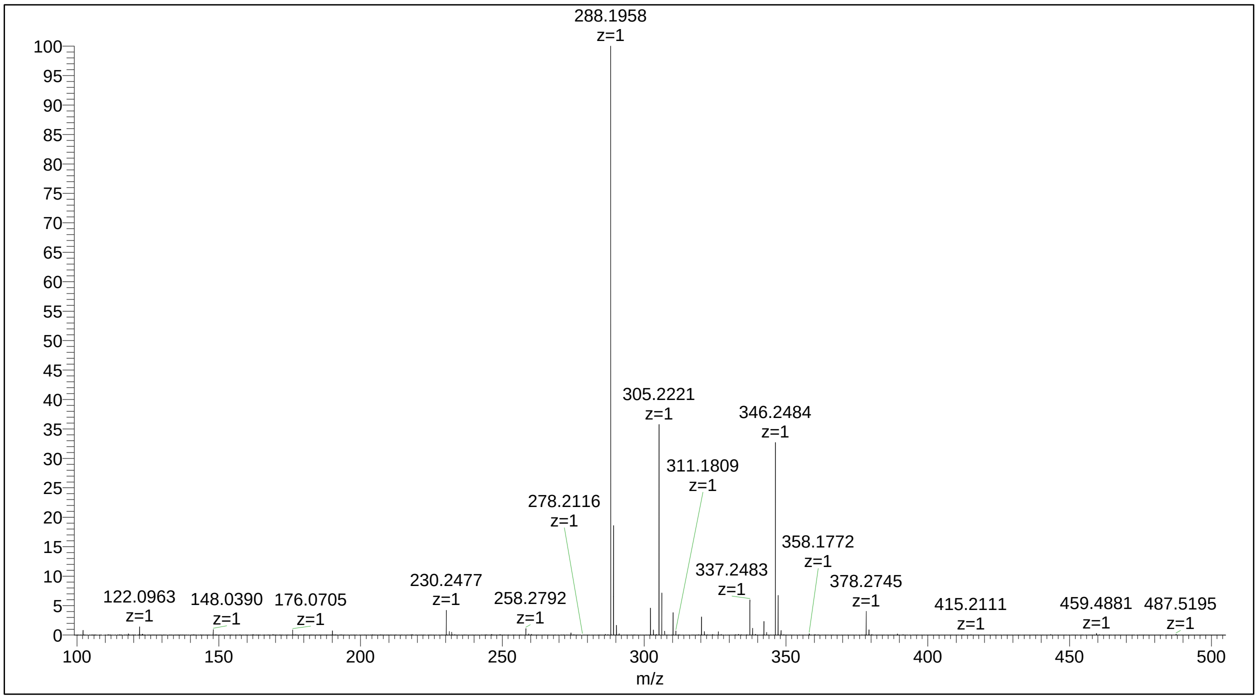

Fig. S30 HRMS (ESI) spectrum of 1-decylindoline-2,3-dione (**4j**)

2. Copies of ^{1}H NMR, ^{13}C NMR, and HRMS spectra for *N*-alkyl indoles 5a-j

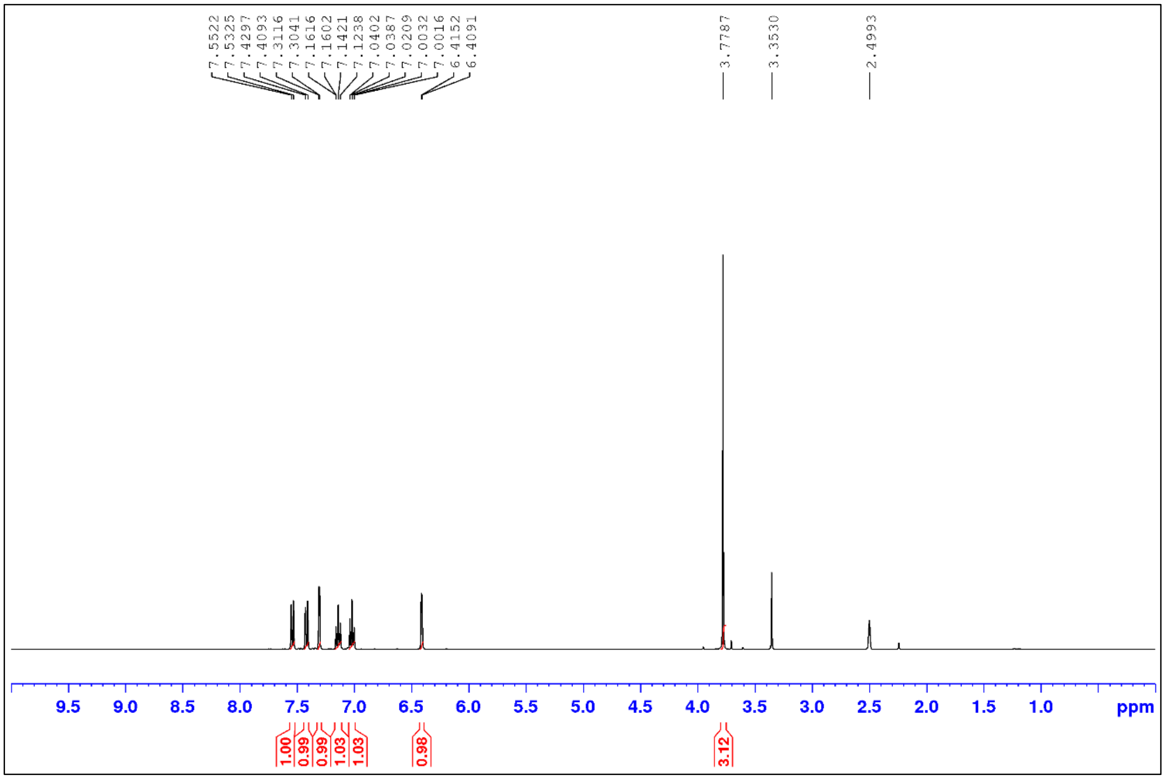

Fig. S31 ^{1}H NMR (DMSO-d_6, 400 MHz) spectrum of 1-methyl-1*H*-indole (**5a**)

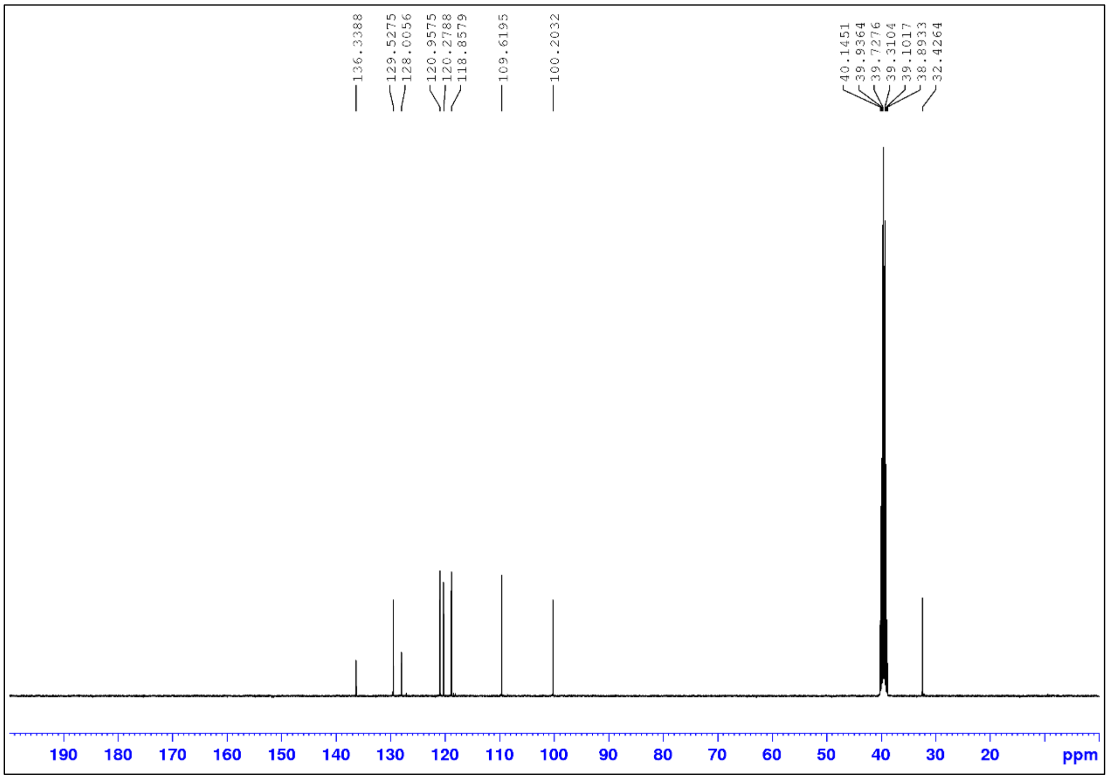

Fig. S32 ^{13}C NMR (DMSO-d_6, 100 MHz) spectrum of 1-methyl-1*H*-indole (**5a**)

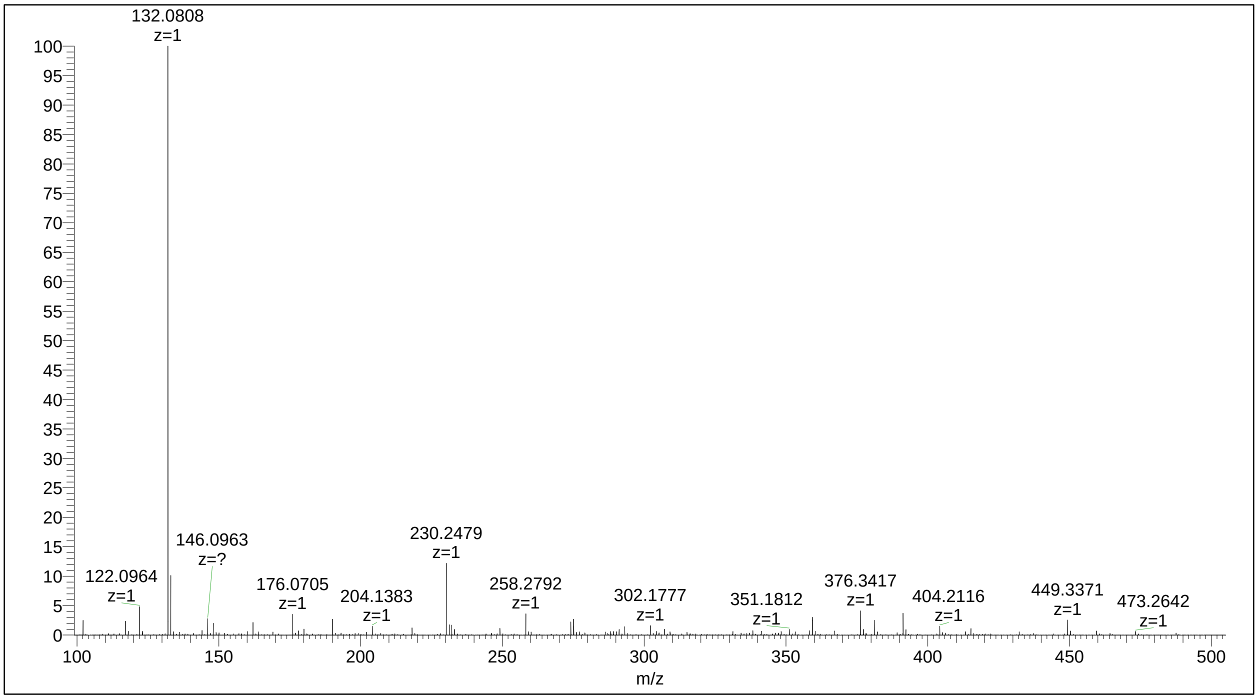

Fig. S33 HRMS (ESI) spectrum of 1-methyl-1*H*-indole (**5a**)

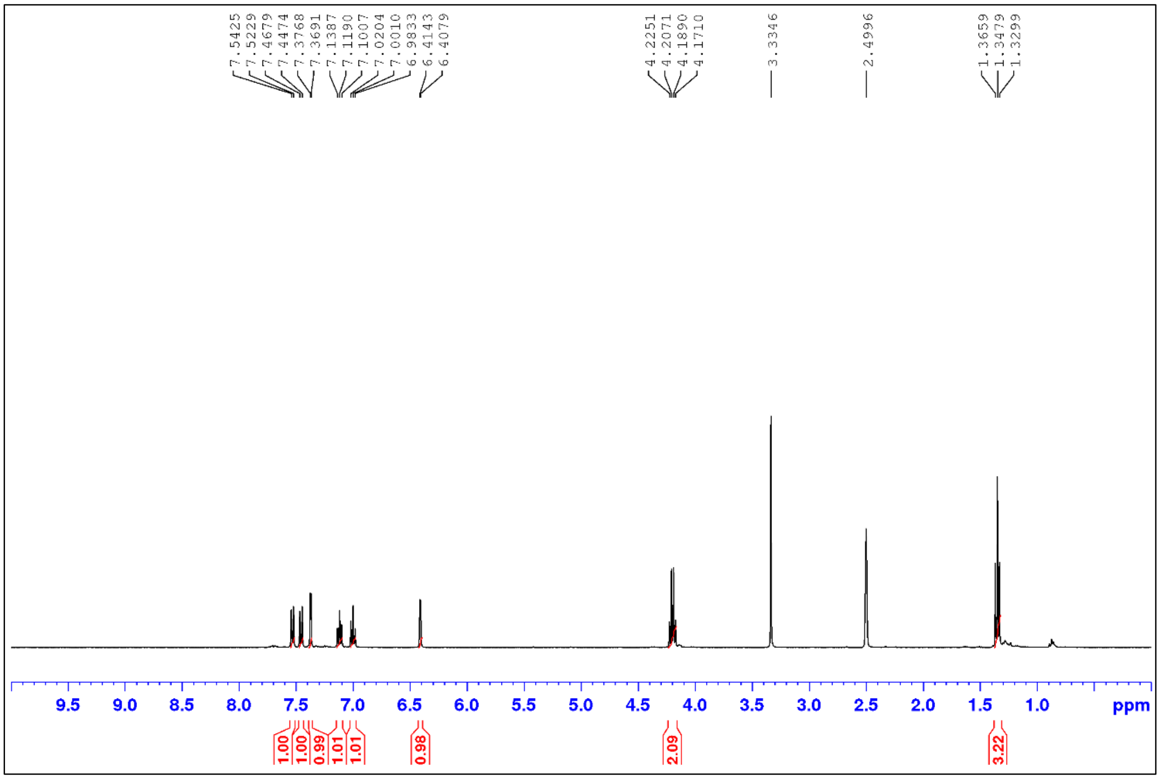

Fig. S34 ^{1}H NMR (DMSO-d_6, 400 MHz) spectrum of 1-ethyl-1*H*-indole (**5b**)

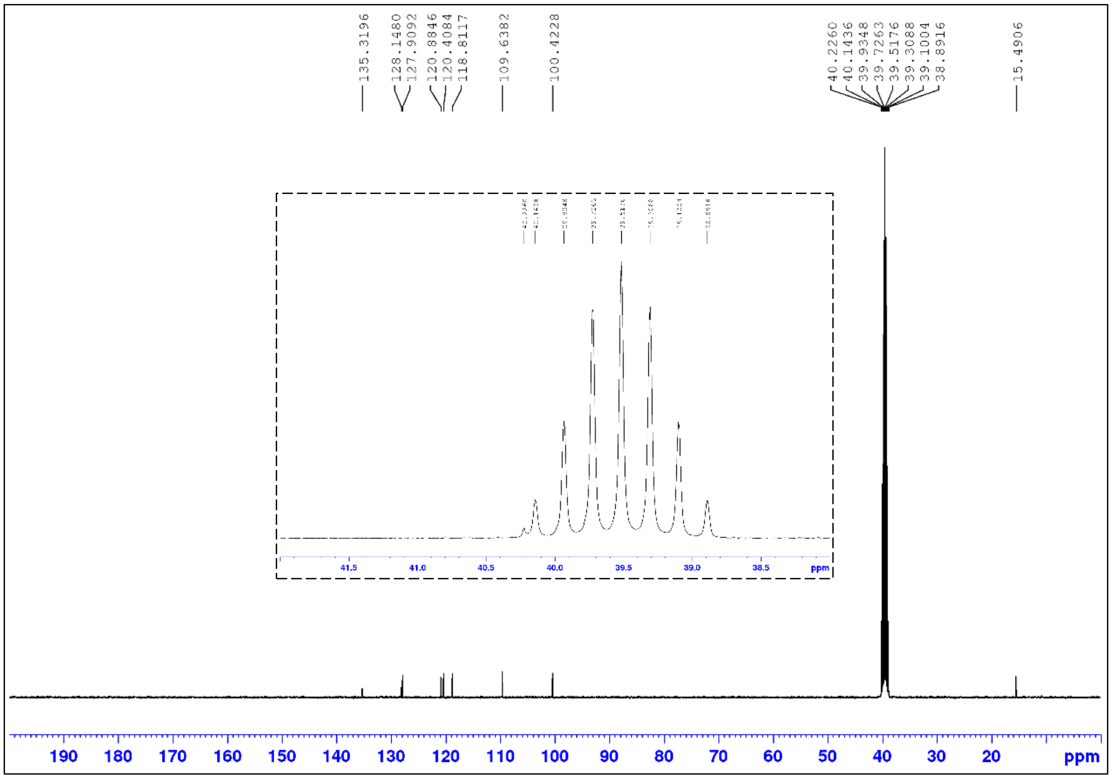

Fig. S35 ^{13}C NMR (DMSO-d_6, 100 MHz) spectrum of 1-ethyl-1*H*-indole (**5b**)
(Inset: Zoom showing CH_2 peak of **5b** at 40.2 ppm)

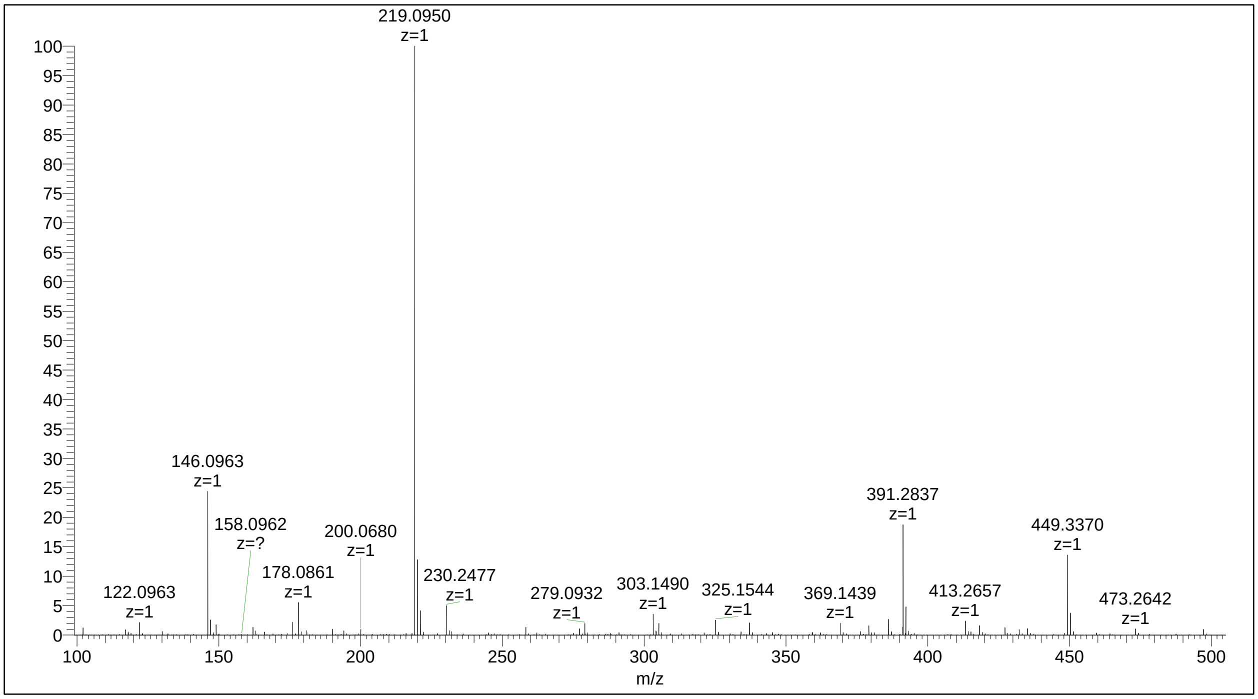

Fig. S36 HRMS (ESI) spectrum of 1-ethyl-1*H*-indole (**5b**)

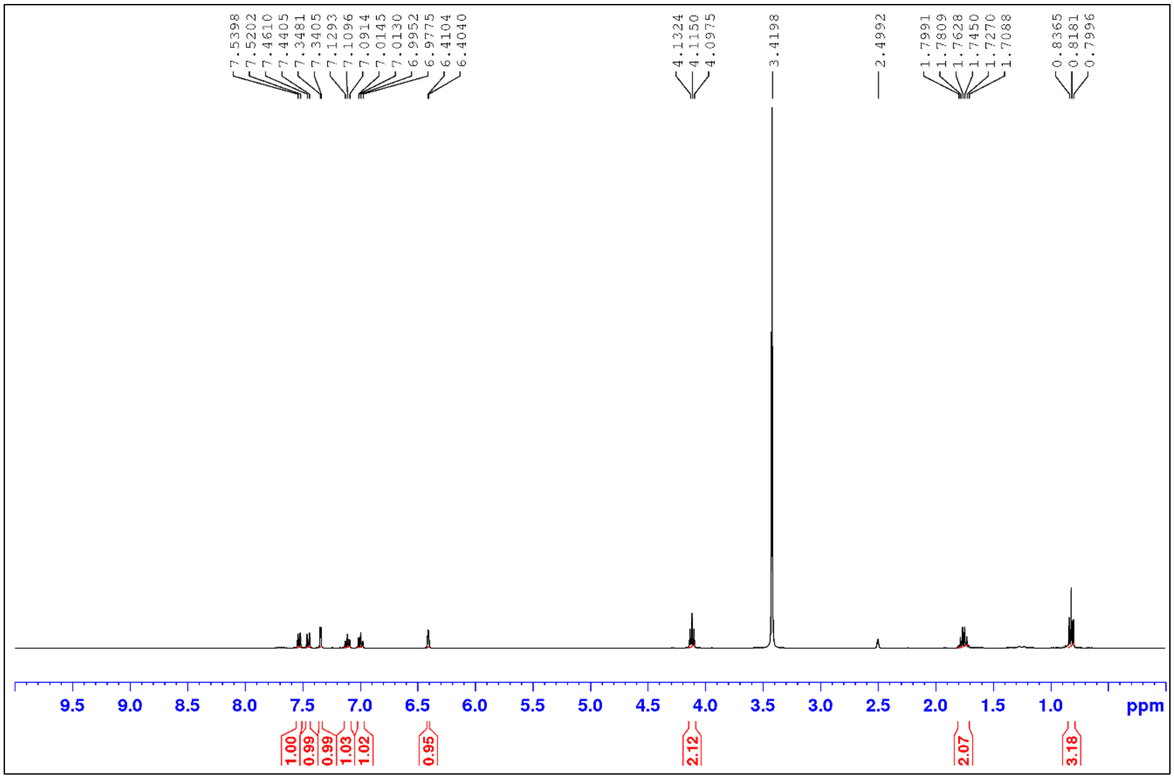

Fig. S37 ^{1}H NMR (DMSO-d_6, 400 MHz) spectrum of 1-propyl-1*H*-indole (**5c**)

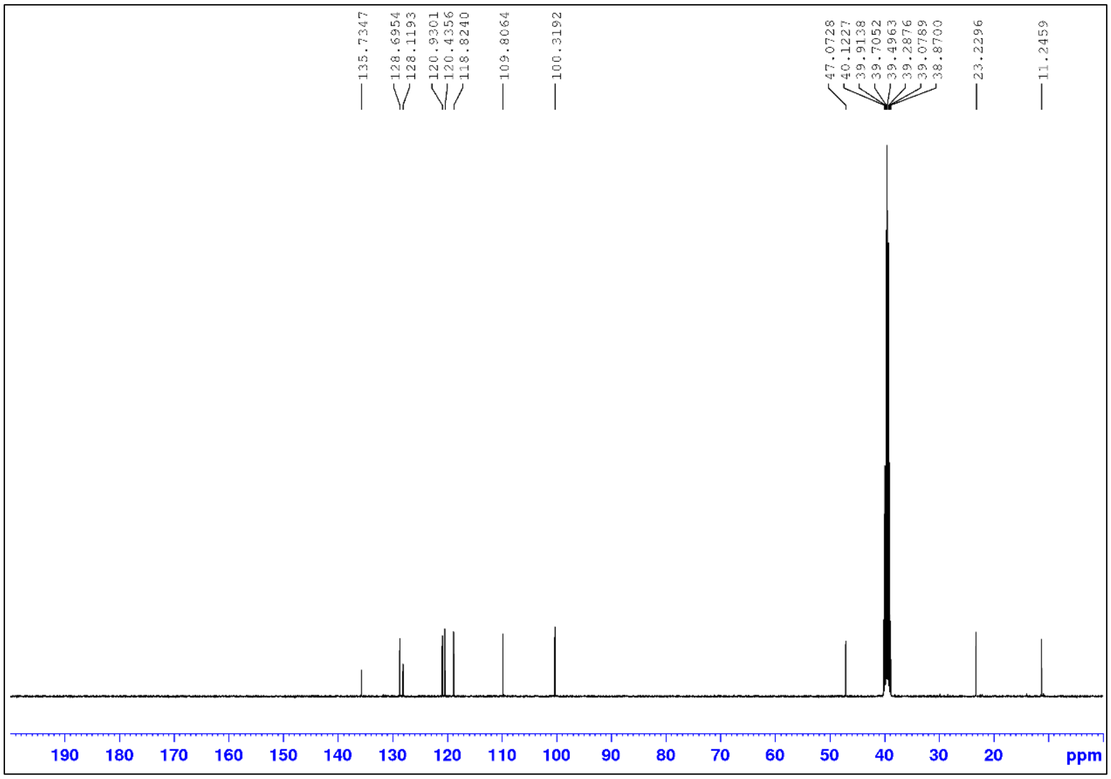

Fig. S38 ^{13}C NMR (DMSO-d_6, 100 MHz) spectrum of 1-propyl-1*H*-indole (**5c**)

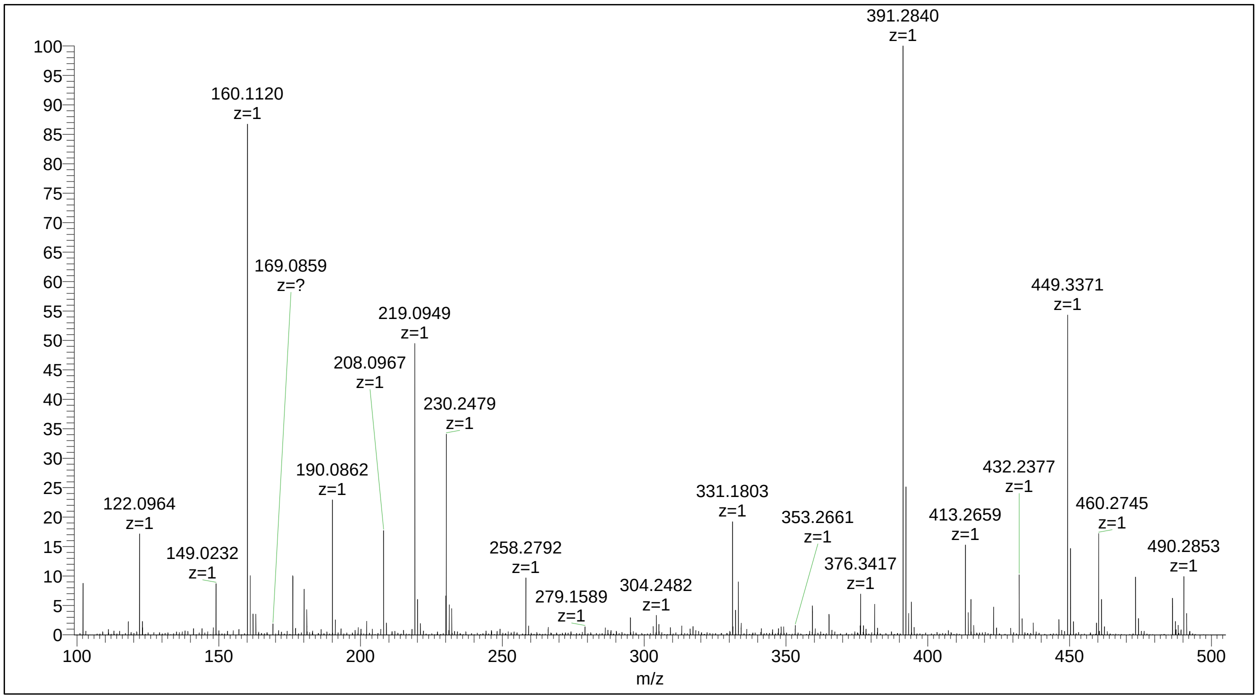

Fig. S39 HRMS (ESI) spectrum of 1-propyl-1*H*-indole (**5c**)

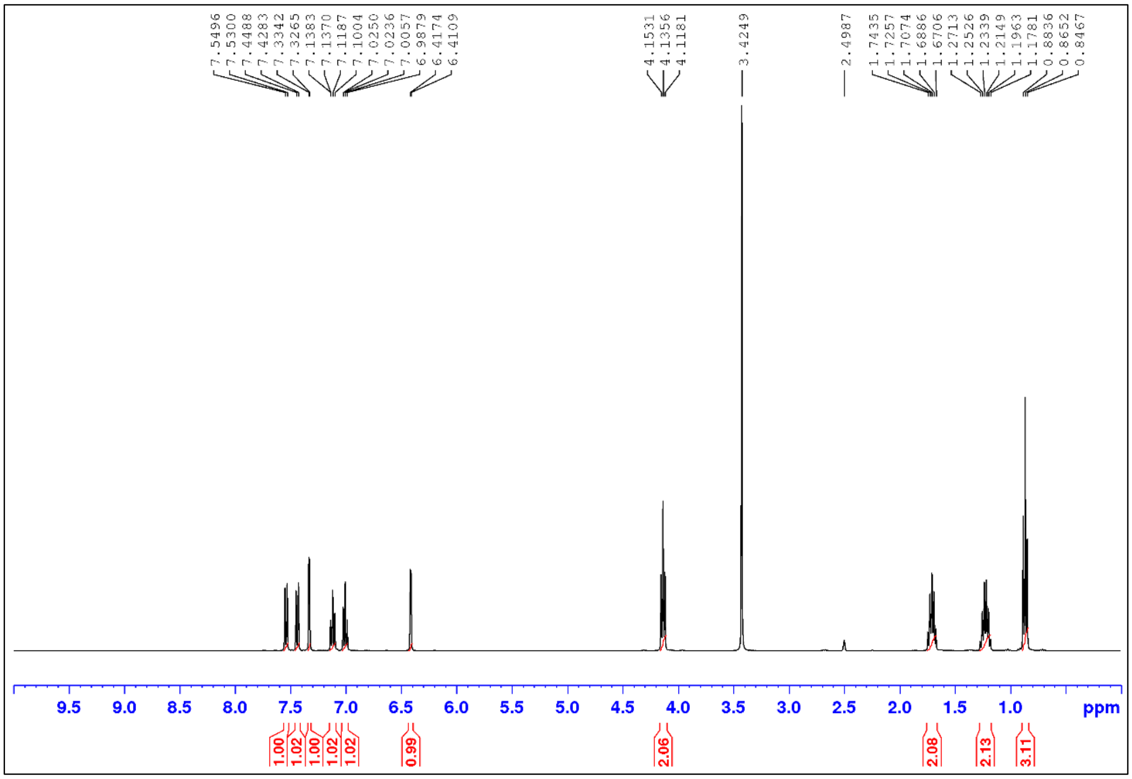

Fig. S40 ^{1}H NMR (DMSO-d_6, 400 MHz) spectrum of 1-butyl-1*H*-indole (**5d**)

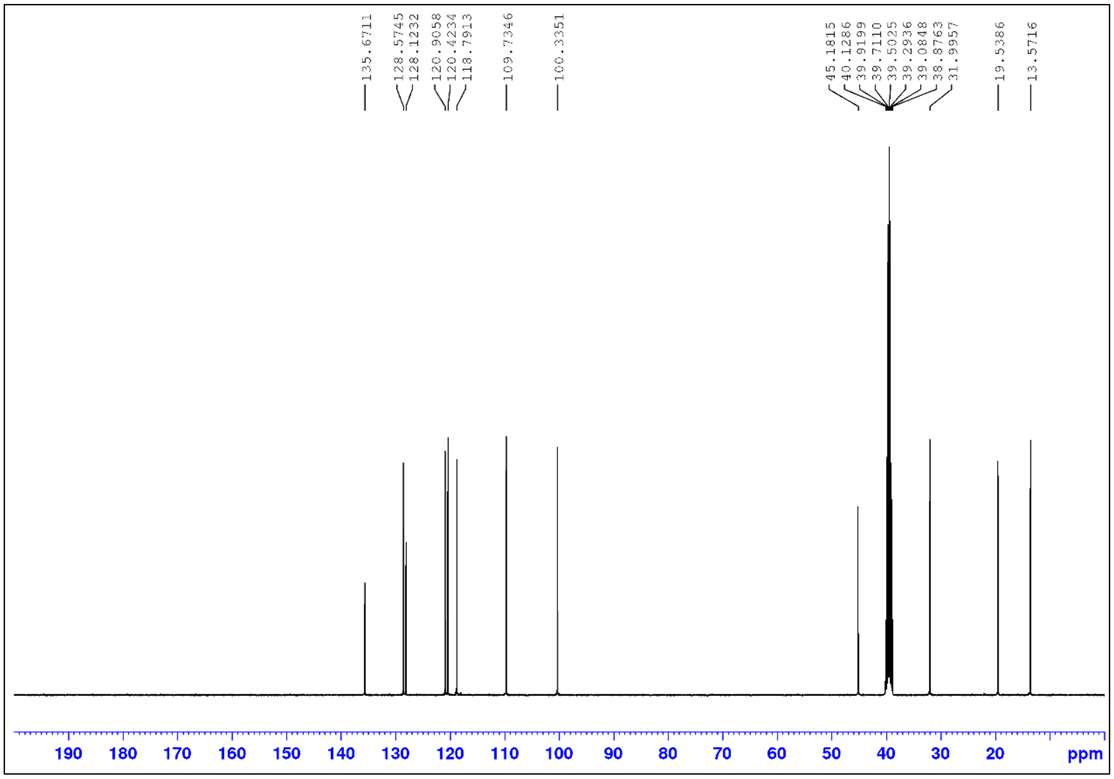

Fig. S41 ^{13}C NMR (DMSO-d_6, 100 MHz) spectrum of 1-butyl-1*H*-indole (**5d**)

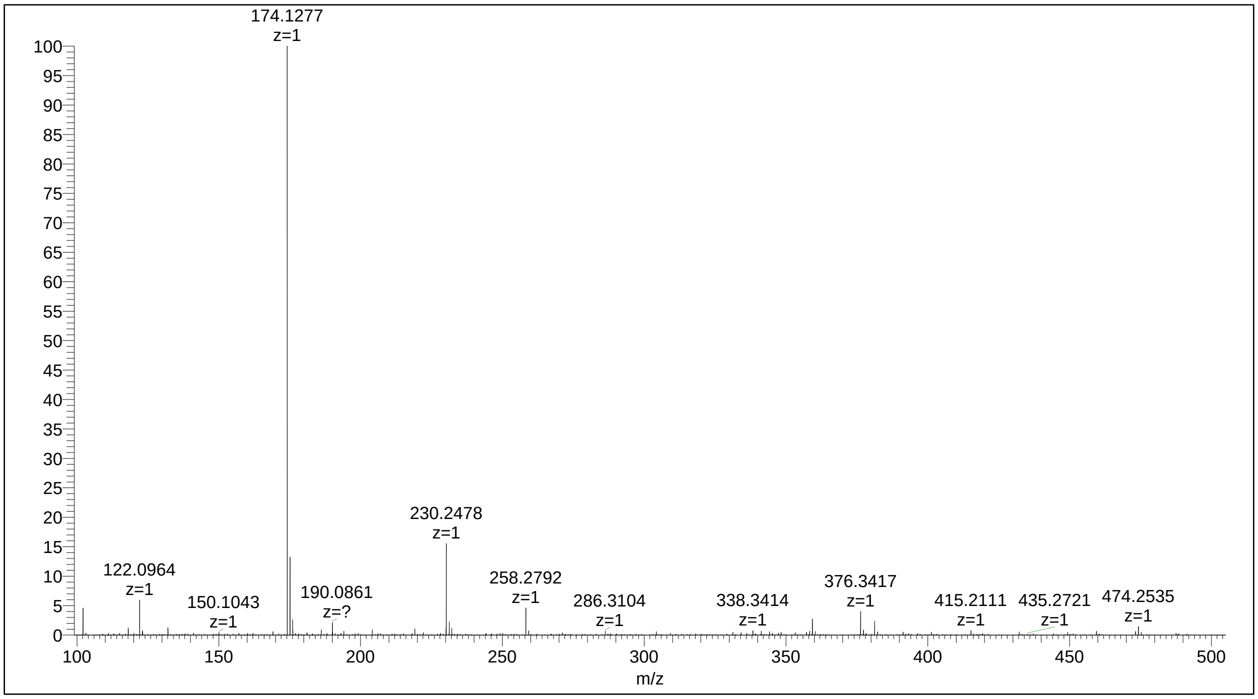

Fig. S42 HRMS (ESI) spectrum of 1-butyl-1*H*-indole (**5d**)

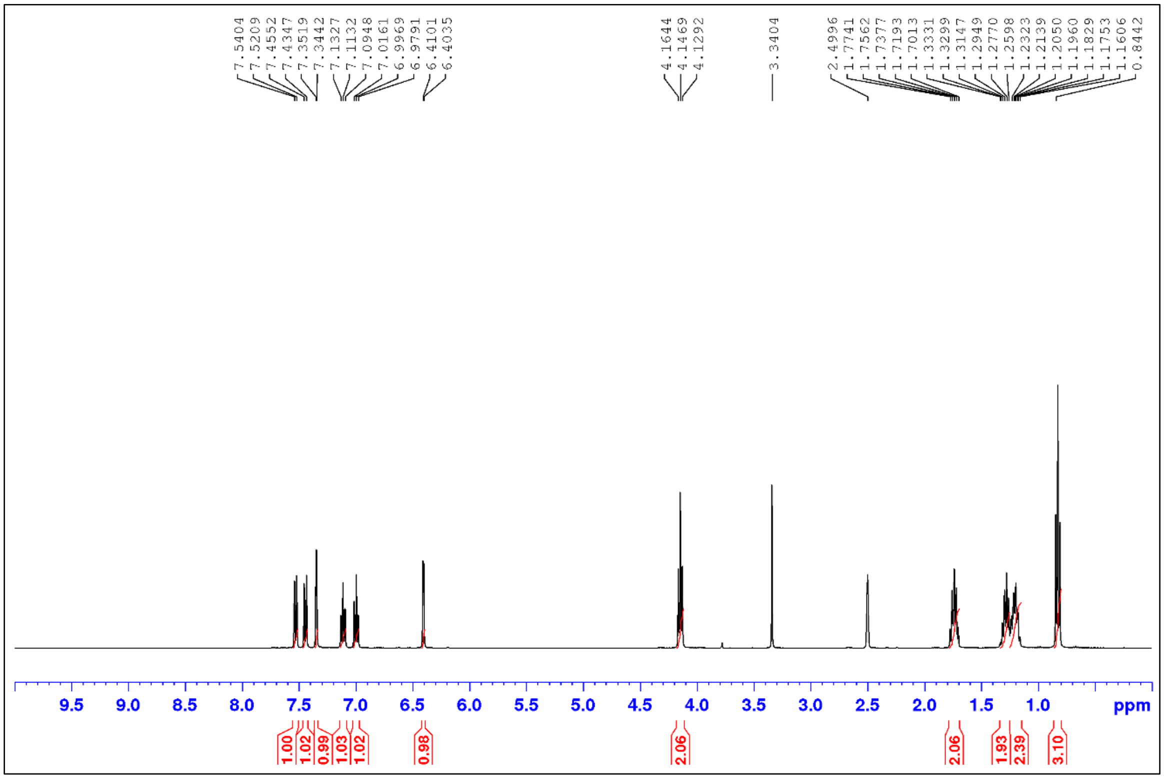

Fig. S43 ^{1}H NMR (DMSO-d_6, 400 MHz) spectrum of 1-pentyl-1*H*-indole (**5e**)

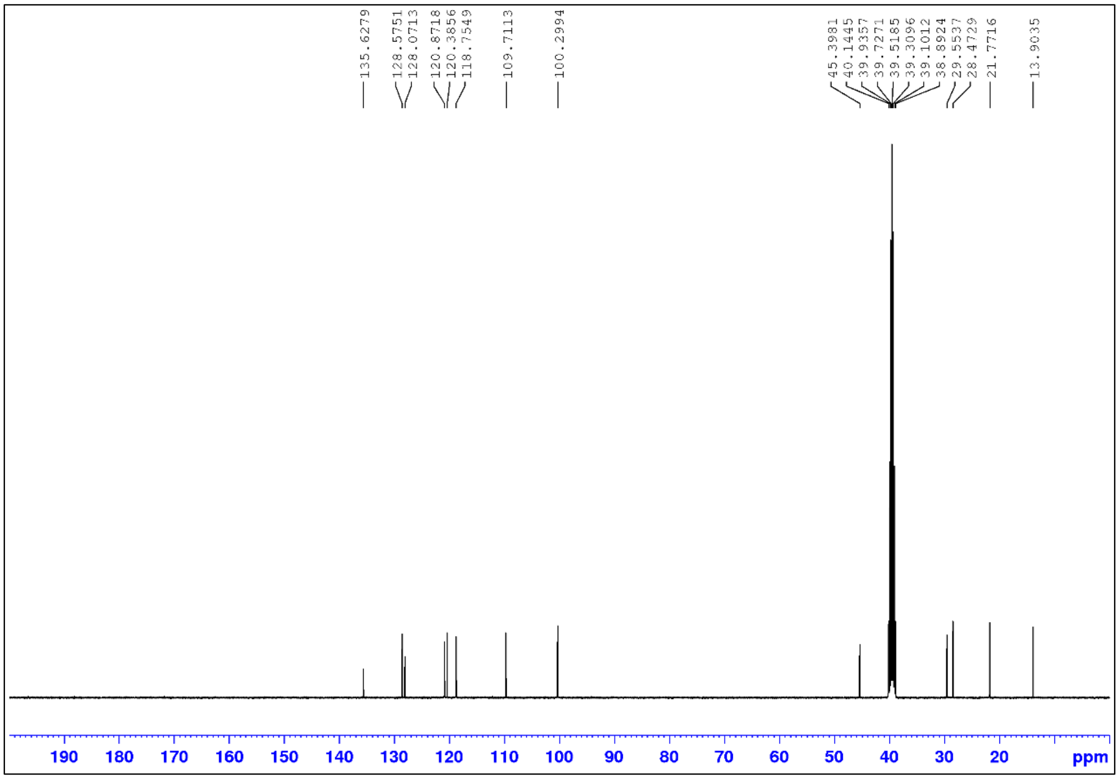

Fig. S44 ^{13}C NMR (DMSO-d_6, 100 MHz) spectrum of 1-pentyl-1*H*-indole (**5e**)

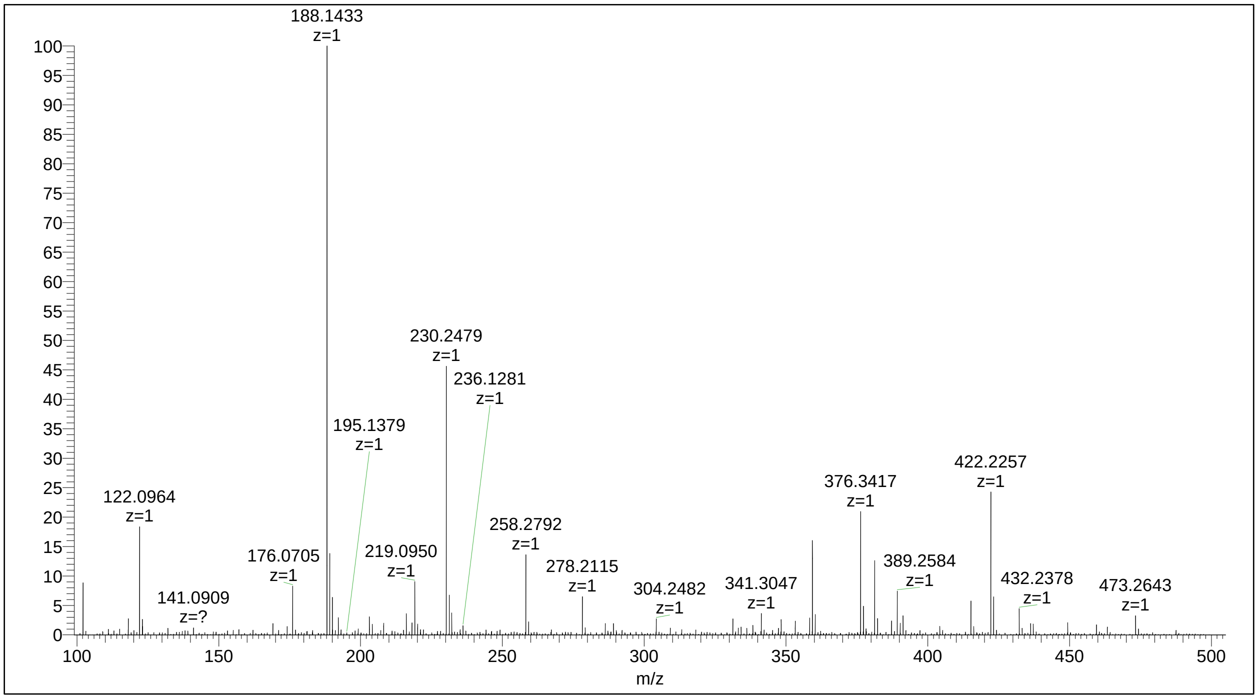

Fig. S45 HRMS (ESI) spectrum of 1-pentyl-1*H*-indole (**5e**)

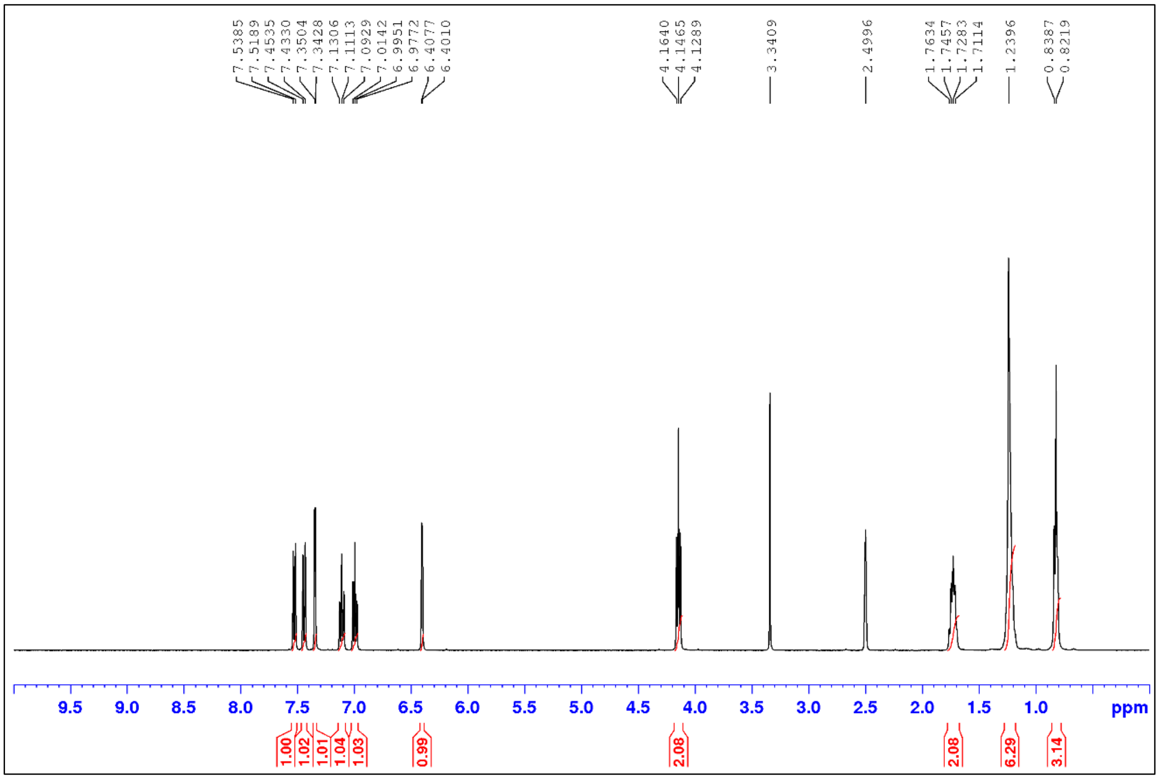

Fig. S46 ^{1}H NMR (DMSO-d_6, 400 MHz) spectrum of 1-hexyl-1*H*-indole (**5f**)

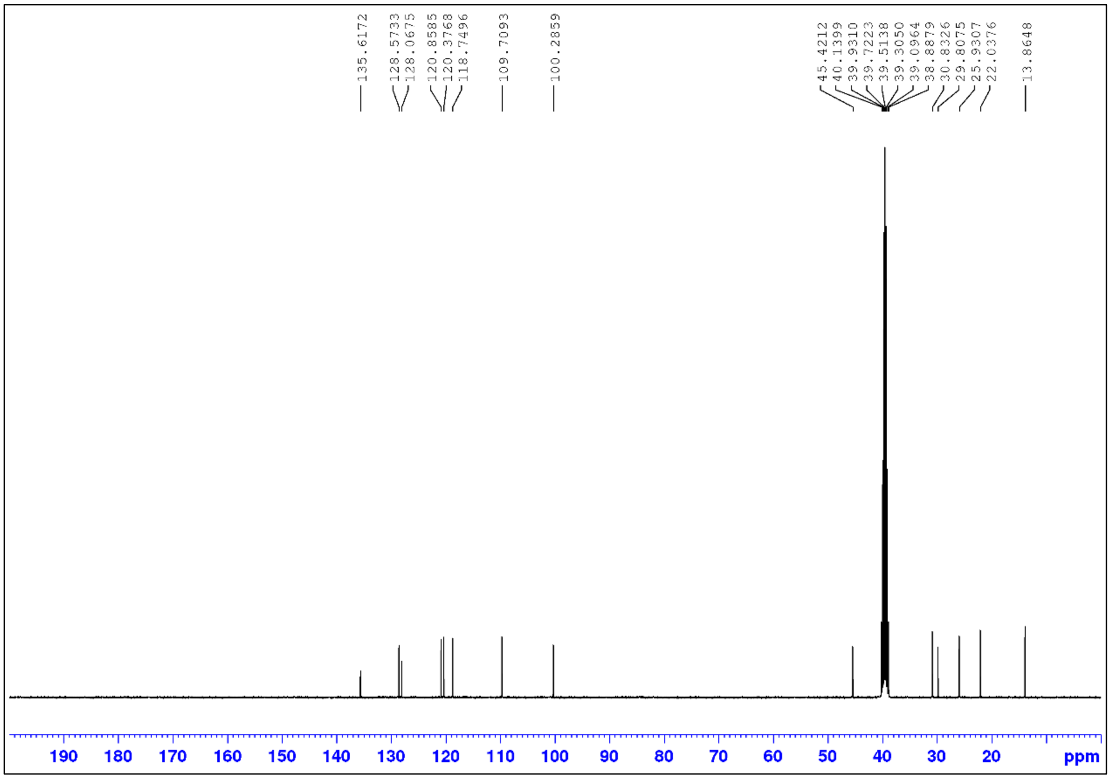

Fig. S47 ^{13}C NMR (DMSO-d_6, 100 MHz) spectrum of 1-hexyl-1*H*-indole (**5f**)

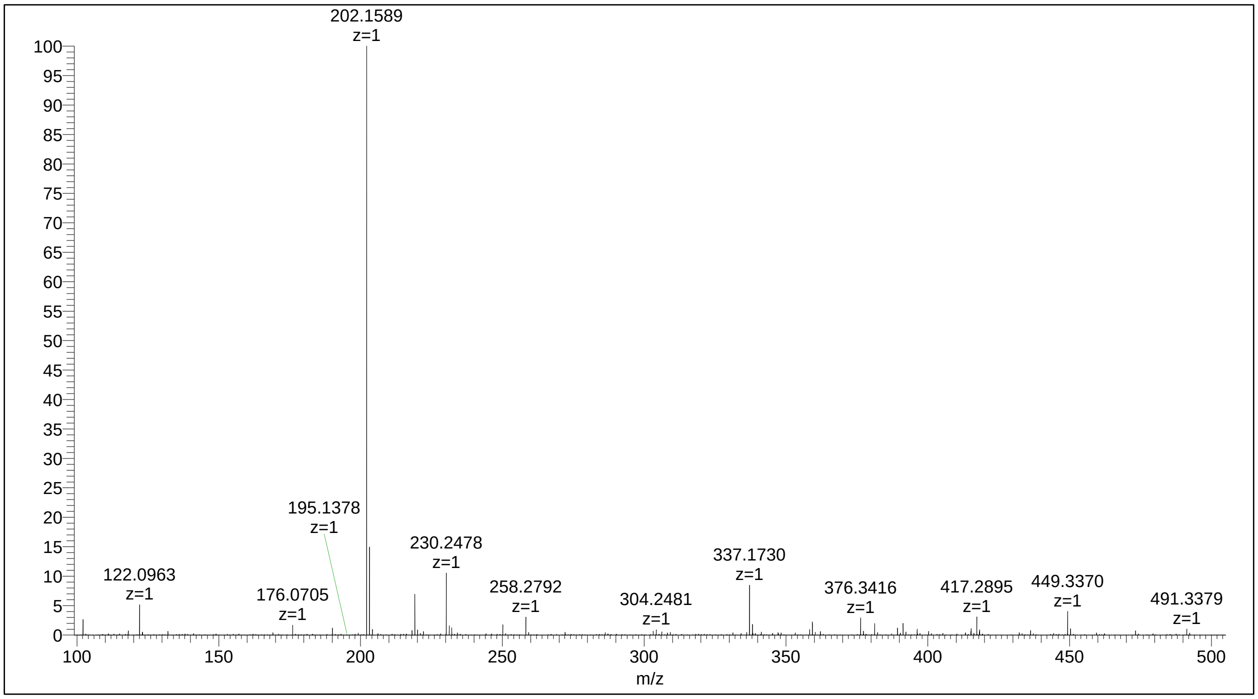

Fig. S48 HRMS (ESI) spectrum of 1-hexyl-1*H*-indole (**5f**)

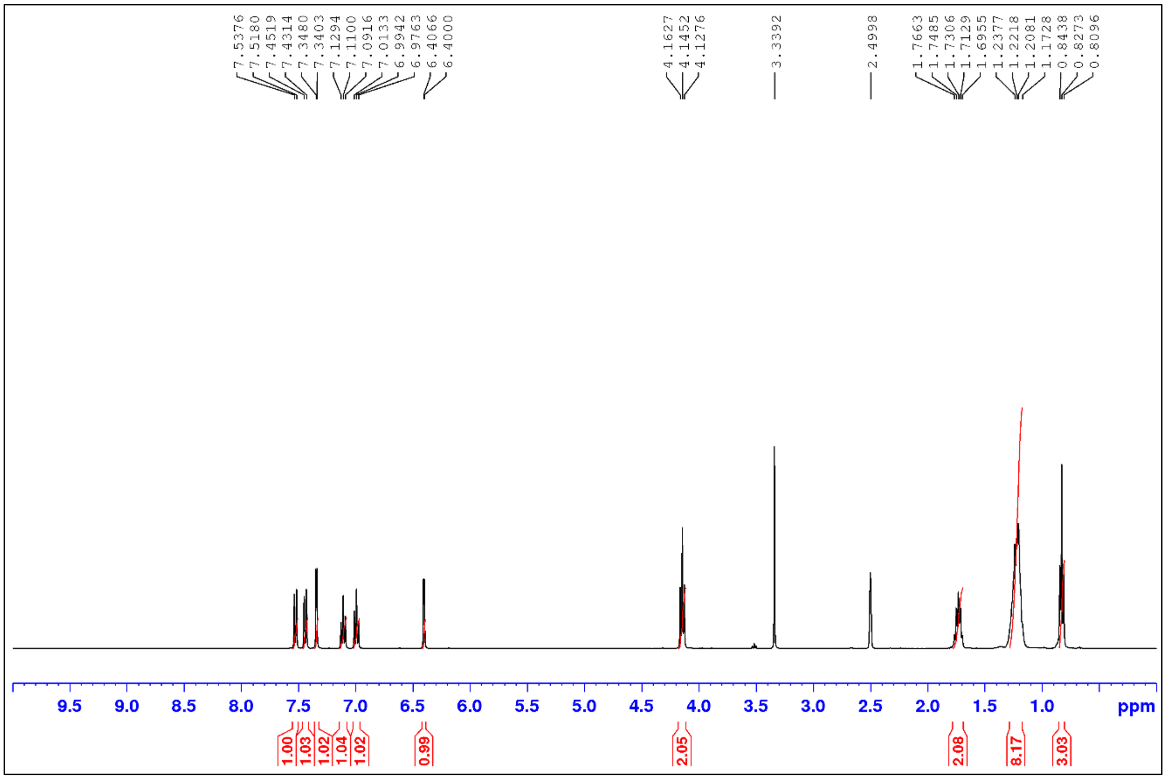

Fig. S49 ^{1}H NMR (DMSO-d_6, 400 MHz) spectrum of 1-heptyl-1*H*-indole (**5g**)

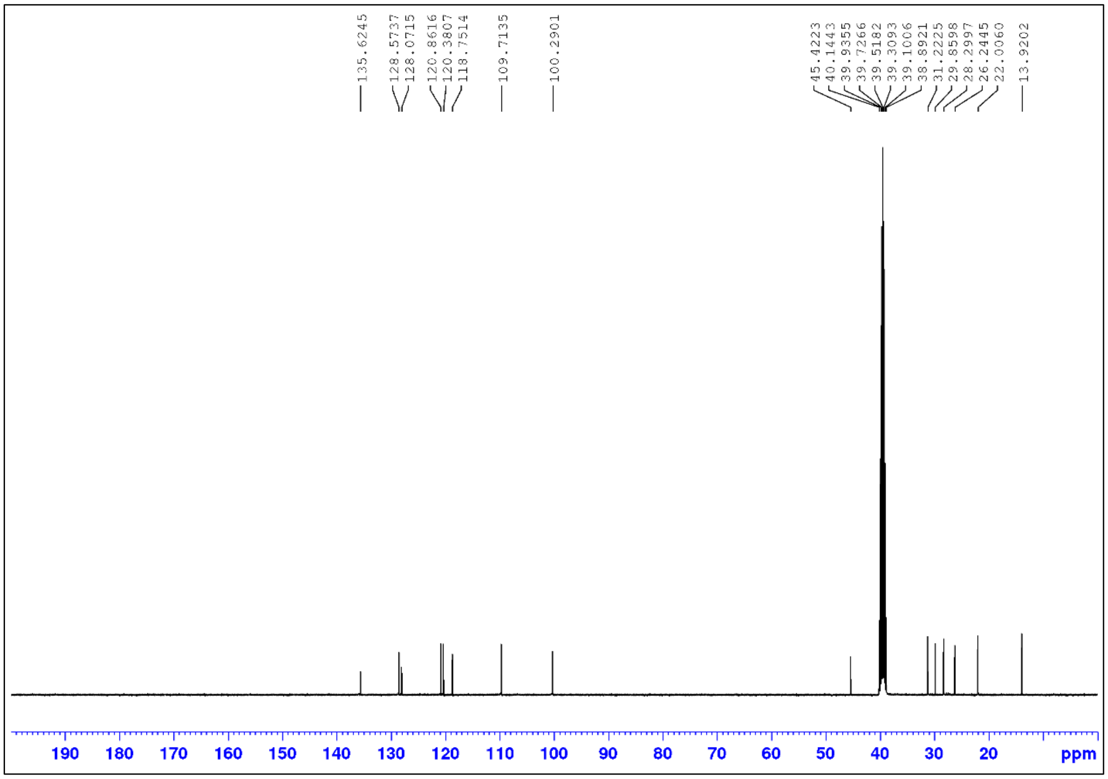

Fig. S50 ^{13}C NMR (DMSO-d_6, 100 MHz) spectrum of 1-heptyl-1*H*-indole (**5g**)

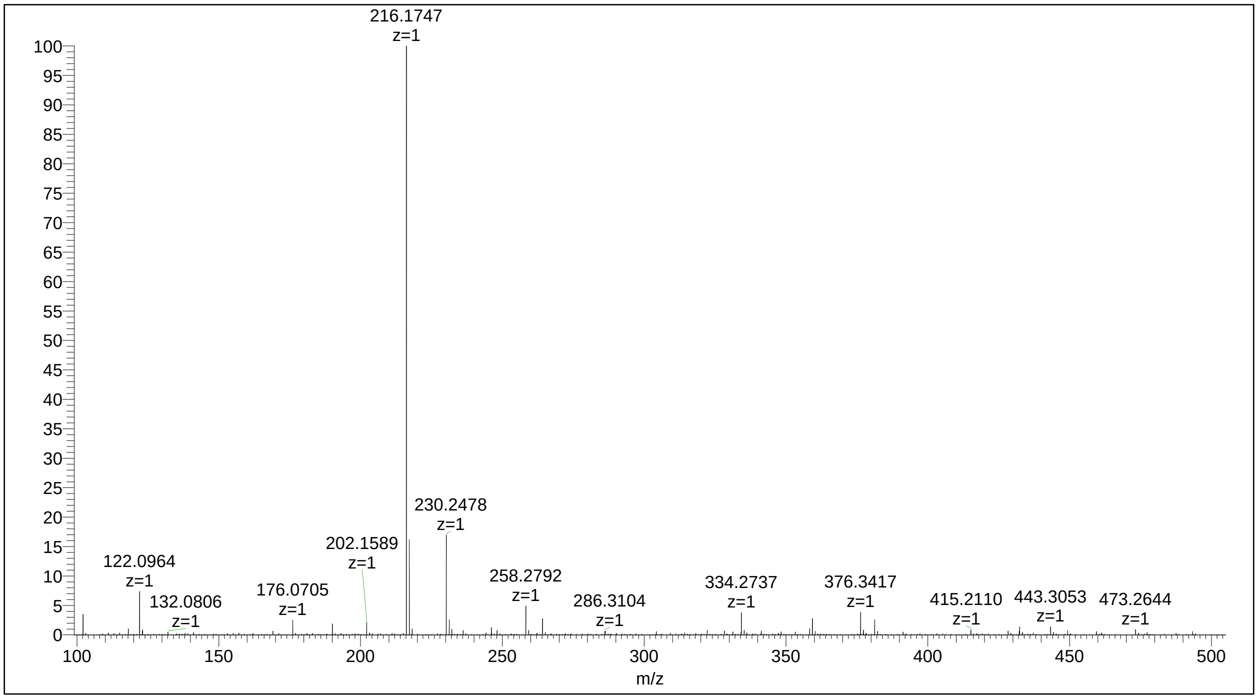

Fig. S51 HRMS (ESI) spectrum of 1-heptyl-1*H*-indole (**5g**)

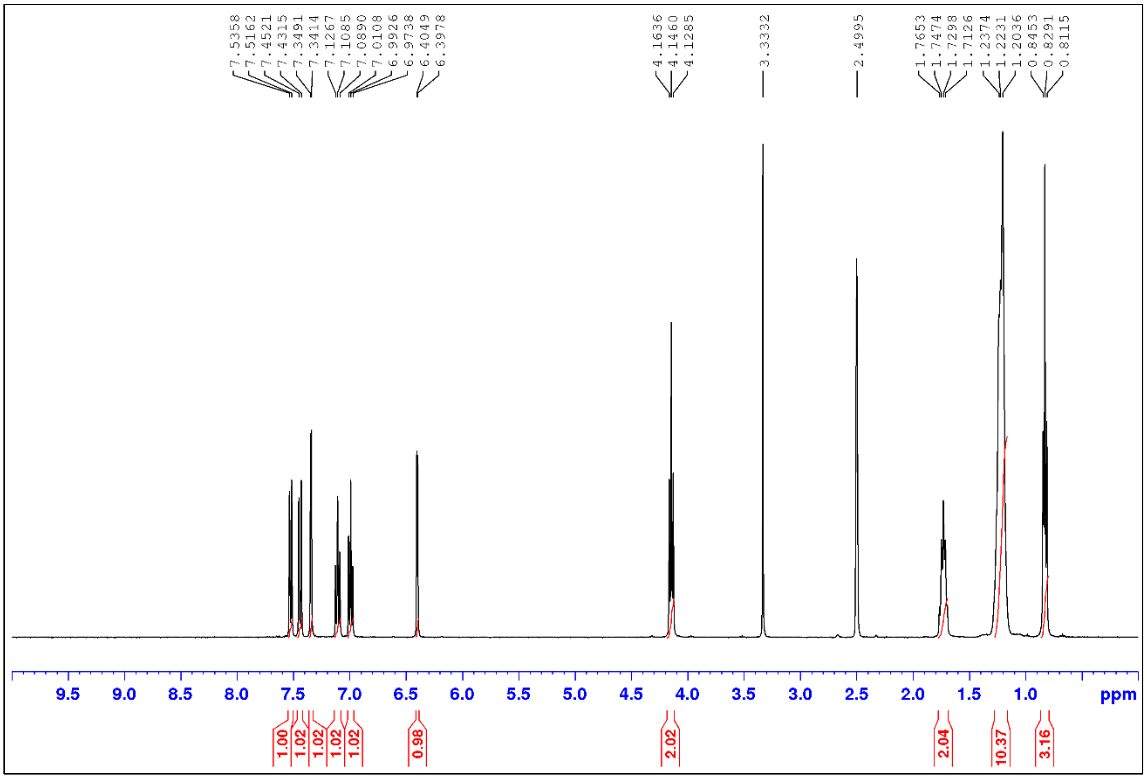

Fig. S52 ^{1}H NMR (DMSO-d_6, 400 MHz) spectrum of 1-octyl-1*H*-indole (**5h**)

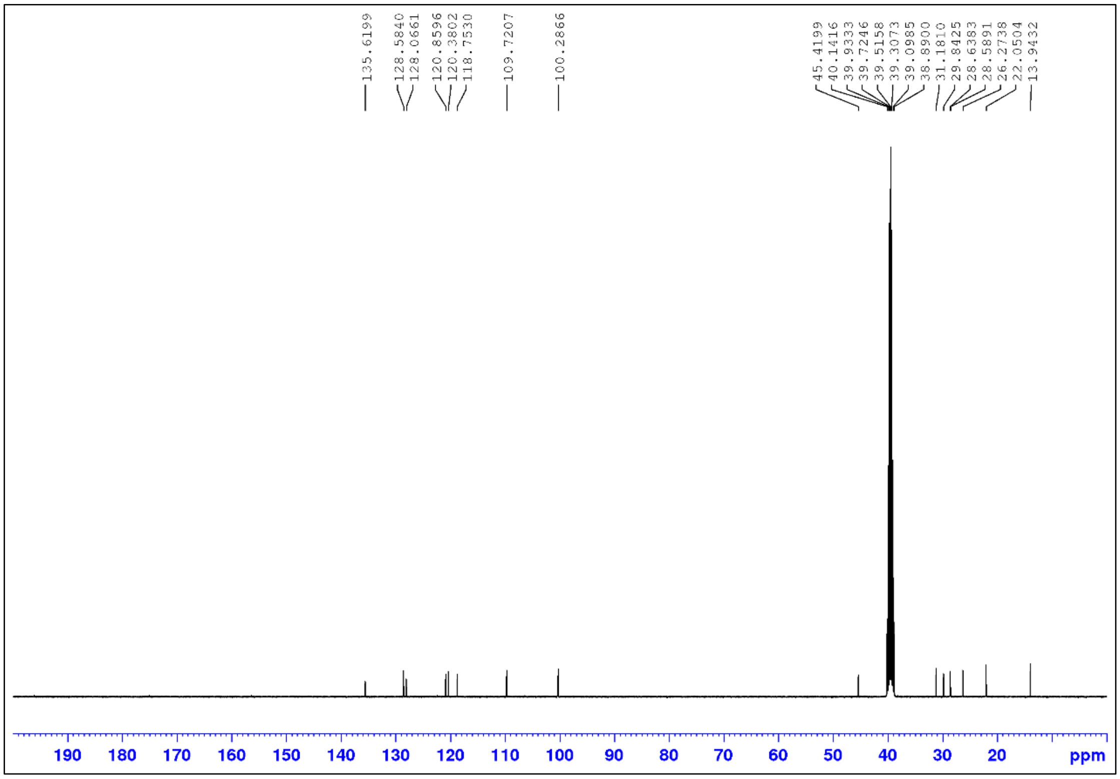

Fig. S53 ^{13}C NMR (DMSO-d_6, 100 MHz) spectrum of 1-octyl-1*H*-indole (**5h**)

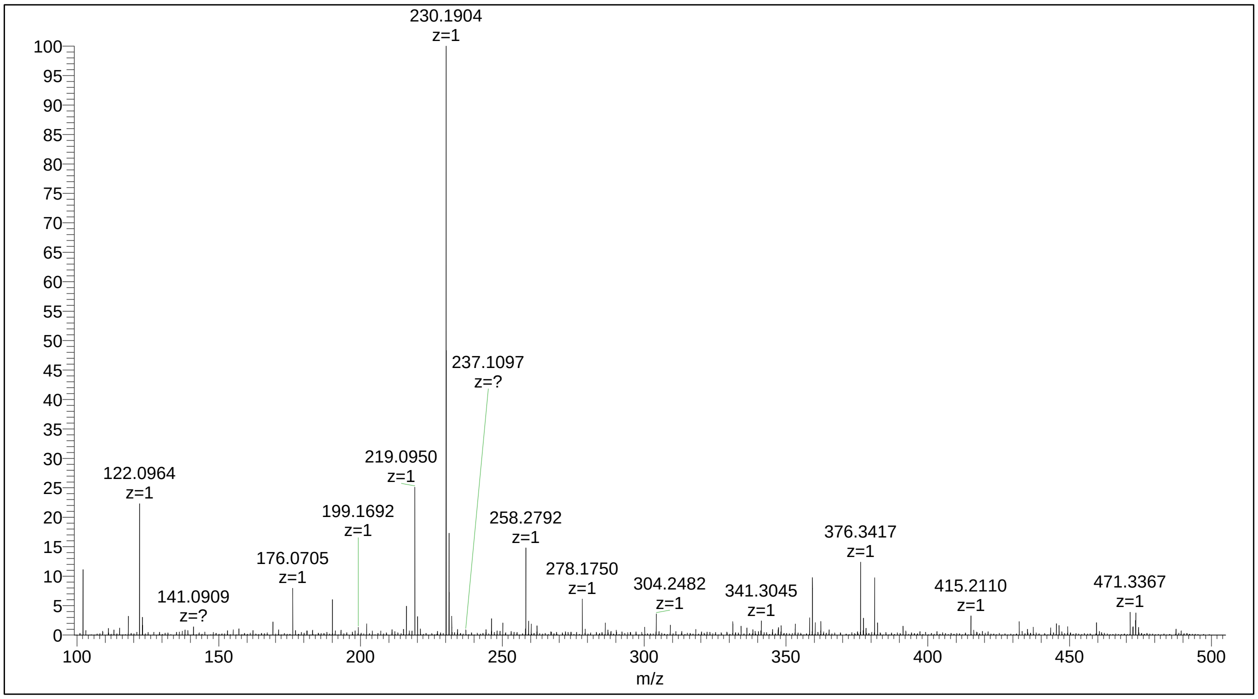

Fig. S54 HRMS (ESI) spectrum of 1-octyl-1*H*-indole (**5h**)

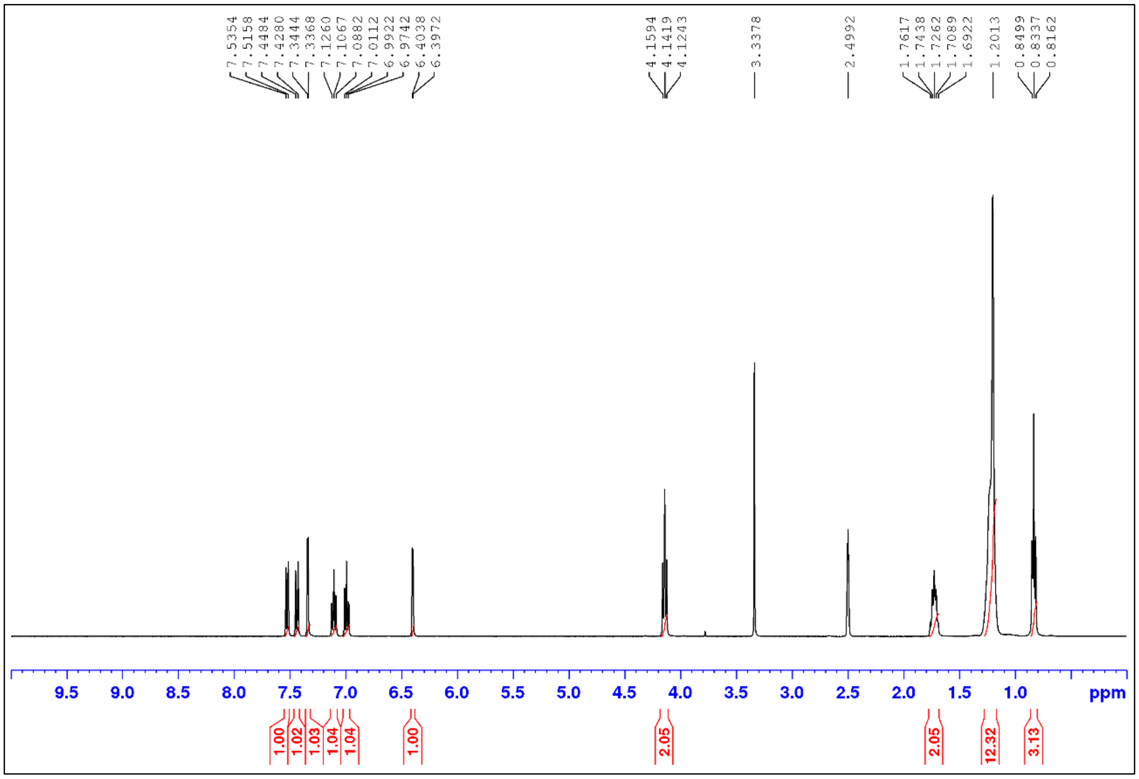

Fig. S55 ^{1}H NMR (DMSO-d_6, 400 MHz) spectrum of 1-nonyl-1*H*-indole (**5i**)

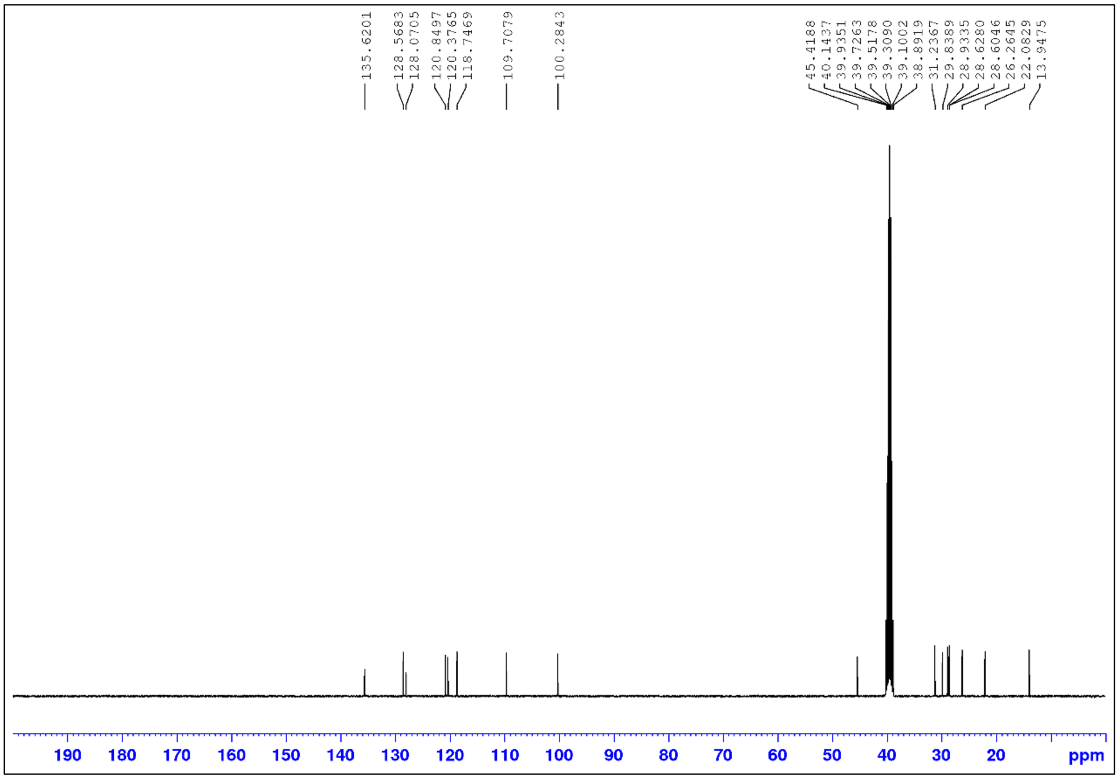

Fig. S56 ^{13}C NMR (DMSO-d_6, 100 MHz) spectrum of 1-nonyl-1*H*-indole (**5i**)

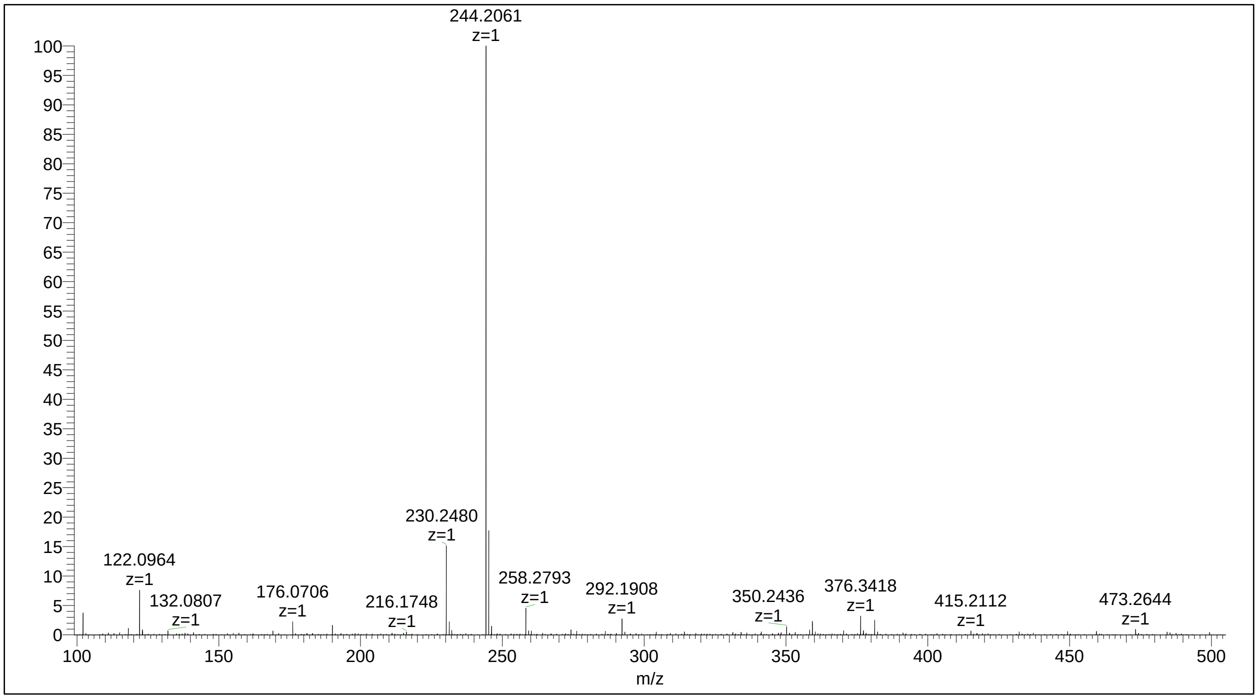

Fig. S57 HRMS (ESI) spectrum of 1-nonyl-1*H*-indole (**5i**)

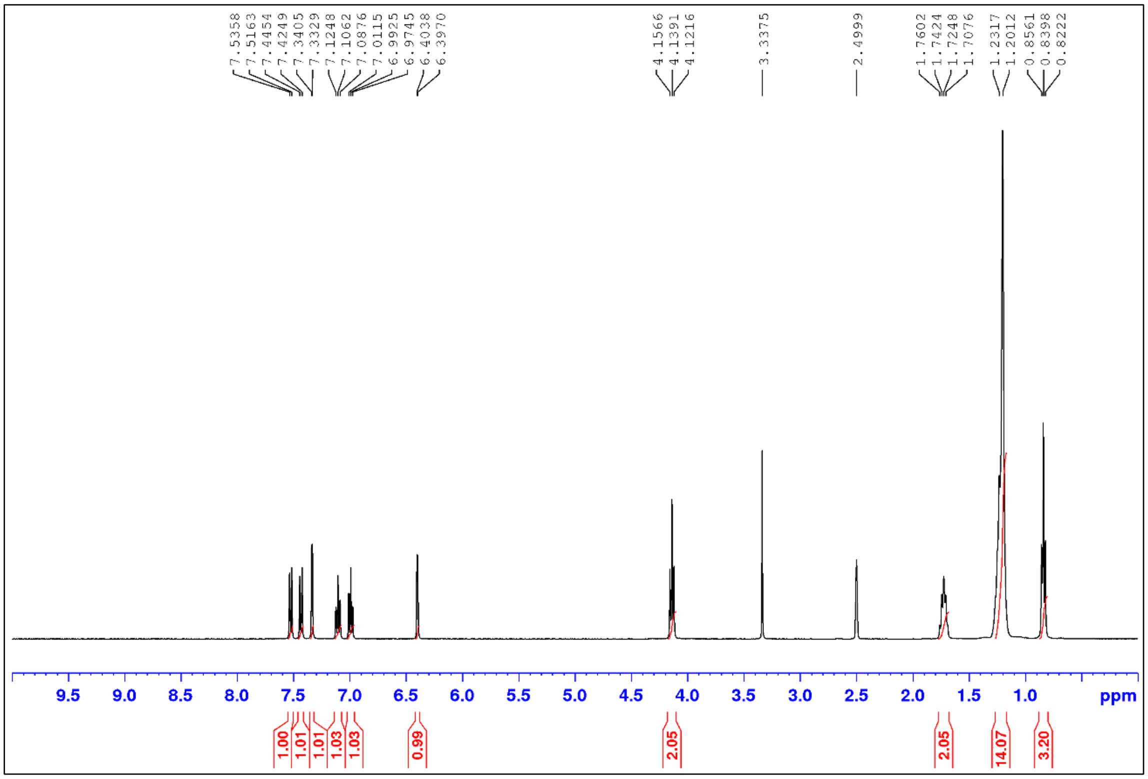

Fig. S58 ^{1}H NMR (DMSO-d_6, 400 MHz) spectrum of 1-decyl-1*H*-indole (**5j**)

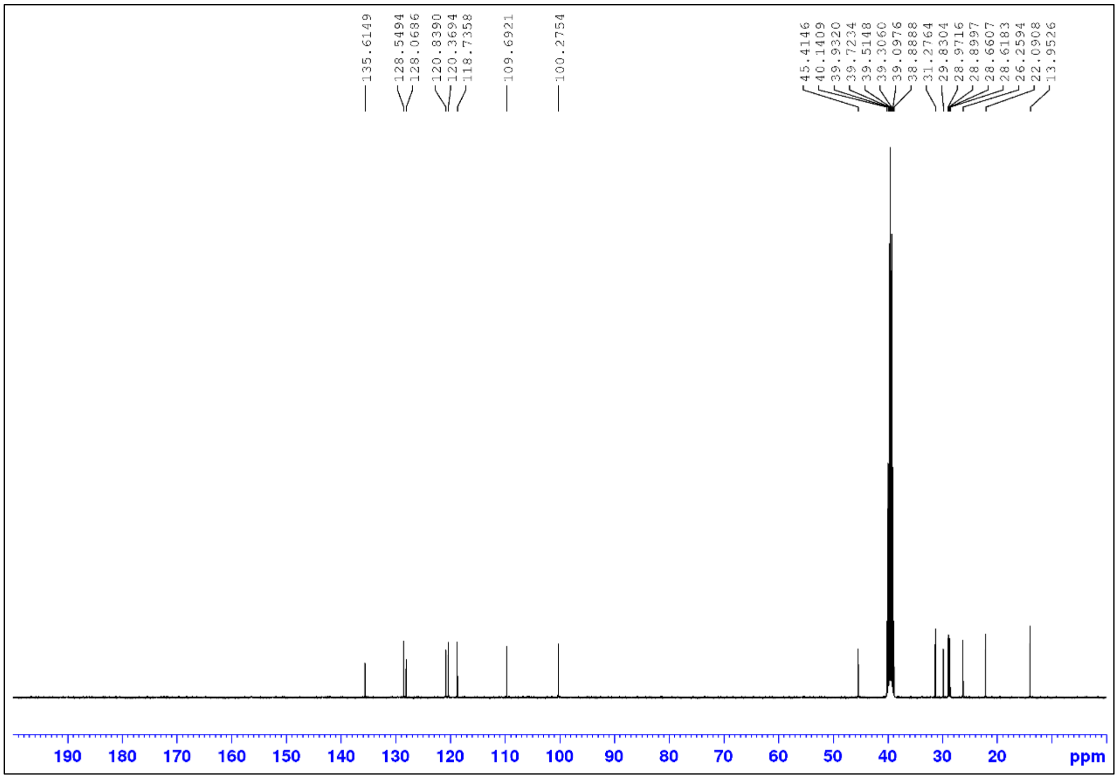

Fig. S59 ^{13}C NMR (DMSO-d_6, 100 MHz) spectrum of 1-decyl-1*H*-indole (**5j**)

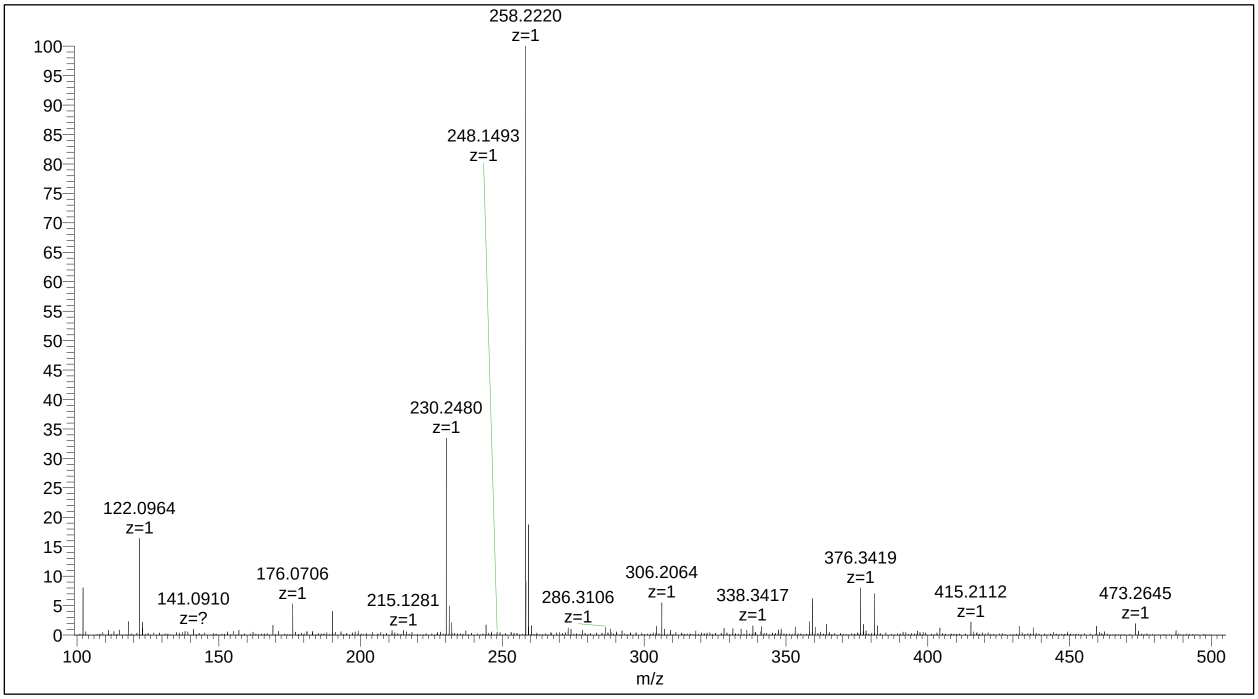

Fig. S60 HRMS (ESI) spectrum of 1-decyl-1*H*-indole (**5j**)

3. List of abbreviations

ACh	acetylcholine
AChE	acetylcholinesterase
AChEI	acetylcholinesterase inhibitor(s)
AD	Alzheimer’s disease
ATC	acetylthiocholine
BBB	blood-brain barrier
BChE	butyrylcholinesterase
BChEI	butyrylcholinesterase inhibitor(s)
BTC	butyrylthiocholine
CAS	catalytic active site
DTNB	5,5′-dithiobis-(2-nitrobenzoic acid)
PAS	peripheral anionic site
SAR	structure-activity relationship
